# Green One-Pot Synthesis of Benzimidazoles from Dinitroarenes in Water Using a Ru-Doped Co-Based Heterogeneous Catalyst

**DOI:** 10.1021/acssuschemeng.5c05806

**Published:** 2025-09-08

**Authors:** José Luis del Río-Rodríguez, Silvia Gutiérrez-Tarriño, María Asunción Molina, Lucy Costley-Wood, Chiara Falcini, Andrew M. Beale, Pascual Oña‑Burgos

**Affiliations:** † Instituto de Tecnología Química, 16778 Universitat Politècnica de València-Consejo Superior de Investigaciones Científicas (UPV-CSIC), Avda. de los Naranjos s/n, Valencia 46022, Spain; ‡ Department of Chemistry, 4919 University College London, 20 Gordon Street, London WC1H 0AJ, UK; § Research Complex at Harwell, Rutherford Appleton Laboratories, Harwell Science and Innovation Campus, Harwell, Didcot OX11 0FA, UK; ∥ Departamento de Química Orgánica, Universidad de Sevilla, c/Profesor García González 1, Sevilla 41012, Spain

**Keywords:** heterogeneous catalysis, 1,2-dinitroarenes, cobalt carbon catalysts, ruthenium doping, hydrogenative coupling, benzimidazoles, one-pot reactions

## Abstract

A novel methodology is presented for preparing non-noble metal-based heterogeneous catalysts doped with low amounts of noble metals, yielding highly active and reusable materials for sustainable organic transformations. A new **Co–Ru@C** catalyst was developed by incorporating small ruthenium nanoparticles (1 wt %) and cobalt nanoparticles (10 wt %) on Vulcan carbon via pyrolysis of a ruthenium-doped cobalt metal–organic framework supported on carbon (2D-Co­(Ru)­MOF/C). Advanced characterization (*in situ* PXRD and XAS, HAADF-STEM, HRTEM) confirmed strong Co–Ru interactions, enhancing catalytic activity. The catalyst was evaluated in the one-pot reductive amination of dinitrobenzenes to benzimidazoles using molecular hydrogen and water, which is in line with green chemistry. The synergistic effect between Co and Ru enabled quantitative product yields under significantly milder reaction conditions than the existing ones. In addition, ruthenium was found to facilitate hydrogen activation in the bimetallic material compared with its undoped **Co@C** counterpart. Regarding stability, the **Co–Ru@C** catalyst retained its activity over at least five consecutive cycles without metal leaching or structural degradation. This material demonstrated a broad substrate scope, affording over 20 functionalized benzimidazoles in high yields. Half-gram-scale syntheses of commercial Diabazole and Fuberidazole further validated the scalability of this approach.

## Graphical abstract

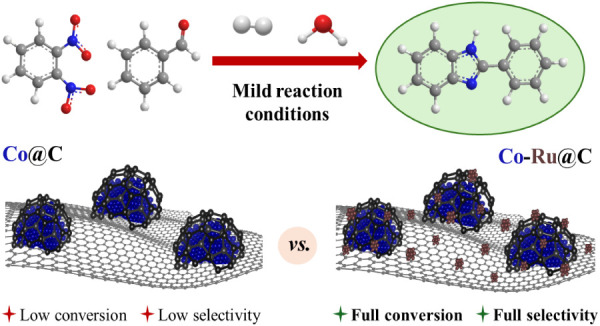

## Introduction

Heterocyclic systems are an important class of organic compounds.^1–3^ They have significant roles in pharmaceutical chemistry, biochemistry, and other scientific disciplines.^4^ Nitrogen-containing heterocycles are the most common heterocyclic compounds in nature. Among them,^5^ benzimidazole stands out in medicinal chemistry for its extensive pharmacological and biological activities including anticancer,^6^ anti-inflammatory,^7^ antiviral,^8^ antimicrobial,^9^ antiprotozoal,^10^ antimalarial,^11^ and antiparasitic agents,^12^ among others. Benzimidazole derivatives are also recognized as intermediates in synthetic chemistry and as ligands in asymmetric catalysis.^13^ Notably, this core structure is present in many commercially available drugs, including Mebendazole, an antiparasitic medication, Luxabendazole, a chemotherapeutic agent, Omeprazole, used for treating specific stomach and esophagus issues and Thiabendazole, an antifungal and antiparasitic agent.^14^

Consequently, the synthesis of benzimidazoles has garnered increasing interest from synthetic organic chemists and biologists.^15^ The most common methods for the synthesis of 2-substituted benzimidazoles involve the condensation of o-phenylenediamine with aldehydes, carboxylic acids or their derivatives, using various catalysts and reagents.^14^ However, these traditional approaches have several drawbacks, including high waste production, challenges in catalyst reuse, the use of inorganic acids, nonsustainable solvents and low atom economy.^16^ To address these shortcomings, nanomaterials and nanocatalysts have emerged as superior alternatives for various organic transformations, outperforming conventional materials in almost all types of catalytic organic reactions.^17^ In recent years, there has been growing interest in harnessing the high activity and selectivity of nanocatalysts to develop greener, more efficient, and waste-minimized processes.^18,19^ Recently, a synthetic one-pot strategy that involves reductive amination using dinitroarenes and aldehydes as starting materials has been reported (Scheme 1). In this context, Cao et al. have faced this one-pot reaction using an Au@TiO_2_ catalyst and HCOOH as the hydrogen source, achieving high conversion of the reactants with good selectivity (87%).^20^ Sorribes et al. also reported a sulfur-deficient molybdenum disulfide material active for this one-pot reaction with molecular H_2_, but using toluene as a reaction medium and high metal loading (19.3% Mo), achieving total conversion and high selectivity (94%).^21^ Finally, our group has recently reported a Co–N,P doped nanoparticles supported on carbon achieving full conversion and chemoselectivity, and stability toward the reduction of dinitroarenes using water as solvent and hydrogen as reductant.^22^ The focus must now be on developing a tailored catalyst that can efficiently perform this reaction, with total conversion and selectivity, under milder conditions than so far achieved.

**Scheme 1:**
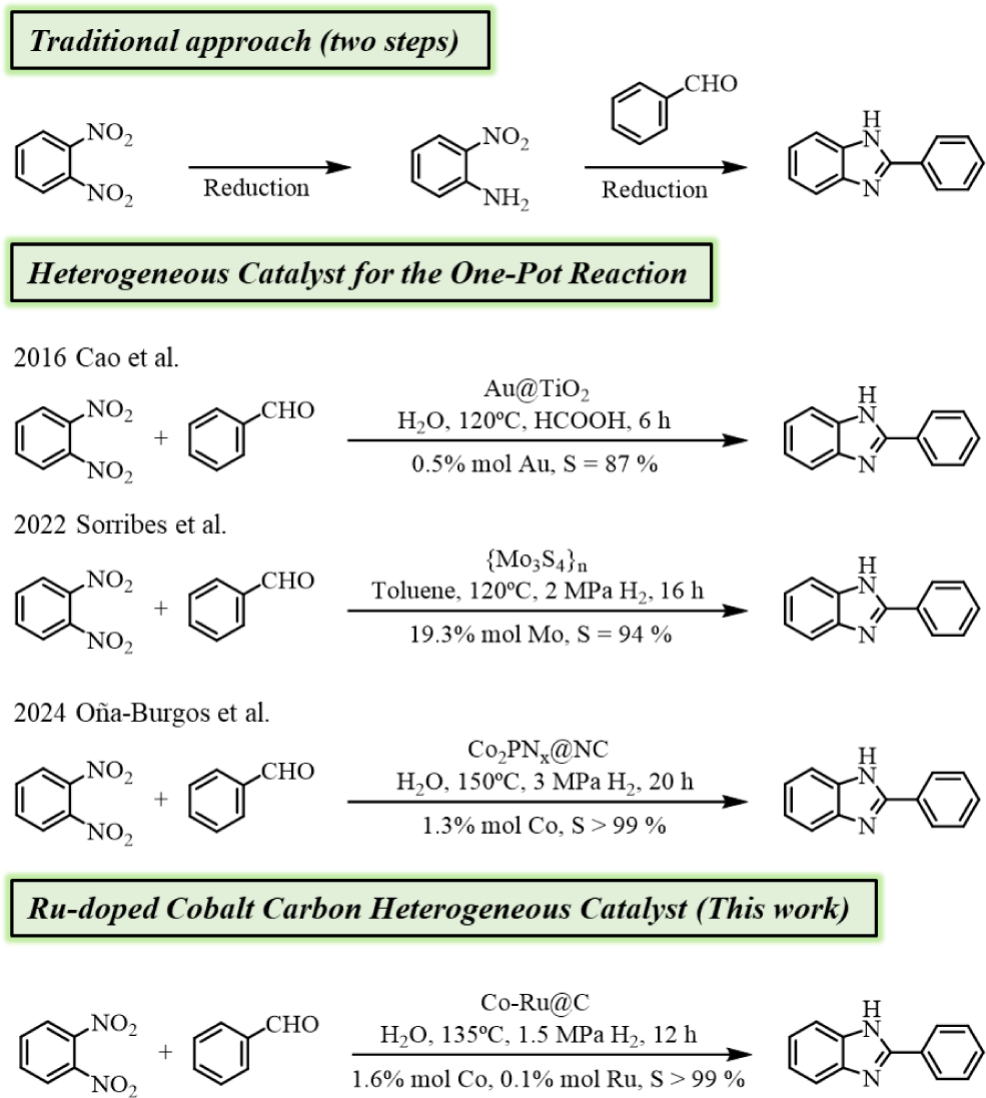
Overview of Reported Heterogeneous Catalysts for the One-Pot Synthesis of Benzimidazoles from 1,2-Dinitroarenes and Comparative Analysis with This Work

The literature clearly shows that the properties of nanomaterials can be tailored by modifying their size, shape, and composition. Many advanced strategies have been proposed to boost the catalytic performance of non-noble metal-based materials. Among these strategies, heteroatom doping is widely recognized as a promising approach for greatly improving catalytic performance due to its multiple advantages.^23–27^ In this context, heteroatom doping can modify the electronic structure of the catalyst, optimizing the adsorption energy of reaction intermediates.^28–31^ This enables precise tuning of catalytic properties. Many catalysts based on elements such as Fe, Co, Ni, Mn, and Cu have been doped into the catalysts to tailor their chemical properties. Notably, incorporating low amounts of noble metals (Pd,^32,33^ Pt,^34,35^ Ru,^36–38^ Rh,^39,40^ and Ir^41,42^ ) into non-noble metal nanocatalysts has garnered significant attention as an effective method for optimizing catalytic properties. On the one hand, noble metal doping can modulate the electronic structure to optimize the adsorption strength of intermediates,^43^ and significantly improve the intrinsic activity of the active species.^44^ On the other hand, the introduced noble metals have been shown to function as active centers, creating a strong synergy effect that enhances the catalytic performance.^45,46^

Drawing on these advantages, considerable effort has been devoted to developing catalysts lightly doped with small amounts of noble metals that can ease reaction conditions by markedly lowering key parameters (pressure, temperature and time), while still achieving high conversion, excellent selectivity toward the desired product, and sustained stability over repeated uses. In this sense, in the present work, we have synthesized a novel **Co–Ru@C** material, containing about 10 wt % cobalt as the major metallic compound and roughly 1 wt % ruthenium. The evolution of metal precursors during pyrolysis was tracked in real time using *in situ* PXRD and XAS, revealing both structural changes and metal speciation. This combined method is crucial for understanding the transformation of MOF-derived catalysts into their active phases and for detecting alloy formation or intermediate species that may impact catalytic performance. In fact, **Co–Ru@C** displayed outstanding activity in the one-pot synthesis of benzimidazoles via reductive coupling of dinitroarenes with aldehydes under exceptionally mild conditions when compared with its monometallic **Co@C** counterpart. Reusability tests and ICP measurements further confirmed its stability and reliability over successive cycles. Moreover, more than 20 substituted dinitroarenes and benzaldehydes were examined, affording very good yields across a wide array of benzimidazoles and including products of high industrial relevance.

## Results and Discussion

### Synthesis and Characterization of the Catalysts

**Co@C** and **Co–Ru@C** catalysts were synthesized using a two-step procedure (Figure 1). First, a well-defined 2D-CoMOF precursor supported on carbon (2D-CoMOF/C) was obtained following a reported procedure,^47^ including carbon in the one-pot synthesis. Briefly, all reactants were added to a stainless-steel autoclave, which was sealed for 9 days at 150 °C in solvothermal, dynamic conditions. To synthesize the precursor of the bimetallic material, **Co–Ru@C**, RuCl_3_ was also included in this step of the synthesis, obtaining 2D-Co­(Ru)­MOF/C. In the second step, the filtered and dried precursors were pyrolyzed under a nitrogen atmosphere at 800 °C for 2 h (heating rate: 25 °C/min), obtaining the **Co@C** and **Co–Ru@C** catalysts. The metal content was determined by X-ray fluorescence (XRF), **Co@C** contains 11.1 wt % Co, while **Co–Ru@C** has a similar metal loading, with 9.6 wt % Co and 0.8 wt % Ru.

**Figure 1:**
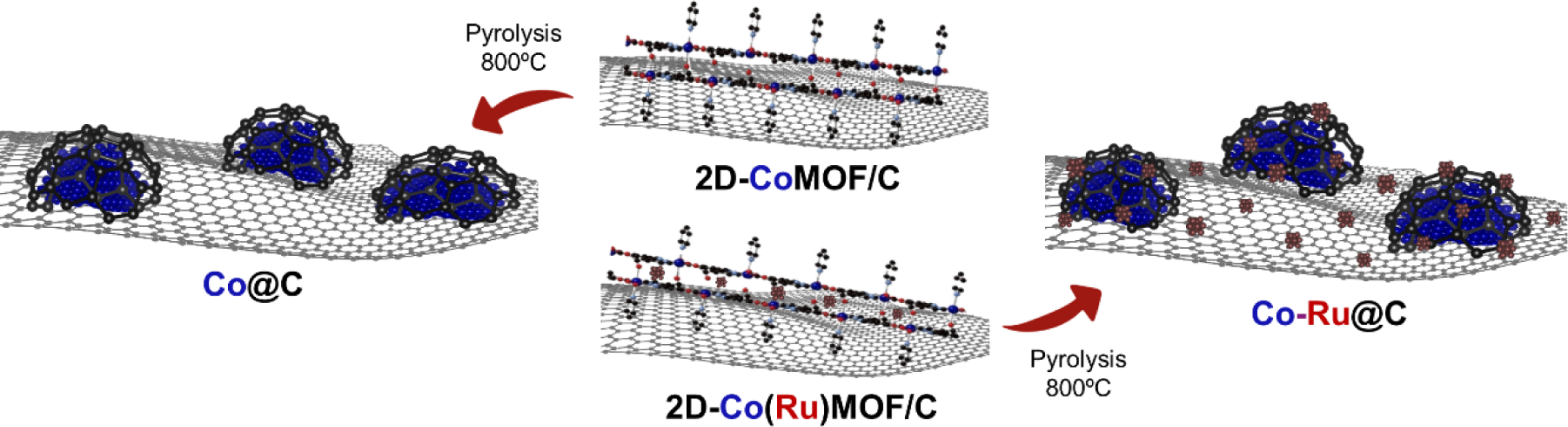
Schematic synthesis of **Co@C** and **Co–Ru@C**.

First, the synthesized precursors were characterized to confirm the formation of well-defined MOFs and their effective support on carbon. Powder X-ray diffraction (PXRD) patterns displayed the characteristic reflections of the 2D-CoMOF (from 8° to 40°),^47^ which remained visible even in the presence of the carbon support (Figure S1). A similar diffraction pattern was observed in the bimetallic 2D-Co­(Ru)­MOF/C, indicating that the incorporation of the second metal did not disrupt the layered structure. Field emission scanning electron microscopy (FESEM) images of the carbon-supported MOFs revealed the typical hexagonal morphology of 2D-CoMOF crystals coated with Vulcan carbon (Figure S2), further validating the structural integrity of the materials.^47^ X-ray absorption spectroscopy (XAS) at both the Co and Ru K-edges was employed to assess the oxidation states and local coordination environments of the metal centers. The Co K-edge XANES spectra of both materials showed features characteristic of Co­(II) in a coordination environment consistent with its incorporation into the MOF framework, as confirmed by comparison with a Co­(Ac)_2_ reference (Figure 2a). This was further supported by the corresponding EXAFS spectra (Figures 2b, S3 and Table S1), which showed a low signal of the second-shell contribution in the Fourier transform (FT), ruling out the presence of large Co aggregates. Similarly, the Ru K-edge XANES spectrum of the bimetallic 2D-Co (Ru)­MOF/C (Figure 2c) displayed features indicative of Ru­(II), as confirmed by comparison with the [Ru­(bpy)_3_]­Cl_2_ reference. The corresponding EXAFS data (Figure 2d) do not show an evident Ru–Ru contribution in the FT, as it would normally be observed in RuO_2_ or similar crystalline structures. These findings suggest that Ru is either well-dispersed within the MOF matrix or exists as small, amorphous species.

**Figure 2:**
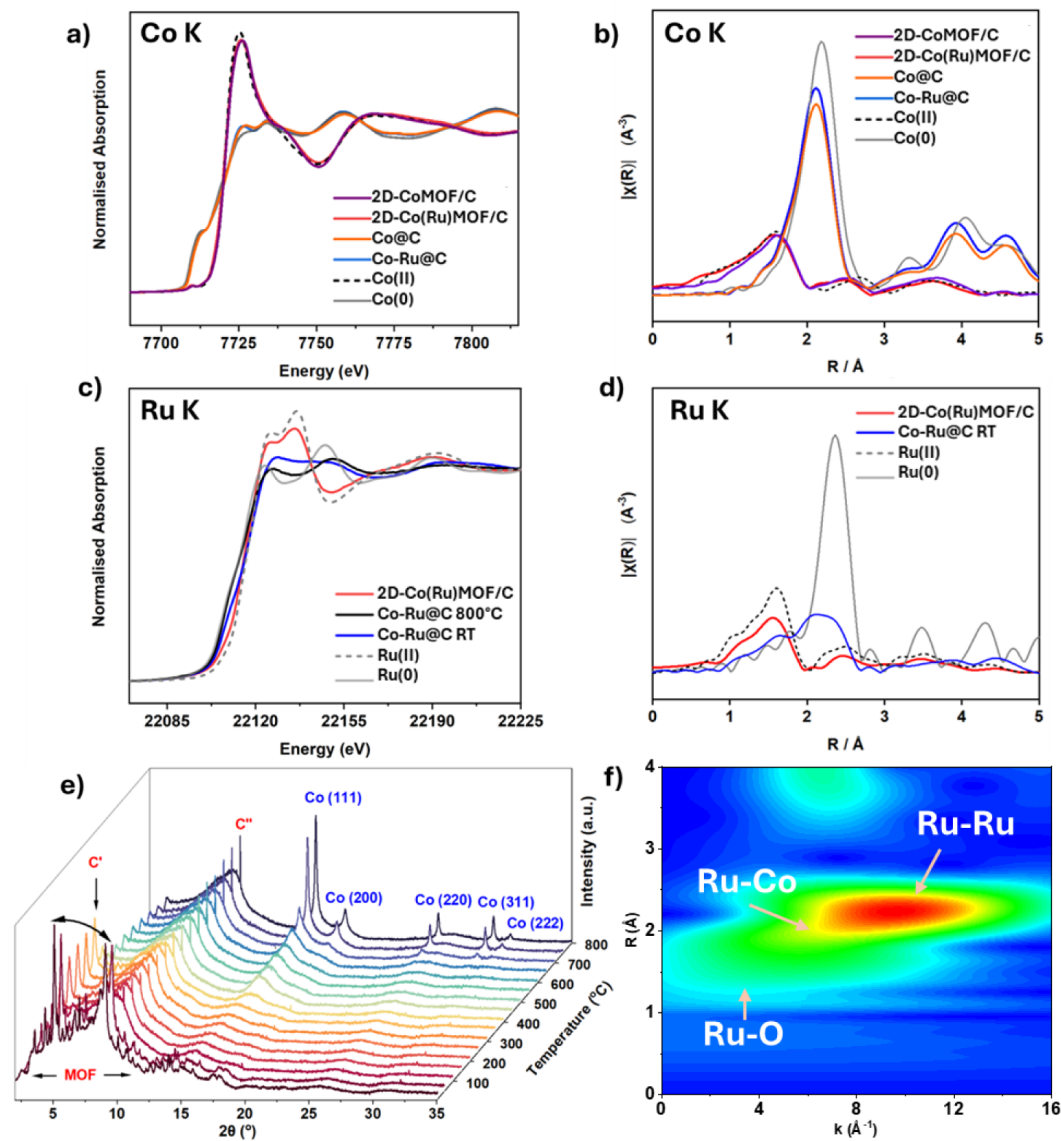
a) XANES spectra at the Co K edge for the monometallic and bimetallic systems before (2D-CoMOF/C and 2D-Co­(Ru)­MOF/C) and after pyrolysis (**Co@C** and **Co–Ru@C**). b) *k* ^2^ -weighted Fourier transform in *R*-space for monometallic and bimetallic systems before (2D-CoMOF/C and 2D-Co­(Ru)­MOF/C) and after pyrolysis (**Co@C** and **Co–Ru@C**). Data were collected *ex-situ* at the Co K-edge. c) XANES spectra at the Ru K edge comparing the bimetallic system before pyrolysis (2D-Co­(Ru)­MOF/C), after pyrolysis at 800 °C (**Co–Ru@C** 800 °C), and after cooling to room temperature (**Co–Ru@C** RT), all under N_2_. d) *k* ^2^ -weighted Fourier transform in *R*-space in bimetallic systems before (2D-Co­(Ru)­MOF/C) and after pyrolysis (**Co–Ru@C**). Data were collected *ex situ* at the Ru K-edge. Relevant reference spectra are included for each case, both the metal foils and either Co­(II) or Ru­(II) in Co­(OAc)_2_ and [Ru­(bpy)_3_]­Cl_2_, respectively. e) PXRD at 23 keV performed on the same 2D-Co­(Ru)­MOF/C sample during a temperature ramp to 800 °C in N_2_, mimicking the pyrolysis step in the synthesis of **Co–Ru@C**. f) The wavelet transform analysis for the Ru K-edge EXAFS spectra of **Co–Ru@C** after *in situ* pyrolysis, showing the individual contributions of different scattering paths.

The evolution of the metal precursors during pyrolysis was monitored using simultaneous *in situ* PXRD and XAS, providing real-time insights into both structural transformations and changes in metal speciation. This combined approach is essential for elucidating how MOF-derived catalysts transition into their active phases and for identifying the formation of alloys or intermediate species that may influence catalytic performance. Initial *ex-situ* XAS at the Co K-edge for the pyrolyzed catalysts, **Co@C** and **Co–Ru@C**, showed that Co (II) in the precursors is fully reduced to metallic Co(0), as evidenced by a XANES spectrum closely matching that of Co foil (Figure 2a). The corresponding EXAFS analysis further supports these findings, indicating a high degree of Co–Co coordination consistent with the formation, after pyrolysis, of relatively large metallic nanoparticles, similar to those found in bulk cobalt (Table S2 and Figure S6). Notably, no evidence of Co–Ru interactions was observed in the EXAFS spectra at the Co K-edge, suggesting that such interactions are either absent or fall below the detection limit from the perspective of Co as the absorbing atom. This is likely due to the high Co:Ru atomic ratio, which reduces the statistical likelihood of detecting Co–Ru scattering events. To further investigate the behavior of ruthenium during pyrolysis, in situ XAS measurements at the Ru K-edge (22.2 keV) were performed in parallel with PXRD at 23 keV.

*In situ* PXRD (Figure 2e) reveals the loss of the MOF’s crystalline order at temperatures close to 100 °C. However, complete decomposition does not occur until approximately 400 °C, as indicated by the TGA profiles shown in Figures S4 and S5. In the same temperature range, following the loss of the ordered MOF structure, an amorphous carbon contribution appears, centered at 8.5° 2θ, and remains constant throughout the entire thermal ramp. As discussed in a previous work,^33^ this signal corresponds to the (002) reflection of turbostratic carbon, characterized by rotationally random and disordered stacking of the layers due to the spherical nature of the carbon shell.^48^ This structural disorder accounts for the broadness of the reflection. Additionally, two more crystalline carbon phases are observed. The first, detected at 3.6° 2θ and labeled C′, corresponds to a graphene oxide phase,^49^ which forms concurrently and decomposes between 350 and 400 °C. The second, designated as C″, is attributed to graphite and is present throughout the heating process, with its scattering intensity increasing at higher temperatures. This phase displays a sharp and intense reflection at 9.1° 2θ, assigned to the (002) reflection of graphite.^48,49^ The appearance of *fcc* Co(0) reflections began around 700 °C and became sharper and more intense up to 800 °C, indicating crystallite growth. All phases remain stable during the 2 h hold at 800 °C (Figure S7), with an apparent slight increase in crystallite size and overall crystallinity upon cooling (Figure S8). Final Co nanoparticle sizes determined by PXRD were 15 nm for **Co@C** and 17 nm for **Co–Ru@C** (Table S3, Figures S9 and S10).

Regarding Ru, no PXRD-detectable crystalline phases were observed throughout the temperature ramp. However, in situ XAS data revealed a gradual reduction of Ru­(II) to Ru(0) beginning at ∼300–400 °C (Figure S11a). Linear combination fitting (LCF) of XANES spectra indicated that by 800 °C, approximately 90% of Ru was in the metallic state (Figure S11b), with no significant changes during the subsequent isothermal hold (Figure S12). Upon cooling, partial reoxidation of Ru(0) was observed (Figure 2c), a common phenomenon among platinum group metals (PGMs) attributed to redispersion into smaller oxidized particles.^50^ EXAFS data at the Ru K-edge for the cooled sample exhibit unexpected features for monometallic Ru nanoparticles. First, the partial oxidation observed in the XANES is reflected in the Fourier transform (FT) by a Ru–O contribution centered at approximately 1.9 Å. Additionally, in bulk hcp-structured Ru, the typical Ru–Ru metallic bond distance is around 2.68 Å.^51^ However, in this case, the FT shows a main peak shifted to shorter distances with an asymmetric distribution, which could be attributed to the presence of additional Ru–Co interactions. Wavelet transform (WT) analysis of the EXAFS spectra enables the resolution of individual scattering paths in both real space (*R*) and *k*-space simultaneously, producing a two-dimensional representation that combines spatial and energetic information. In this analysis, as shown in Figure 2f, the signal associated with single scattering from Ru–O path reaches maximum intensity at low *k*-values, which is characteristic of lighter elements.^52^ In contrast, the Ru–Ru signal extends to higher *k*-values, consistent with the stronger scattering from a heavier atom such as Ru. In addition, a third contribution is identified, distinct from the Ru–Ru signal, appearing at slightly lower *R* and *k* values. This feature can be tentatively assigned to a Ru–Co interaction, likely responsible for the asymmetry and the FT shift toward shorter distances. It does not correspond to the expected Fourier distance of a Ru–O path or a multiple-scattering Ru–O–Ru pathway (Figure S13). Moreover, its relatively high intensity, combined with the weak Ru–O signal observed in the pyrolyzed sample, further supports the hypothesis of a structural Ru–Co interaction.

Electron microscopy techniques were employed to investigate the morphology and metal dispersion on the carbon support of the catalysts after pyrolysis and to shed light on the nature of the Ru–Co interaction. High-resolution transmission electron microscopy (HRTEM) provided further insight into the crystalline structure of the metal nanoparticles. In **Co@C**, lattice fringes with *d*-spacings of 1.86 Å and 2.07 Å were observed, corresponding to the (200) and (111) planes of face-centered cubic (*fcc*) cobalt (JCPDS: 00-015-0806) (Figure 3a). Similarly, for **Co–Ru@C**, cobalt nanoparticles showed lattice fringes with *d*-spacings of 2.07 Å and 2.15 Å, also assignable to *fcc* cobalt (100) and (111) planes (Figure 3c). These results confirm that cobalt retains an *fcc* crystalline structure after pyrolysis and suggest that ruthenium does not significantly alter the crystallinity of the cobalt phase. Scanning transmission electron microscopy (STEM) of **Co@C** revealed well-dispersed spherical cobalt nanoparticles with an average size of 11 ± 5 nm (Figure 3b). In contrast, the bimetallic **Co–Ru@C** material displayed two distinct nanoparticle populations embedded in the carbon matrix (Figure 3d). Energy-dispersive X-ray spectroscopy (EDX) analysis indicated that the larger particles were cobalt-rich (19 ± 6 nm), while smaller, surrounding particles corresponded to ruthenium, with an average size of 2 ± 1 nm.

**Figure 3:**
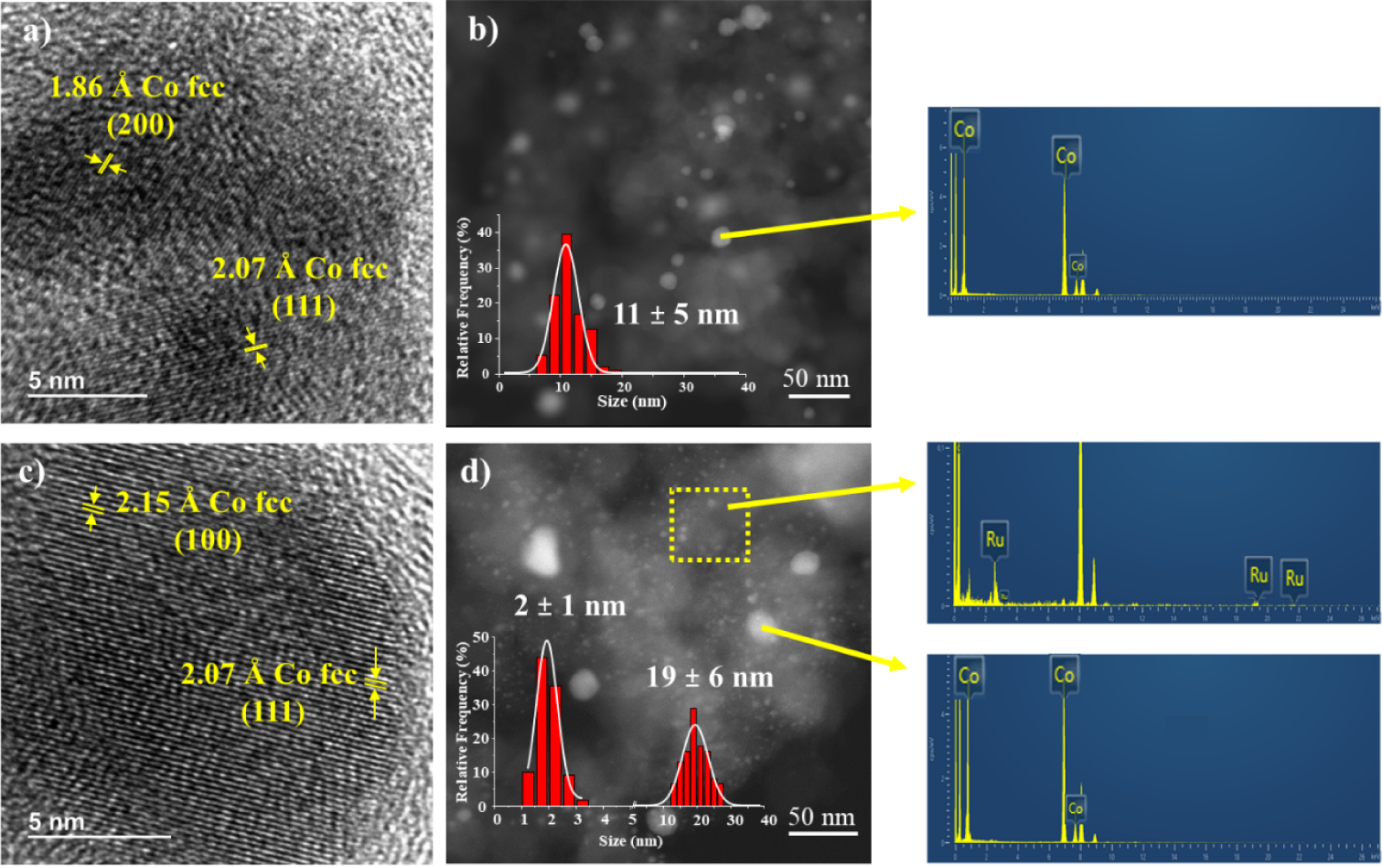
a) HRTEM micrograph of **Co@C**. Lattice spacings are highlighted in yellow, b) STEM-HAADF coupled with EDX analysis of **Co@C**, where the map spectrum is shown in the right part, c) HRTEM micrographs of cobalt nanoparticles of **Co–Ru@C**. Lattice spacings are highlighted in yellow, d) STEM-HAADF coupled with EDX analysis of **Co–Ru@C**, where the map spectrum is shown in the right part. EDX signals at 0.9, 8.1, and 8.9 keV correspond to the copper grid.

Overall, these structural and spectroscopic results indicate a significant interaction between Ru and Co in the **Co–Ru@C** catalyst after pyrolysis and cooling. Two possible scenarios may account for this structural behavior. The first is the formation of a Ru–Co alloy, where Ru atoms are incorporated into the Co *fcc* phase during pyrolysis (see Tables S4–S6 and Figure S14). This would be consistent with the shift of the main EXAFS peak to shorter distances than typical Ru–Ru bonds in an *hcp* structure, as well as the observed asymmetry in the distribution, suggesting a mixed coordination environment. However, while alloy formation cannot be ruled out, it is not clearly supported by the Co K-edge XAS data, where no Ru–Co interactions were detected, likely due to the low relative concentration of Ru. A more plausible explanation, considering the microscopy findings, is that the Ru–Co interaction arises from close contact between small Ru nanoparticles and larger Co nanoparticles within the carbon matrix. This hypothesis is supported by STEM–EDX, which shows spatial proximity between Ru- and Co-rich regions, as well as by the lack of significant changes in Co crystallinity, as shown by PXRD and HRTEM. In this case, the EXAFS signal at the Ru edge would reflect surface-level Ru–Co interactions at the nanoparticle interface, rather than bulk alloy formation.

### Optimization of Reaction Conditions

The one-pot reductive coupling of 1,2-dinitrobenzene **(1a)** with benzaldehyde **(2a)** to synthesize 2-phenylbenzimidazole **(4aa)** was chosen as the model reaction to study the doping effect of Ru on Co nanoparticles supported on carbon. It should be mentioned that our group has previously reported that cobalt-based catalysts performed very well in reductive amination reactions in water.^22^ So, the initial experiments were conducted under the best state-of-the-art conditions (150 °C and 30 bar H_2_) for the two catalysts **Co@C** and **Co–Ru@C** (Table 1) using water as solvent. While **Co@C** did not achieve full conversion and showed poor selectivity (entry 1, Table 1), **Co–Ru@C** achieved full conversion and selectivity to the product (entry 2, Table 1), prompting further study of the influence of temperature and pressure (entries 3–7, Table 1) on the reaction with this catalyst. Both parameters were adjusted in order to find the mildest reaction conditions, resulting in full conversion and selectivity to 2-phenylbenzimidazole at impressively low temperatures and pressures of 135 °C and 15 bar H_2_ (entry 5, Table 1). Under the same reaction conditions, **Co@C** only achieved 30% conversion and 4% selectivity (entry 8, Table 1), demonstrating that the presence of small amounts of noble metals like ruthenium plays a key role in the reaction.

**Table 1:**
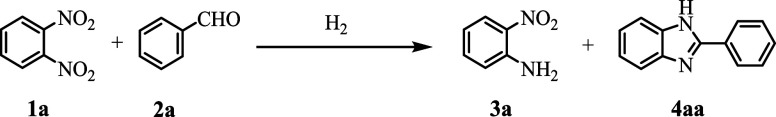
Optimization of the Reaction Conditions^a^

					**Conversion (%)**	**Selectivity (%)**	
**Entry**	**Catalyst**	***T*** (°C)	***P*** (bar)	**t (h)**	**1a**	**3a**	**4aa**
1^b^	Co@C	150	30	20	60	91	9
2^c^	Co–Ru@C	150	30	20	>99	<1	>99
3^c^	Co–Ru@C	150	20	20	>99	<1	>99
4^c^	Co–Ru@C	150	10	20	>99	26	74
5^c^	**Co–Ru@C**	**135**	**15**	**12**	**>99**	**<1**	**>99**
6^c^	Co–Ru@C	120	30	16	>99	<1	>99
7^c^	Co–Ru@C	120	20	20	82	82	18
8^b^	Co@C	135	15	12	30	96	4
9^d^	Ru@C	135	15	16	11	94	6
10^e^	Ru@C	135	15	16	33	96	4
11^f^	Ru@C + Co@C	135	15	16	47	93	7
12^f^	Ru@C^e^ + Co@C	135	15	16	51	94	6

a Reaction conditions: **1a** (1 mmol), **2a** (1.5 mmol), H_2_O (3 mL). Conversion and selectivity were determined by GC and GC-MS using benzonitrile as an external standard.

b 10 mg catalyst (1.8% mol Co).

c 10 mg catalyst (1.7% mol Co, 0.1% mol Ru).

d 3 mg of 2.5% Ru@C (0.1% mol Ru).

e 3 mg activated commercial 5% Ru@C (50% H_2_O content) (0.1% mol Ru).

f Physical mixture resulting 1.7% mol Co, 0.1% mol Ru.

To demonstrate that Ru is not solely the active center in the **Co–Ru@C** catalyst, and that both metals are necessary in order to carry out the reaction, two Ru-based catalysts were tested under the optimized reaction conditions. First, a **Ru@C** catalyst synthesized following the same synthetic route as for the **Co–Ru@C** was tested (entry 9, Table 1). Additionally, a commercial activated Ru supported on carbon was tested (entry 10, Table 1). These trials resulted in low conversion of 1,2-dinitrobenzene (11% and 33%, respectively) and very low selectivity to the target molecule **4aa** (6% and 4%, respectively), even with a longer reaction time than for the optimized conditions.

Furthermore, to prove that both metals need to interact and there is a synergistic effect between Co and Ru, two physical mixtures of our **Co@C** with commercial and prepared **Ru@C** were used as catalysts. As shown in entries 11 and 12 in Table 1, these tests resulted in 47% and 51% conversion and 7% and 6% selectivity to the product, respectively, which is approximately the sum of the activity of each catalyst separately.

### Study of Other Solvents

The effect of other solvents was also investigated as reaction media using **Co–Ru@C** (Table S7). In general, all employed, regardless of their polarity, were suitable for achieving complete conversion of the starting material in this reaction. However, the selectivity toward the desired molecule varies slightly depending on the solvent used. It is important to highlight that toluene solvent, which is the most apolar and nonprotic one (entry 1, Table S7), is the only one where the reaction mechanism is clearly altered, because the product distribution is almost evenly split between the target product and 1-benzyl-2-phenylbenzimidazole (product **5a)**, which is a reaction byproduct. Previous studies have already reported the formation of this byproduct when this solvent was used.^21,53^ In the case of THF, a polar aprotic solvent, the selectivity for the desired product is relatively high, although 8% of product **5a** is still present. This increase in selectivity can be attributed to the ability of THF to stabilize polar intermediates, such as the imine group (−C=NH) involved in the reaction mechanism.^22,54^ Additionally, it facilitates the dispersion of protonated species required in acid/base catalysis to activate the reactants, such as the protonation of nitro or carbonyl groups present in this reaction. Ethyl acetate achieves nearly complete selectivity toward the target molecule **4aa** (entry 3, Table S7) and it is the use of polar protic solvents that allows for both conversion and selectivity >99% (entries 4–6, Table S7).

The choice of water as a reaction medium has rested solely on its higher sustainability as a solvent. To obtain a better understanding of the influence of H_2_O in the reaction mechanism, various experiments were conducted employing D_2_O as the solvent and D_2_ as the reducing agent. A noticeable effect was detected as the D_2_O:H_2_O ratio increased, resulting in decreased selectivity toward the desired product **4aa** (Figure S15). These findings align with previous reports indicating that reactions proceed at slower rates in deuterated water compared to H_2_O.^55^ This phenomenon is mainly due to physical properties of D_2_O, such as higher viscosity and stronger hydrogen bonding,^56^ and to other factors, including kinetic isotope effects, lower dielectric constant and decreased ion mobility.^57^ On the other hand, when reactions are carried out using D_2_ instead of H_2_, isotopic effects known as Kinetic Isotope Effects (KIE) may occur again, primarily influencing reaction rates and product distributions.^58,59^ As shown in Figure S15, using D_2_ as a reductant leads to a slight decrease in both conversion and selectivity toward the target product compared to hydrogen. These findings corroborate previous investigations demonstrating that deuterium slows reaction rates relative to hydrogen when used as a reducing agent with water as the solvent.^60^ This phenomenon arises because the C–D bond is slightly stronger and has a lower vibrational energy than the C–H bond, given that the atomic mass of deuterium is twice that of hydrogen.

### Unveiling the Intricacies of the One-Pot Reaction

To further evaluate the role of each metal in the reaction pathway, UV–vis spectroscopy and H_2_-D_2_ isotopic exchange studies were performed. First, the adsorption of 1,2-nitroaniline (**3a**) and 2-phenylbenzimidazole (**4aa**) on **Co@C** and **Co–Ru@C** was investigated using UV–vis spectroscopy (Figures S16 and S17). Both catalysts showed similar adsorption of the intermediate and similar desorption of the final product (Figure S18), suggesting that Co is responsible for the adsorption of the organic molecules, and the difference between these two catalysts is likely related to the activation of molecular hydrogen. To shed some light on the role of ruthenium, the efficiencies of **Co@C** and **Co–Ru@C** in H/D exchange tests were measured in a flow reactor, and the H_2_, D_2_ and HD species were detected using an *in situ* mass spectrometer. These isotopic experiments showed that **Co–Ru@C** dissociates H_2_ faster than **Co@C** with HD/H_2_ mass signal ratios of 0.360 and 0.047, respectively (Figure S19). The higher rate of HD formation with **Co–Ru@C** is related to its higher hydrogen cleavage efficiency. Thus, up to this point, the key difference between these two catalysts appears to lie in their ability to dissociate the molecular hydrogen.

These findings suggest that cobalt is responsible for the organic reactants’ adsorption, while ruthenium is more related to the hydrogen cleavage. Therefore, the presence of both metals is necessary to have the synergistic effect that makes **Co–Ru@C** so active under mild reaction conditions. However, as demonstrated by the results of the physical mixture, their close proximity is crucial because it optimizes the colocation of the cleaved hydrogen with the target organic molecule, thereby enhancing the desired reaction.

The straightforward synthesis of benzimidazoles through the reductive coupling of 1,2-dinitroarenes with aldehydes is a multistep catalytic reaction. To gain a more comprehensive understanding of the catalytic behavior of **Co–Ru@C**, the product profile distribution was monitored under the optimized reaction conditions. The reaction profile is typical for a cascade reaction. The reaction begins when the hydrogenation of 1,2-dinitrobenzene (**1a**) takes place, leading to the formation of 1,2-nitroaniline (**3a**) with some traces of 2-phenylbenzimidazole (**4aa**). After 4 h of reaction, the concentration of 1,2-nitroaniline reaches its maximum and then begins to decline as the product concentration increases. It is important to note that after 8 h of reaction, the reactant is fully converted and after 12 h, full selectivity to the desired product is achieved (Figure 4a).

**Figure 4:**
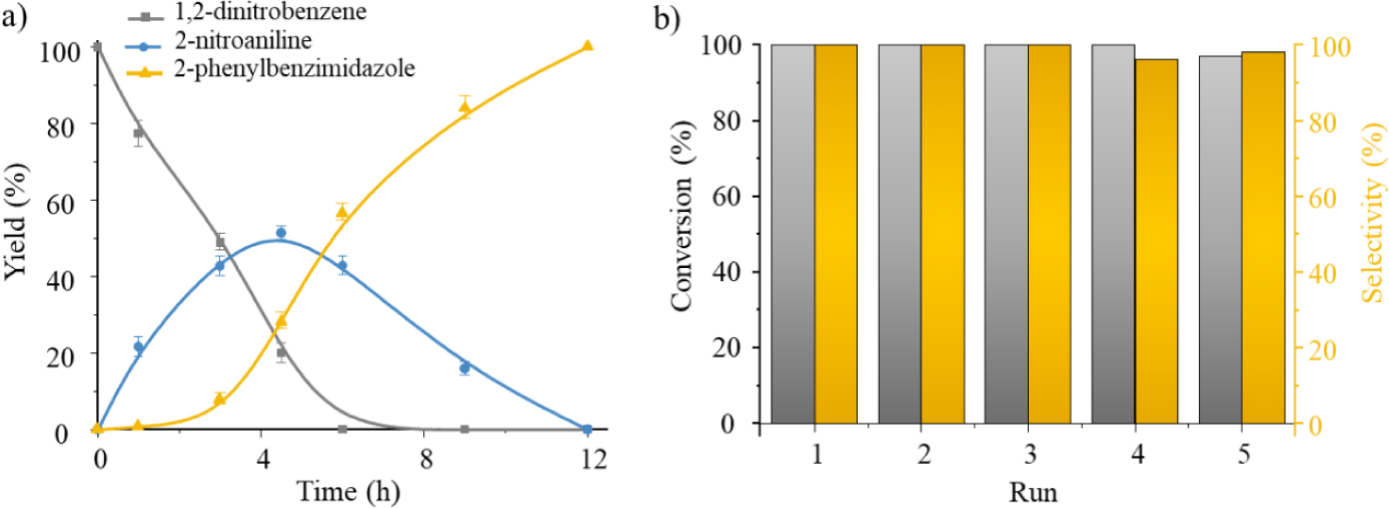
a) Product profile distribution of the synthesis of 2-phenylbenzimidazole (**4aa**) using **Co–Ru@C** as catalyst. b) Conversion of 1,2-dinitrobenzene **1a** (gray) and selectivity to 2-phenylbenzimidazole **4aa** (orange) for five reaction runs using **Co–Ru@C** as catalyst. Reaction conditions: **1a** (1 mmol), **2a** (1.5 mmol), H_2_O (3 mL), 135 °C, 15 bar H_2_, 12 h.

The obtained product distribution with this catalyst can also be explained through the reaction mechanism. This, using hydrogen as the reducing agent, has been studied previously for cobalt catalysts.^22,54^ In the case of Co_2_PN_*x*_@C catalyst in water, there is a full selectivity toward the 2-phenylbenzimidazole molecule, as for this **Co–Ru@C** catalyst. No byproducts appear in either case, as is the case of using {Mo_3_S_4_}_*n*_,^21^ which, for the same one-pot reaction, yields a selectivity value of 8% toward the 1-phenyl-2-benzimidazole byproduct.

### Stability and Heterogeneity of the Catalyst

For heterogeneous catalysts, it is crucial to ensure consistent performance over multiple uses, thereby reducing the need for frequent replacement, minimizing operational costs, extending their lifespan and lowering resource consumption and process interruptions. After 5 cycles under identical optimized conditions to those of the first run, conversion and selectivity remain close to 100% (Figure 4b). Furthermore, ICP analyses of the reaction crudes after each cycle confirm the system’s heterogeneity, without evidence of metal leaching (Table S8). In addition, recycling experiments at incomplete conversion (ca. 50%) have been carried out to reveal that there is no loss in catalytic activity with both conversion and selectivity remaining constant throughout the cycles (Figure S20).

The analysis of the catalyst after 5 reaction cycles *via* HAADF-STEM and EDX shows that a good dispersion of the cobalt and ruthenium nanoparticles is maintained, with no apparent signs of sintering or the appearance of agglomerates. Thus, the histogram shows a distribution of 18 ± 5 nm for the Co nanoparticles and 2 ± 1 nm for the Ru nanoparticles, similar to the fresh material (*vide supra*). Moreover, the existence of cobalt nanoparticles surrounded by small ruthenium nanoparticles is still observed (Figure 5). The PXRD analysis also shows the preservation of the three characteristic peaks of the Co phase, corresponding to face-centered cubic (*fcc*) Co^0^ nanoparticles, which can be observed at 44°, 51°, and 76° (JCPDS: 00-015-0806).^61^ The absence of additional Ru-related reflections in the postreaction XRD pattern indicates the structural stability of the Ru nanoparticles, with no detectable changes in phase composition or particle size compared to the fresh catalyst (Figure S21).^62,63^

**Figure 5:**
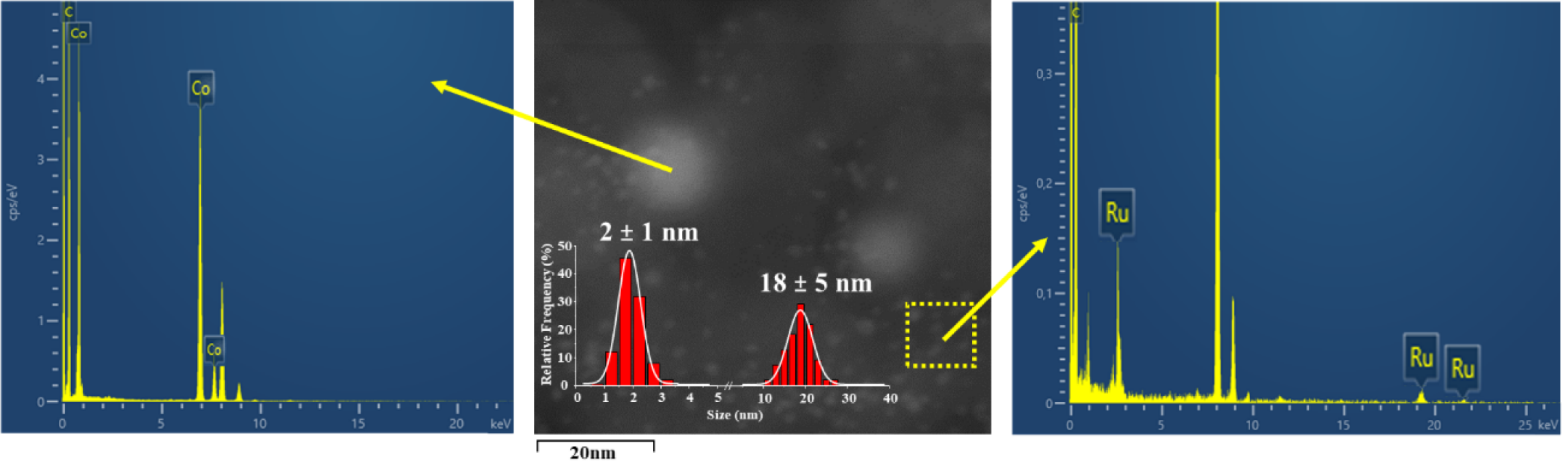
STEM image of **Co–Ru@C** after five runs (center panel). EDX spectrum of dispersed Ru nanoparticles marked with a yellow arrow (left panel). EDX spectrum of Co nanoparticles marked with a yellow arrow (right panel). EDX signals at 0.9, 8.1, and 8.9 keV correspond to the copper grid.

However, XAS indicates that, while the state of the Co is unaffected by reaction, the Ru undergoes some further oxidation (Figure S22). XANES of the pyrolyzed **CoRu@C** best fit to standards of Ru(0) and an Ru­(II) complex, discussed previously, and the spectra could be well fit with a linear combination of the two (74% and 26% respectively) (Figures S23,24 and Table S9). After catalysis, a third component, RuO_2_, is required to achieve a good fit of the spectra, with a composition of 47% Ru(0), 20% Ru­(II) and 33% RuO_2_. Therefore, while the Co–Ru interaction is preserved, demonstrated by the retained but diminished contribution of both Ru–Co and Ru–Ru paths in the Fourier transform, some metallic Ru is lost and new oxide nanoparticles are formed.

### Substrate Scope

With the optimal reaction conditions in hand (135 °C and 15 bar H_2_), the general scope of the developed catalyst for the reductive coupling of variously substituted 1,2-dinitrobenzenes and benzaldehydes was examined. As shown in Scheme 2, several 1,2-dinitroarenes with electron-donating or -withdrawing groups produced the corresponding substituted 2-phenylbenzimidazole with excellent yields. It can be seen that alkyl-substituted dinitroarenes exhibit very good extracted yields **(4ba–4ca)**. For halogen-substituted 1,2-dinitroarenes, there were no dehalogenation byproducts and the yield was not influenced by the halogen position (**4da, 4ea**). In this sense, introducing a second halogen atom does not decrease the reaction performance, obtaining high yields (**4fa, 4ga**). Furthermore, electron-rich dinitroarenes containing methoxy groups or cyclic acetal also underwent this reaction with very reasonable yields (**4ha, 4ia**).

**Scheme 2:**
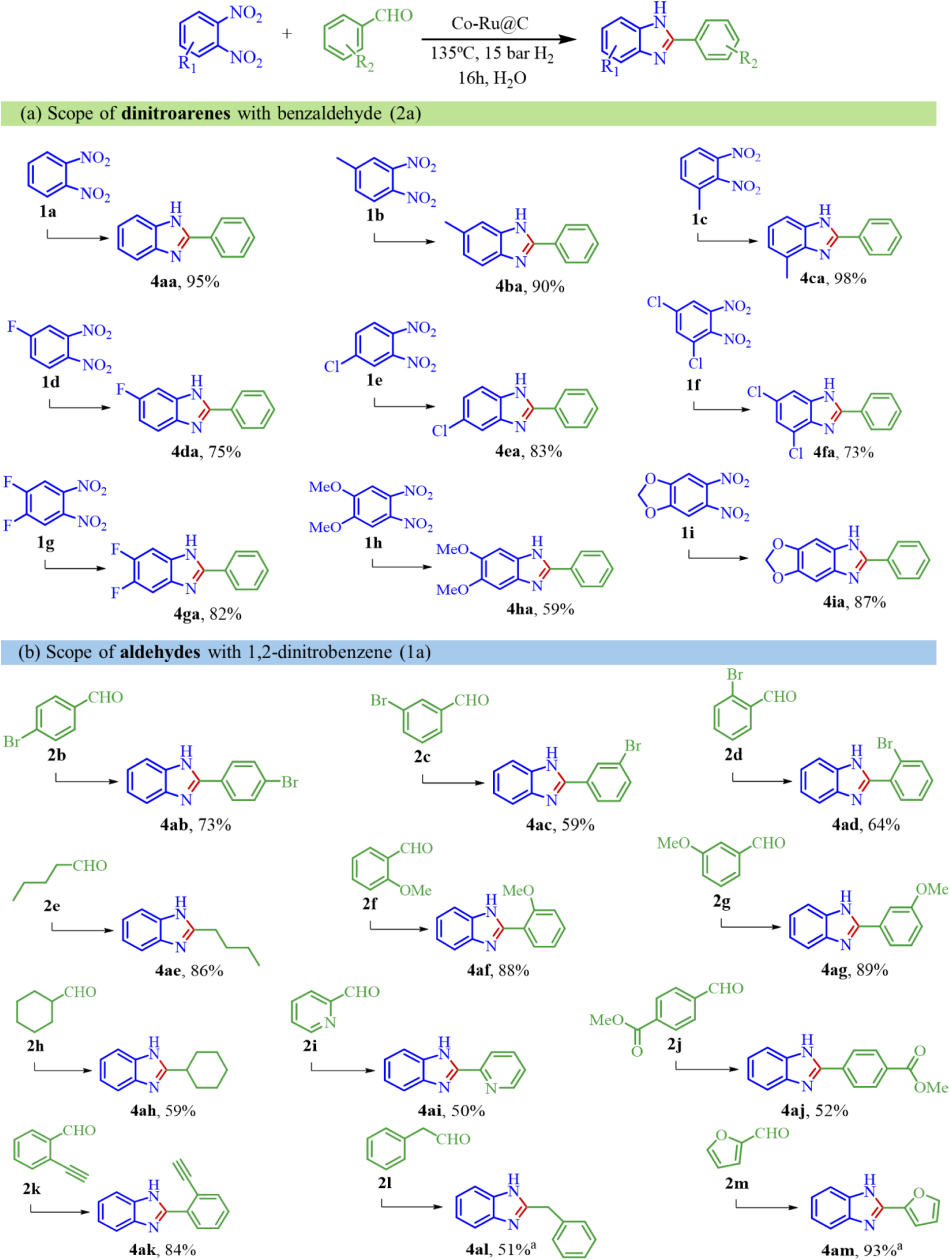
Catalytic Reductive Coupling of 1,2-Substituted Dinitroarenes and Substituted Aldehydes with **Co–Ru@C** [Fn sch2-fn1]

i Reaction conditions: **1a**–**1g** (0.5 mmol), **2a**–**2m** (0.75 mmol), **Co–Ru@C** (5 mg), H_2_O (3 mL), 15 bar H_2_, 135 °C, 16 h. Yields of isolated products are given. ^a^Scaled reactions: **1a** (2.5 mmol), **2l**–**2m** (3.75 mmol), **Co–Ru@C** (25 mg), H_2_O (15 mL), 15 bar H_2_, 135 °C, 16 h.

Scheme 2 also shows the applicability of various aldehyde groups in this hydrogenative reductive coupling reaction. The position of the halogen-substituted group showed a slight electronic and steric hindrance on the catalyst activity. For example, the 2-phenylbenzimidazole molecule **(4ac)** derived from 3-bromobenzaldehyde displayed the lowest extracted yield due to an electronically unfavorable effect. In contrast, the 4-bromobenzaldehyde derivative **(4ab)** achieved the highest isolated yield among the three, as it does not experience steric hindrance, unlike the 2-bromobenzaldehyde derivative **(4ad).** No debrominated compounds have been detected as side products, regardless of the bromine position in the benzaldehyde. Alkoxy and linear aldehydes were also evaluated, resulting in an excellent extracted yield of almost 90% (**4ae–4ag**). **Co–Ru@C** also exhibits acceptable yields when 1,2-dinitrobenzene is coupled with a cyclic aliphatic (**4ah**) and pyridine (**4ai**) group. The excellent selectivity of this catalytic system was investigated by applying substrates with sensitive groups, such as C≡C and RCOOR (**4aj–4ak**), obtaining their derivatives 2-phenylbenzimidazole with high yields. It is important to point out that no other byproducts than the corresponding reaction intermediate in each case have been found in any of the reactions, achieving always a full conversion of the starting material.

The high relevance of several benzimidazoles leads to the necessity of performing some proof of concept in order to investigate the scalability of the process. From this perspective, two half-gram-scale reactions were carried out to determine the quantity of highly valuable substituted 2-phenylbenzimidazole that can be obtained. Phenylacetaldehyde is used in industry mainly as a fragrance. Its coupling with 1,2-dinitrobenzene results in Diabazole (**4al**). This supplement is often marketed for its potential benefits in managing diabetes, apart from helping regulate blood sugar levels and its use as an antioxidant.^64^ On the other hand, biomass-derived furfural has a huge importance as a renewable resource because of its industrial application and environmental benefits. As a result of its coupling, a pesticide-denominated Fuberidazole (**4am**) is synthesized. It is primarily used to control fungal diseases in crops and inhibit the growth of fungi on vegetables and fruits.^65^ Both extracted yields demonstrate that the **Co–Ru@C** catalyst shows promising potential for industrial applications.

### Green Metrics

The primary goal of Green Chemistry is to reduce waste generation and eliminate the use of hazardous solvents and reagents.^66^ In this context, the one-pot synthesis of substituted 2-phenylbenzimidazoles shows a substantial improvement in process sustainability, as it significantly lowers both the E-factor and Process Mass Intensity (PMI) compared to conventional multistep approaches. This study introduces a multifunctional **Co–Ru@C** catalyst capable of converting 1,2-dinitrobenzene directly into 2-phenylbenzimidazole in a single step. In contrast, the traditional method, which involves the sequential hydrogenation of 1,2-dinitrobenzene to 1,2-nitroaniline followed by the reductive coupling with benzaldehyde, leads to higher E-factor and PMI values, in opposition to the principles of Green Chemistry. Moreover, organic synthesis frequently depends on conventional organic solvents that, while enabling high yields, do not align with the principles of sustainable chemistry. Although the use of green solvents should be prioritized, their application is often limited by lower selectivity. However, in this study, we demonstrate that sustainable solvents such as water and ethanol provide complete selectivity toward the target molecule. The selection of water as a reaction solvent is based on its superior sustainability compared to ethanol. Furthermore, water is a nonflammable, nontoxic and nonvolatile solvent, which significantly reduces safety risks in both laboratory and industrial settings. As the most abundant solvent in nature and readily accessible, it also offers a considerably lower cost. For this reason, we have conducted a study of the typical parameters used in Green Chemistry and Sustainability to show the greenness of our chemical process using **Co–Ru@C** as a catalyst (Table S10).^67^ Outstandingly, the E-factor (total mass of waste/mass of final product) yields a value of 16.2, which represents an extremely low result and is highly favorable for the sustainability of the process. The typical range for pharmaceutical chemistry, where the group of benzimidazoles is usually included, is *E* = 25–100, but this value could also be categorized as exceptional within the fine chemistry range (*E* = 5–50). The EcoScale tool has also been used to take into consideration factors such as the cost of reagents, process safety, reaction conditions and workup and purification. This tool considers a process to be more sustainable when the result is close to 100 points.^68^ In this case, the result is 90 points, which demonstrates the good sustainability of this process using this **Co–Ru@C** catalyst (Table S11). To conclude, the GreenStar tool is used to evaluate how many of the principles of sustainable chemistry are addressed in this chemical reaction. A higher area value indicates that a higher degree of greenness is fulfilled. In our case, the Green Star Area Index (GSAI) is 12.64, a moderate value that suggests the efficiency of the reaction, the use of reagents and the minimization of waste are balanced (Figure S25).^69^ Additional details on the calculation methodology are available in the Supporting Information.

## Experimental Section

### Catalyst Preparation

#### Preparation of **Co@C** Nanoparticles

**Co@C** nanoparticles have been synthesized following a two-step procedure. In the first step, one equivalent of [Co_4_O_4_(OAc)_4_(py)_4_] (0.46 mmol) and 4 equiv of 2,2′-bipyridine-4,4′-dicarboxylic acid (bda) (1.84 mmol) were dissolved in 10 mL of pyridine. Then, carbon powder (VULCAN XC72R, 100 mg) was added to the solution and it was mixed with 8 equiv of trifluoroacetic acid (TFA). The resulting solution was introduced into a stainless-steel autoclave, being heated at 150 °C for 9 days under autogenous pressure and dynamic conditions. Once cooled to room temperature, the solution was filtered and the powder was washed with acetone to remove the remaining pyridine solvent molecules, obtaining the precursor 2D-CoMOF/C. The obtained solid was dried under vacuum, after which it was ground to a fine powder. The material was transferred into a quartz reactor in the second step and placed in a vertical oven. The sample was pyrolyzed with a ramp rate of 25 °C/min and held at 800 °C for 2 h under nitrogen flow. Finally, the sample was cooled to room temperature under a nitrogen stream. The metal content of **Co@C** by FRX measurement revealed 11.1 wt % Co. Elemental analysis: 1.08% N, 82.48% C, 0.22% H, 0.38% S.

#### Preparation of **Co–Ru@C** Nanoparticles

**Co–Ru@C** nanoparticles have been synthesized following the same procedure, including the ruthenium salt in the first one-pot step. One equivalent of [Co_4_O_4_(OAc)_4_(py)_4_] (0.46 mmol) and 4 equiv of 2,2′-bipyridine-4,4′-dicarboxylic acid (1.84 mmol) were dissolved in 5 mL of pyridine. Moreover, 10.73 mg of RuCl_3_·*x*H_2_O were dissolved in 5 mL of ethanol. Both solutions were mixed, and carbon powder (VULCAN XC72R, 100 mg) and 8 equiv of trifluoroacetic acid (TFA) were added to the resultant solution. This mixture was introduced into a stainless-steel autoclave and heated at 150 °C for 9 days under autogenous pressure and dynamic conditions. The solution was filtered, washed with acetone and ground to a fine powder, obtaining the precursor 2D-Co­(Ru)­MOF/C. Finally, the material was pyrolyzed following the same procedure used for the **Co@C**. The metal content of **Co–Ru@C** by FRX measurement revealed 9.6 wt % Co and 0.8 wt % Ru. Elemental analysis: 1.03% N, 78.64% C, 0.25% H, 0.29% S.

#### Preparation of **Ru@C** Nanoparticles

**Ru@C** nanoparticles have been synthesized following the same procedure without incorporating the cobalt precursor. In this case, 4 equiv of 2,2′-bipyridine-4,4′-dicarboxylic acid (1.84 mmol) were dissolved in 5 mL of pyridine. Moreover, 10.73 mg of RuCl_3_·*x*H_2_O were dissolved in 5 mL of ethanol. Both solutions were mixed, and carbon powder (VULCAN XC72R, 100 mg) and 8 equiv of trifluoroacetic acid (TFA) were added to the resultant solution. This mixture was introduced into a stainless-steel autoclave and heated at 150 °C for 9 days under autogenous pressure and dynamic conditions. The solution was filtered, washed with acetone and ground to a fine powder. Finally, the material was pyrolyzed following the same procedure used for the **Co@C**. The metal content of **Ru@C** by FRX measurement revealed 2.4 wt % Ru. Elemental analysis: 0.65% N, 90.74% C, 0.35% H, 0.39% S.

### Experimental Catalytic Evaluation

#### General Procedure

Catalytic tests were conducted in a 12 mL stainless-steel batch reactor. The reactor contained 10 mg of catalyst, 1,2-dinitrobenzene (168.2 mg, 1 mmol), benzaldehyde (150 μL, 1.5 mmol) and 3 mL of water. Then, the reactor was purged with 15 bar of H_2_ three times. Finally, the pressure was set to 15 bar H_2_ and the temperature to 135 °C. Once finished, the reaction crude is mixed with 6 mL of THF, generating a homogeneous mixture. Catalytic performance was followed by gas chromatography using benzonitrile (100 μL, 0.97 mmol) as external standard.

#### Substrate Scope

For the substrate scope, the same procedure was followed, using 0.5 mmol of the corresponding dinitrobenzene, 0.75 mmol of the substituted aldehydes and 5 mg of catalyst. Upon completion of the reaction, the resulting crude mixture underwent purification via silica gel column chromatography. The column was prepared by packing it with silica gel and pre-equilibrating it with the eluent. The crude product, preadsorbed onto silica gel, was loaded onto the column and elution was carried out using a gradient solvent system comprising hexane and ethyl acetate. Fractions containing the purified product were identified through thin-layer chromatography (TLC), pooled together, and concentrated under reduced pressure to afford the final compound.

#### Scale-Up Reaction

The scale-up reaction to synthesize Diabazadole and Fuberidazole was carried out in a 25 mL engineer stainless-steel batch reactor. The substrate scope protocol was followed with the difference that the mass of all reaction components was multiplied by a factor of 5. In more detail, 25 mg of catalyst, 2.5 mmol of 1,2-dinitrobenzene (420.5 mg), 3.75 mmol of the substituted benzaldehyde (310 μL in case of furfural and 415 μL in case of phenylacetaldehyde) and 15 mL of water were added to the reactor. Then, the reactor was purged with 15 bar of H_2_ three times. Finally, pressure and temperature were set to 15 bar H_2_ and 135 °C.

#### Calculation of Conversions and Selectivities

In all cases, the calibration was done using benzonitrile as an external standard (ES). The response factor of the analytes (RF_i_) was determined by injecting known quantities of each analyte *i* into the corresponding GC.

The amount of each of the reactants and products during the reaction is therefore calculated as follows:

$$n_{i}=\frac{A_{i}\cdot n_{ES}}{A_{ES}\cdot RF_{i}}$$

being *A_i_* the area of the analyte, *A*_ES_ the area of the external standard (benzonitrile) and *n*_ES_ the exact mol of the standard added.

Thus, the conversion of reactant 1,2-dinitrobenzene (1,2-DNB) is calculated as follows:

$${\mathrm{conversion}}_{i}\left(t\right)\;\left(\%\right)=\frac{n_{i}\left(t=0\right)-n_{i}\left(t\right)}{n_{i}\left(t=0\right)}\times 100$$

The selectivity of each product is calculated as follows:

$${\mathrm{selectivity}}_{i}\left(t\right)\;\left(\%\right)=\frac{n_{\mathrm{product}\;i}\left(t\right)}{\sum n_{\mathrm{products}}\left(t\right)}\times 100$$

### Stability of the Catalyst

The stability of **Co–Ru@C** catalyst was demonstrated through a reusability test in water. After 16 h of reaction time, the spent catalyst was filtered and washed with ethyl acetate. The catalyst was pyrolyzed at 200 °C for 2 h under a nitrogen flow of 20 mL/min to remove some residual deposits on the catalyst surface, which cannot be easily eliminated otherwise. Then, the catalyst is placed another time in a clean reactor and the next run proceeds under general reaction conditions. Another four runs were executed in water with significant maintenance of conversion and selectivity throughout. ICP analyses revealed no loss of metallic cobalt and ruthenium charge over multiple uses. For the experiments carried out at approximately 50% conversion, the previous procedure was replicated, except that the reaction time was set to 3 h.

## Conclusions

This work introduces a new methodology for preparing a cobalt-based heterogeneous catalyst doped with a small amount of ruthenium, markedly enhancing catalytic activity in valuable organic transformations. The **Co–Ru@C** catalyst is a novel material in which small ruthenium nanoparticles (0.8 wt %) are integrated with larger cobalt nanoparticles (9.6 wt %). Its precursor is obtained in a one-pot process that generates a well-defined cobalt metal–organic network doped with ruthenium and supported on Vulcan carbon, 2D-Co­(Ru)­MOF/C. Subsequent pyrolysis at 800 °C under N_2_ results in **Co–Ru@C**, and *in situ* PXRD and EXAFS reveal intimate contact between Co and Ru nanoparticles and show that both metals are predominantly in the metallic state, features crucial for the target reaction. Reductive amination of dinitrobenzenes to benzimidazoles was selected as a model transformation, using molecular hydrogen as the reductant and water as the solvent, conditions fully aligned with green-chemistry principles. The **Co–Ru@C** synergy enables quantitative product yields under markedly milder pressure, temperature and reaction times than state-of-the-art protocols. H/D exchange experiments confirm that Ru accelerates H_2_ dissociation and hence the overall kinetics, whereas undoped **Co@C** cannot improve the benchmark conditions.

The catalyst can be reused at least five times and postcatalysis characterization (HAADF-STEM, HRTEM, XEDS and PXRD analyses) shows no structural degradation or metal leaching. In addition, this catalyst is active for the obtention of more than 20 functionalized benzimidazoles in high yields by using different dinitrobenzenes and benzaldehydes, including halogen-, alkyne- and ester-substituted compounds. Half-gram-scale syntheses of the bioactive and commercial compounds Diabazole and Fuberidazole further demonstrate the scalability of the reaction. The use of water, hydrogen and full conversion and selectivity translates into outstanding green-chemistry metrics. Overall, **Co–Ru@C** offers a versatile platform for designing new metal-doped materials for the sustainable synthesis of value-added organic molecules.

## Supplementary Material



## Notes

Additional characterization of the catalysts (PXRD, SEM, EXAFS, TGA, XANES, UV–vis TEM), study of other solvents, ^1^H NMR and ^13^C NMR data of the isolated products (PDF)
