# Supplementary material for: Green One-Pot Synthesis of Benzimidazoles from Dinitroarenes in Water Using a Ru-Doped Co-Based Heterogeneous Catalyst
Source: ACS Sustain Chem Eng. 2025 Sep 8;13(37):15533–46. doi: 10.1021/acssuschemeng.5c05806 (PMC12458975; doi:10.1021/acssuschemeng.5c05806)
Supplement: Supplementary file 1 [file sc5c05806_si_001.pdf]

Supporting Information

Green One-Pot Synthesis of Benzimidazoles from Dinitroarenes in Water Using a Ru-doped Co-Based Heterogeneous Catalyst

Jose Luis del Río-Rodríguez,[a] Silvia Gutiérrez-Tarriño,[a,*] María Asunción Molina,[b,c] Lucy Costley-Wood,[b,c] Chiara Falcini,[a,d] Andrew M. Beale,[b,c] Pascual Oña-Burgos.[a,*]

[a]*Instituto de Tecnología Química, Universitat Politècnica de València-Consejo Superior de Investigaciones Científicas (UPV-CSIC), Avda. de los Naranjos s/n, 46022 Valencia, Spain.*

[b]*Department of Chemistry, University College London, 20 Gordon Street, WC1H 0AJ, UK*

[c]*Research Complex at Harwell, Rutherford Appleton Laboratories, Harwell Science and Innovation Campus, Harwell, Didcot, OX11 0FA, UK*

[d]*Departamento de Química Orgánica, Universidad de Sevilla, c/Profesor García González 1, 41012 Sevilla, Spain*

* Corresponding authors

Silvia Gutiérrez-Tarriño silgutar@itq.upv.es

Pascual Oña-Burgos pasoabur@itq.upv.es

Number of pages: 52

Number of figures: 25

Number of tables: 11

Table of contents

1. General Information 3

2. Characterization of the precursors 5

3. Catalysts characterization...8

4. Study of other solvents...16

5. Unravelling the active species..17

6. Catalyst stability...19

7. Green Metrics calculations...23

8. Characterization data and experimental details of the isolated benzimidazoles......25

9. References 52

1. General Information

All reagents and solvents were purchased from commercial suppliers and used without further purification.

Nuclear magnetic resonance (NMR). ^{1}H and ^{13}C NMR were recorded on a Bruker 300 spectrometer and the chemical shifts are reported in ppm relative to residual proton solvent signals. Data for ^{1}H NMR spectra are reported as follows: chemical shift (δ, ppm), multiplicity (s = singlet, d = doublet, t = triplet, q = quartet, m = multiplet, dd = double doublets, bs = broad signal), coupling constant and integration. Data for ^{13}C NMR spectra are reported in chemical shift (δ, ppm).

High-Resolution TEM (HRTEM). HRTEM images of **Co@C** and **Co-Ru@C** before and after catalysis were obtained after a drop of the corresponding material was suspended on a copper grid. HRTEM images were acquired at the "Servicio de Microscopía Electrónica" of Universitat Politècnica de València (UPV) using a JEOL 2100F microscope operated at 200 kV in both the transmission (TEM) and scanning transmission (STEM) modes. ImageJ software was used to estimate individual nanoparticle sizes. The analysis of HR-TEM images to identify interplanar distances was done with Gatan Digital Micrograph software. The identification of the crystalline phases was performed by comparing the obtained interplanar distances with those provided by the Software X̀ Pert HighScore Plus database.

X-ray Flourescence (XRF). X-ray fluorescence spectra of the catalysts were recorded in a Zetium XRF spectrometer. Before measuring the catalysts, the calibration curve is adjusted to the predicted concentration of the analyte using commercial standards.

Gas Chromatography. GC analyses were acquired on an Agilent gas chromatograph equipped with an HP5 capillary column (30m x 250 μm). The instrument was set to an injection volume of 1μL, an inlet split ratio of 50:1, and inlet and detector temperatures of 280 ºC and 300 ºC, respectively. Nitrogen was used as carrier gas with a constant pressure of 10 psi. The temperature program used for all the analyses is 80.0 ºC, 1 min; 10 ºC/min to 160 ºC, 14ºC/min to 280, 15 min.

GC/MS. GC/MS analyses were acquired on an Agilent 6890 Network gas chromatograph coupled to an Agilent 5973 Network mass selective detector equipped with an Agilent HP5-M5 capillary column (30m x 250 μm). The instrument was set to an injection volume of 1μL, an inlet split ratio of 60:1, and inlet and detector temperatures of 250 ºC and 280 ºC, respectively. Helium was used as carrier gas with a constant flow rate of 1.2 mL/min. The temperature program used for all the analyses is as follows: 50.0 ºC, 2 min; 30 ºC/min to 280ºC, 15 min.

UV-Vis Absorption. A Cary-50 spectrophotometer was employed to record the UV/Vis absorption spectra of the filtrates. All the spectra were recorded at room temperature, using quartz cuvettes of 1 cm optical path length and 3 mL of capacity.

Chemical analysis. Elemental analysis of nitrogen, carbon and hydrogen (N, C, H) contents of isolated solid materials were determined with a Carlo Erba 1106 elemental analyzer using sulfanilamide as reference. In addition, Varian 715-ES inductively coupled plasma atomic emission spectrometer (ICP-AES) was used in order to determine the content of leached Co. Prior to the measurement process, the crude reaction is filtered and disaggregated using H_2SO_4 98% and some drops of hydrogen peroxide at 100ºC. In all cases, the calibration curve is adjusted to the predicted concentration of the analyte using commercial standards.

Powder X-Ray Diffraction (XRD). X-ray diffraction (XRD)was used to determine the atomic periodical structure of the solids. The X-ray diffraction measurements were acquired according to the powder method, in Bragg−Brentano geometry, using a CUBIX diffractometer from PANalyticaloperating at 40 kV and 35 mA, and equipped with a PANalytical X'Celerator detector. X-ray radiation with from Cu Kα source was used in the range of 2° to 90° (2θ) with a step of 0.020° (2θ).

Thermogravimetric analysis (TGA). It was carried out in a Mettler Toledo TGA/SDTA 851 apparatus; using a heating rate of 10 ºC/min in a nitrogen stream until a temperature of 800 ºC was reached.

X-ray Absorption Spectroscopy (XAS) and X-ray Diffraction (XRD). The majority of the *ex-situ* XAS measurements were conducted at the ALBA Synchrotron (Cerdanyola del Vallès, Barcelona, Spain) on the BL16-NOTOS beamline (proposal 2023097780). Data were collected at the Co K-edge (7708 eV) and Ru K-edge (22117 eV) using either fluorescence or transmission mode, with a minimum of three spectra averaged for each sample. The samples (powders) were mixed with boron nitride and pressed into circular pellets. *Ex-situ* powder diffraction was also performed on the BL16-NOTOS beamline (proposal 2023097780) at 23 keV (0.5393 Å). Samples were loaded into 1.5 mm ID capillaries, some with varying wall thicknesses, resulting in different contributions of quartz to the diffraction patterns. One sample, **Co-Ru@C** post reaction, was measured on the B18 beamline at Diamond Light Source (rapid access proposal SP42561-1), with the Ru K edge measurements in fluorescence mode and the Co K edge measurements in transmission mode. Again samples were measured as pressed pellets, diluted here with cellulose, and Co and Ru foils were measured in order to calibrate these spectra with those measured at ALBA Synchrotron.

For the *in-situ* experiments, performed at ALBA synchrotron, samples were placed in a custom-designed capillary system, created and built by ALBA. The 2D-CoMOF/C or 2D-Co(Ru)MOF/C samples were inserted into a quartz capillary, fixed between two plugs of quartz wool. Nitrogen gas was flowed through the capillary at 20 mL/min, while the temperature was raised at 25 °C/min to 800 °C, with a 2-hour hold, controlled by a hot air gun. The capillary was rocked during heating to improve sample averaging, allowing differently oriented grains to contribute to the diffraction pattern and to prevent hotspot formation. XAS data were collected in transmission mode at the Ru K-edge during isothermal sections after each 100 °C increase, with three spectra merged at each temperature. PXRD data were collected continuously during the ramp at 23 keV, with data shown for every 50 °C increase.

XAS data analysis and fitting were performed using the Athena and Artemis packages of the Strawberry Demeter software suite.[1] Co^0 and Ru^0 foils were measured to calculate the $S0^2$ value for EXAFS fitting. For PXRD analysis, wavelength and sample-detector distances were corrected during pre-processing, and Rietveld refinements were conducted using GSAS II,[2] with the broad carbon peak at low angles included in the background (using a Chebyshev-1 function).

H/D experiments

To conduct the isotopic exchange experiments, the same $H_2 + D_2$ flow is first directed straight to the mass spectrometer, bypassing the sample, until a stable signal is obtained. Subsequently, the flow is redirected through a bypass to pass through each catalyst. The amount of HD produced by each catalyst directly depends on the amount of H_2. Therefore, normalization is performed using the H_2 value before opening the bypass, as this ensures the most reproducible experimental conditions and more reliable results. The analysis of hydrogen and deuterium was carried out using a Pfeiffer Vacuum mass spectrometer calibrated for this purpose.

2. Characterization of the precursors

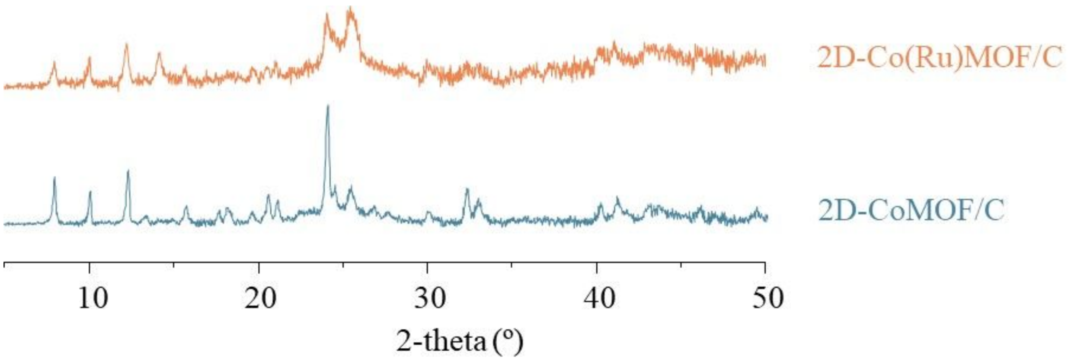

Figure S1. X-Ray diffraction patterns of the MOFs supported on carbon precursors before pyrolysis.

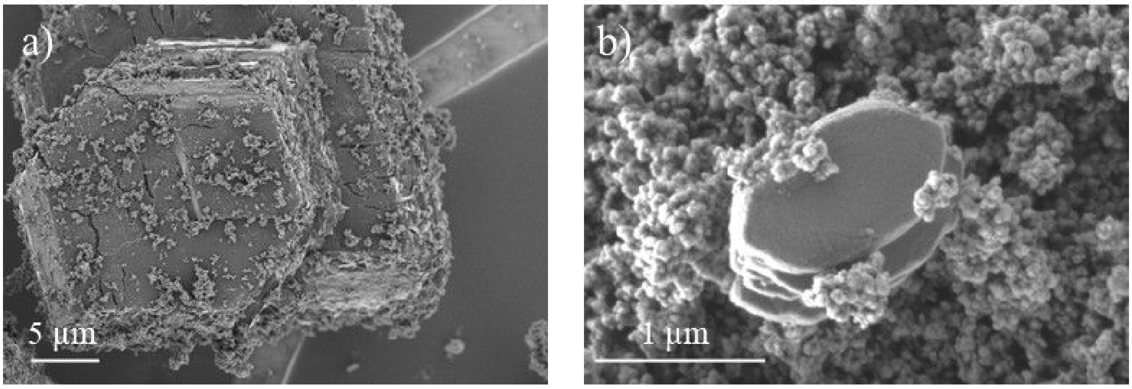

Figure S2. FESEM images of a) 2D-CoMOF/C and b) 2D-Co(Ru)MOF/C.

Table S1. EXAFS fitting parameters at the Co K-edge for 2D-CoMOF/C and 2D-Co(Ru)MOF/C. The fitted scattering paths include O.1, corresponding to a Co–O path as in CoO.

Sample	Enot	Path	C.N.	σ^2	R (Å)	R-factor
**2D-CoMOF/C**	-1.94 ± 0.49	O.1	4.81 ± 0.22	0.0051 ± 0.001	2.081	0.024
**2D-Co(Ru)MOF/C**	-2.70 ± 0.68	O.1	5.48 ± 0.33	0.0059 ± 0.001	2.084	0.011

S_0^2 refined using Co foil to 0.78. K range2-15, Rwindow 1-2, Nipd 7.6

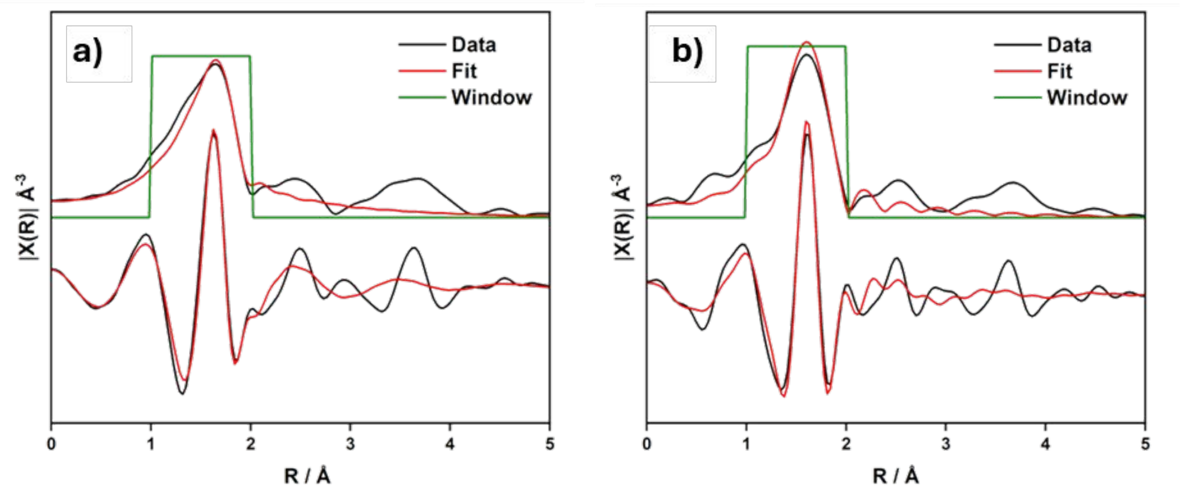

Figure S3. Co K-edge EXAFS fits in R magnitude and real space. a) 2D-CoMOF/C and b) 2D-Co(Ru)MOF/C. The corresponding fitting parameters are provided in Table S1.

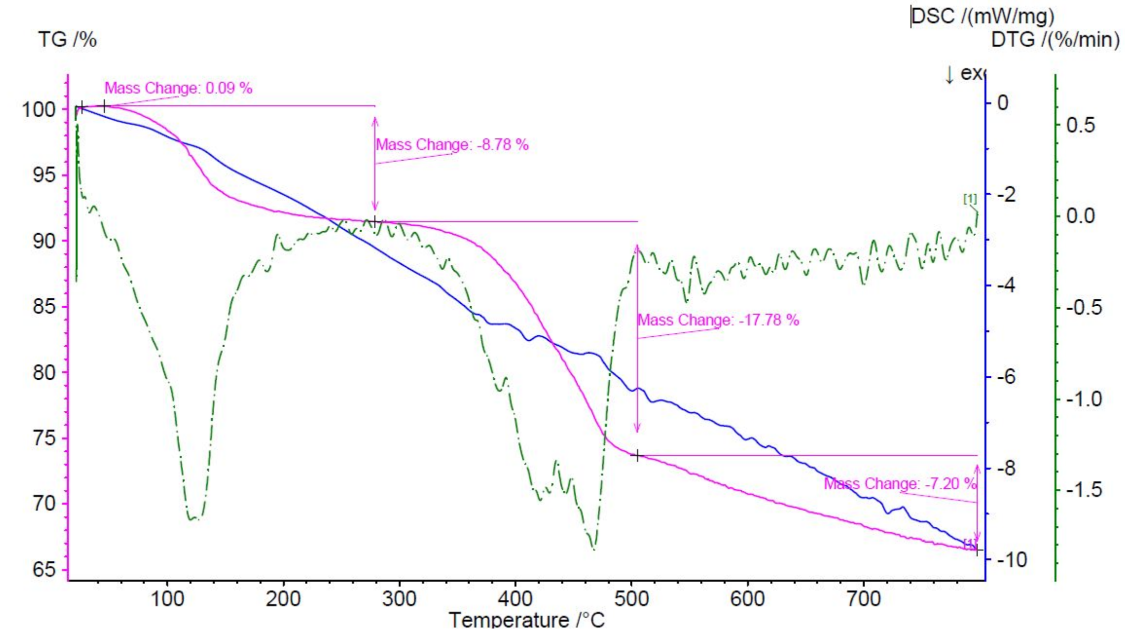

Figure S4. Thermogravimetric analysis of the 2D-CoMOF/C.

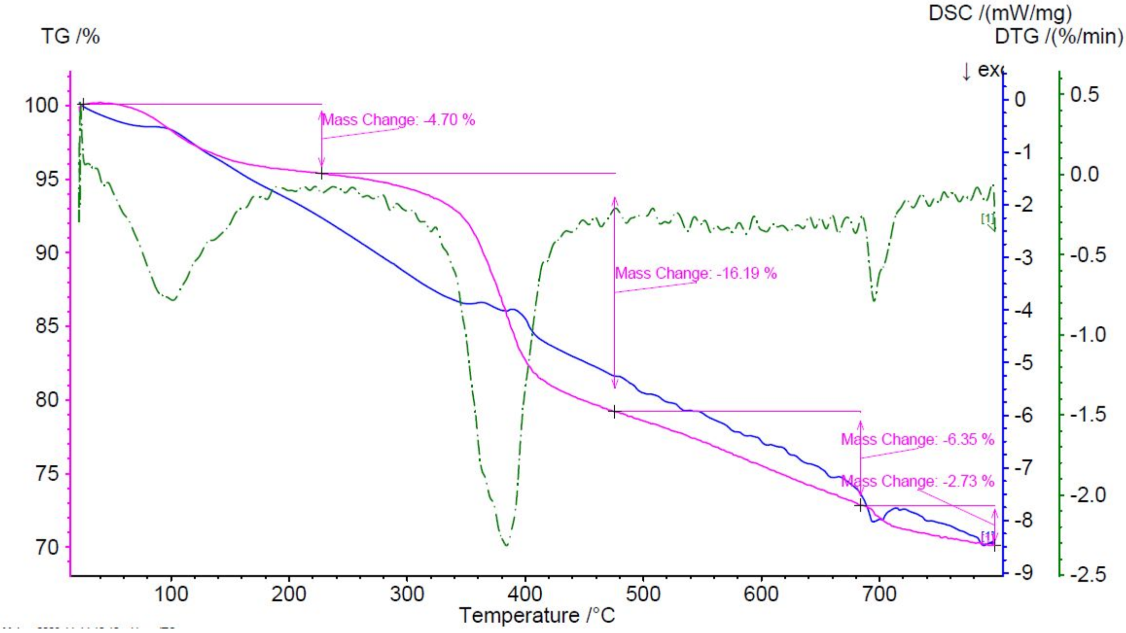

Figure S5. Thermogravimetric analysis of the 2D-Co(Ru)MOF/C.

3. Catalysts characterization

Table S2. EXAFS fitting parameters at the Co K edge of the monometallic **Co@C** and bimetallic **Co-Ru@C** catalysts post-pyrolysis. The fitted scattering paths include Co.1, a Co-Co path in Co^0.

Sample	Enot	Path	C.N.	σ^2	R (Å)	R-factor
**Co@C**	-5.60 ± 0.24	Co.1	9.50 ± 0.21	0.0062 ± 0.0002	2.505	0.005
**Co-Ru@C**	-5.90 ± 0.51	Co.1	10.46 ± 0.21	0.0063 ± 0.0002	2.505	0.005

S_0^2 refined using Co foil to 0.78. K range 3-15, Rwindow 1-3, Nipd 15.3

Note from the table above that the Co-Co distance in the monometallic and bimetallic catalysts are identical, hence no alloying is indicated from the perspective of an absorbing Co. The Ru is 10 times lower in concentration than the Co, however, so it would alter the average metallic distance very little, if at all.

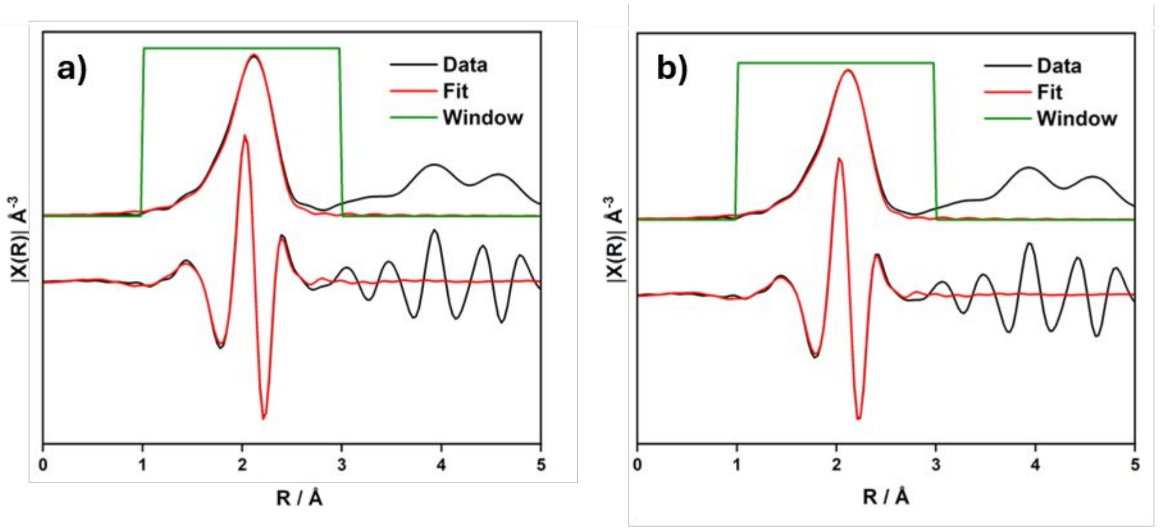

Figure S6. Co K-edge EXAFS fits in R magnitude and real space. a) **Co@C** and b) **Co-Ru@C**. The corresponding fitting parameters are provided in Table S2.

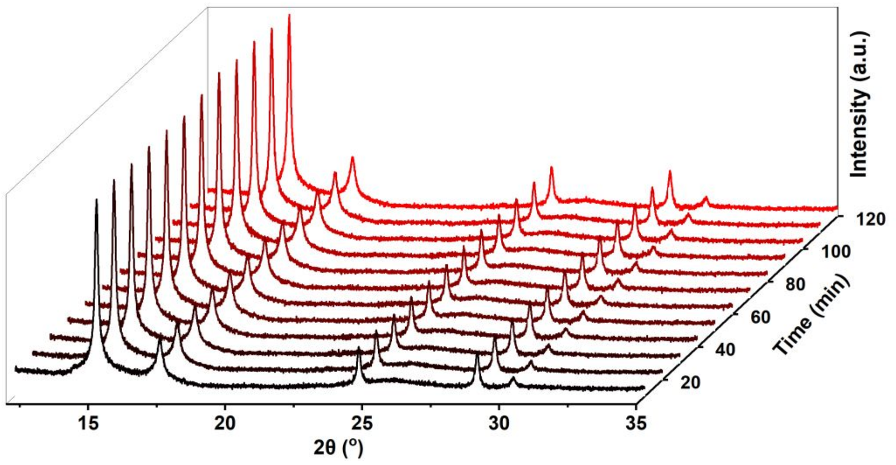

Figure S7. *In situ* PXRD collected at 23 keV during the 2-hour dwell at 800°C in N_2 for the bimetallic Co-Ru sample.

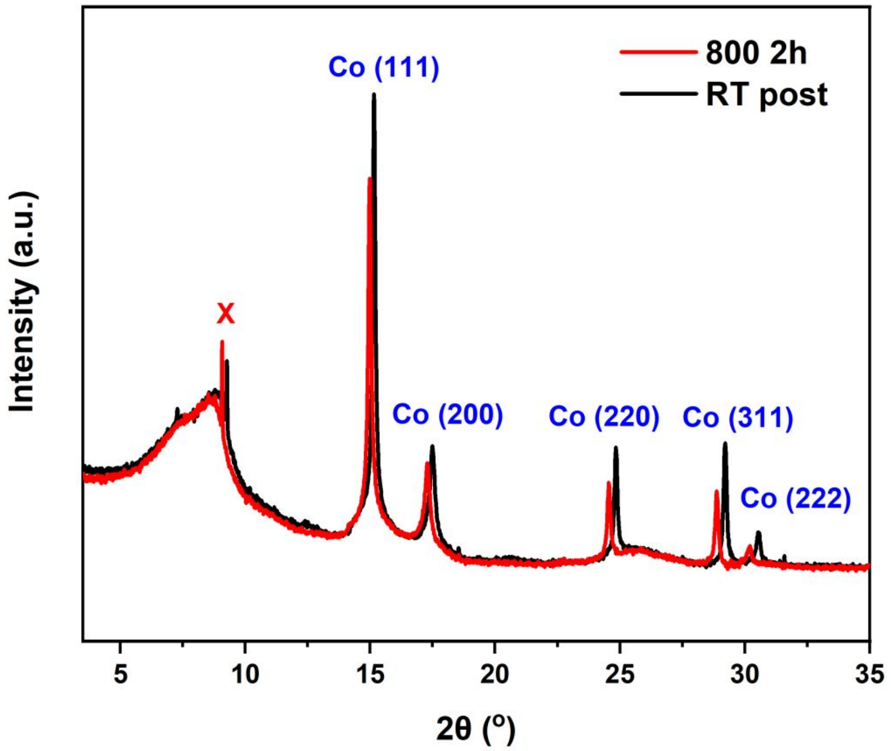

Figure S8. Comparison of raw PXRD data collected at 800°C at the end of the 2-hour hold and after cooling to room temperature, both under N_2 for the bimetallic Co-Ru sample. The peak shift is attributed to thermal contraction upon cooling.

Refinement process and values obtained for the above samples:

During a refinement, the background was fitted to a Chebyshev-1 function with 5 coefficients, and one background peak was added to account for the amorphous carbon reflection at 12/25°, depending on the energy. For the Co FCC phase, the unit cell, crystallite size, and U_{iso} of the Co atom were refined sequentially and then in groups. A March-Dollase preferred orientation model

was applied and the M-D ratio along the (111) refined. This was to mimic the effects of stacking faults and improve the fit. The goodness of fit is reported by the weight of residual, wR. Values are reported in the table below.

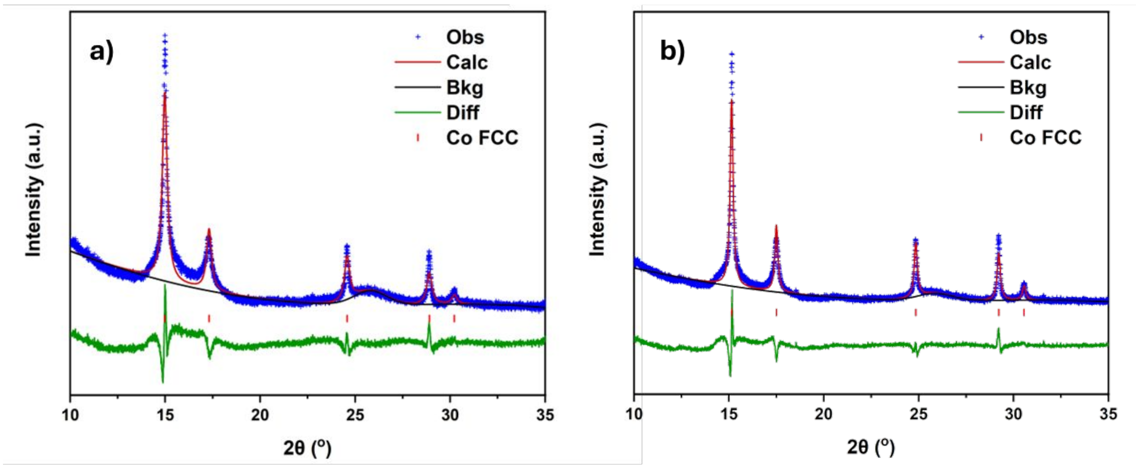

Figure S9. Refined PXRD patterns of the bimetallic **Co-Ru@C** catalyst measured in situ at 23 keV: (a) at 800°C during pyrolysis and (b) after cooling to room temperature. Refinement details are provided in Table S3.

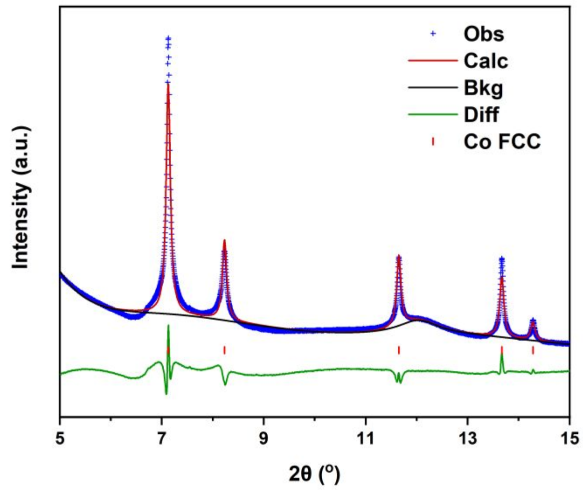

Figure S10. Refined PXRD pattern of **Co@C** measured *ex situ* after pyrolysis. Note that this data originates from the same sample but was collected at BM31 (ESRF) at 70 keV, resulting in different 2θ values and data resolution. The refinement was conducted using the same methodology. Refinement details are provided in Table S3.

Table S3. Refinement parameters obtained from synchrotron powder XRD data for in situ and ex-situ measurements of **Co@C** and **Co-Ru@C**, recorded during and/or after pyrolysis.

Sample	Unit cell (Å)	Size (nm)	U_{iso} Co	M-D ratio	wR (%)
**Co-Ru@C**, *in situ* at 800°C in N_2	3.576	10.4	0.0266	1.52	5.7
**Co-Ru@C RT** post *in situ* pyrolysis	3.538	17.2	0.0090	1.48	5.8
**Co@C RT** post *in situ* pyrolysis	3.546	15.0	0.0088	1.51	3.8

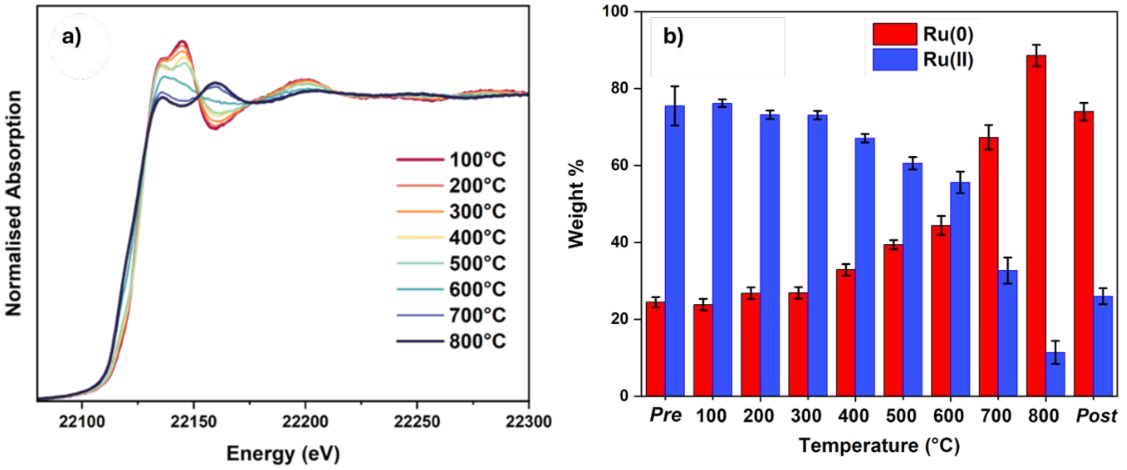

Figure S11. a) XANES spectra recorded for the 2D-Co(Ru)MOF/C sample during a temperature ramp to 800°C under N_2, simulating the pyrolysis conditions used in the synthesis of **Co-Ru@C**. b) Evolution of the weight fractions of Ru(0) and Ru(II), determined by linear combination fitting (LCF) of the XANES spectra using $Ru(OAc)_2$ as a reference, before the *in situ* experiment, during the temperature ramp in N_2, and after cooling to room temperature (RT) in the same atmosphere.

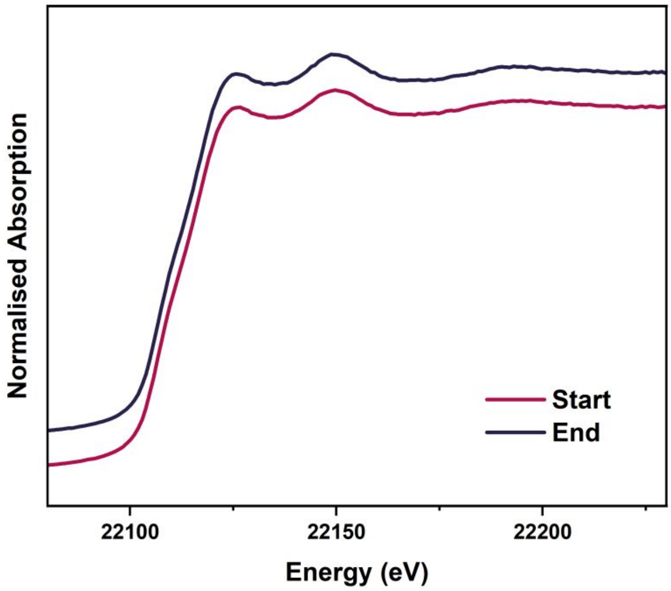

Figure S12. Merged XANES of **Co-Ru@C** at the Ru K edge from the start (in red) vs the end (in black) of a 2 h isothermal hold at 800 ºC. Vertical stacked for clarity, as spectra overlap perfectly. Whiteline energy identical at 22115.4 eV.

Evidence for Co-Ru interaction and resulting EXAFS fitting parameters

The interaction between Ru and Co is evidenced in both the wavelet transform analysis and the χ(k) data. In the *k* and *R* space, the wavelet analysis (Figure S13 a-d) reveals an additional component separating from the Ru–Ru contribution, located at slightly lower distances and identified as Ru–Co. This feature does not correspond to the expected FT position of Ru–O or Ru–O–Ru paths, and its intensity is significant, especially considering the relatively small Ru–O contribution in the pyrolyzed sample.

The χ(k) data (Figure S13 e-f) also show contributions from metallic Co at approximately 7 $Å^{-1}$ and 11 $Å^{-1}$, indicative of Ru–Co paths. These features cannot be explained solely by metallic Ru foil and occur at *k*-values too high to be attributed to partially oxidized Ru, whose contributions appear between ~3 and 6.5 $Å^{-1}$. Furthermore, anti-phasic interactions between the Co and Ru metallic paths are likely responsible for the notably low intensity observed in the Fourier transform. Crystallite sizes estimated by PXRD (~12 nm) would typically result in higher intensities between 2–2.8 Å in R-space. In addition, a sharp and unusual node at 1.5 Å (not phase-corrected, Figure 2d in the main text) is also thought to arise from anti-phasic interactions, occurring at interatomic distances too long to be solely due to Ru–O coordination. Fitting of the Fourier transform was only possible upon inclusion of a Ru–Co path, alongside RuO_x and Ru^0

components. Altogether, these findings strongly support the presence of Ru atoms in close proximity to Co, with direct interactions between the two.

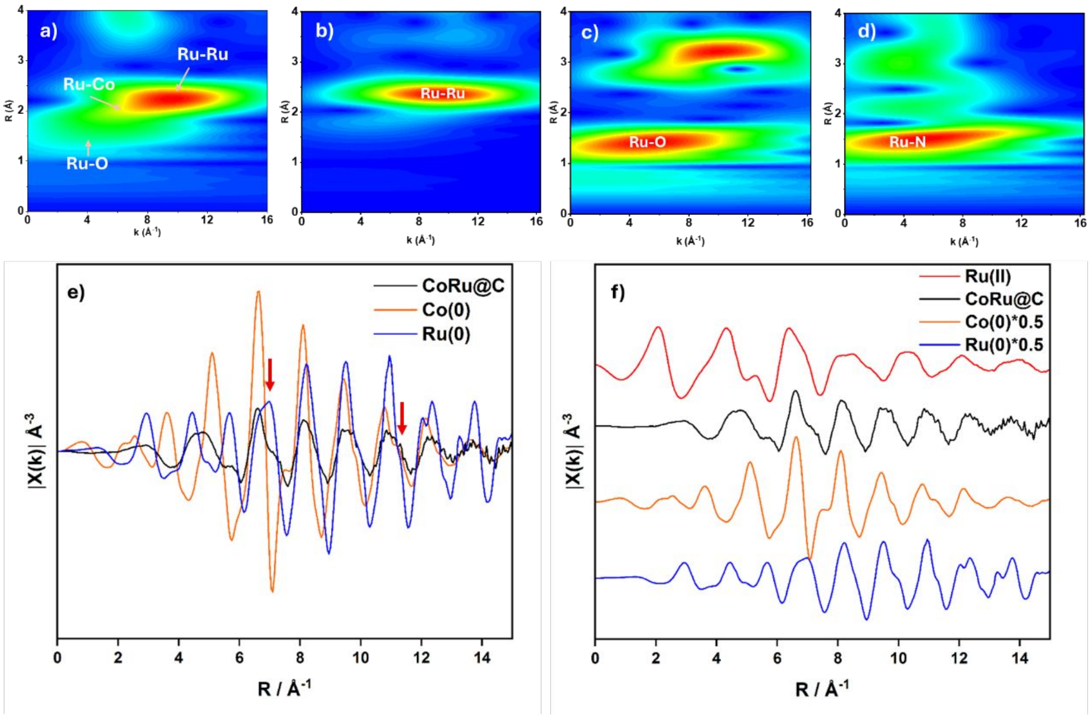

Figure S13. The wavelet transform analysis for the Ru K-edge EXAFS spectra of pyrolyzed (a) **Co-Ru@C**, compared to references (b) Ru(0) (foil) (c) RuO_2 and (d) $Ru(bpy)_2$. Analysis used the Larch package.[3] (e) Overlapped and (f) stacked and scaled k^2 weighted x(k) data for the experimental Ru K spectra of **CoRu@C**, with a Ru foil and Ru(II) complex references and a Co foil reference at the Co K edge. Red arrows highlight areas in inverse angstroms where the influence of paths from the Co foil are observed in the material.

Two models were therefore tested to fit the **Co-Ru@C** after cooling, both with 50% of Ru atoms substituted by Co, but with either a *hcp* or *fcc* crystal structure. Ru(0) and Co(0) have, respectively, a *hcp* and *fcc* crystal structure at room temperature. A Ru-O path from RuO_2 was also added to both to account for the partially reoxidised Ru(0). The calculated atomic distances are provided in Tables S4 and S5. Both models provided good EXAFS fits (Figure S14). While the calculated interatomic distances were closer to those predicted by the hcp model, the lack of reflections of a hcp phase in the PXRD (despite the high coordination numbers in the EXAFS, see Table S6) implies that Ru is more likely incorporated into the Co *fcc* phase during pyrolysis.

Co doped into hcp Ru^0 lattice (fit shown in Figure S14. b)

Table S4. EXAFS at the Ru K edge of the 2D-Co(Ru)MOF/C from in situ pyrolysis to become **Co-Ru@C**. These spectra were measured after the sample was cooled from 800°C to RT, in N_2. The model used for the metallic component was of hcp Ru^0 with 50% of atoms substituted for Co. The σ^2 value for O is high, however, it was fit using a model of ordered RuO_2 which is unlikely to represent well the oxide component present. Only 1st shell components were fit.

Enot	Path	C.N.	σ²	R (Å)	R-factor
-4.08 ± 0.40	Ru-O	1.92 ± 0.15	0.0047 ± 0.001	1.973	0.008
	Ru-Ru	4.09 ± 0.20	0.0064 ± 0.000	2.663	
	Ru-Co	2.42 ± 0.14	0.0057 ± 0.001	2.544	

Amp = 0.78, N_{ipd} = 13.75, k-range 3 – 13.8Å^{-1}, R-window 1-3Å

Ru doped into fcc Co^0 lattice (fit shown in Figure S14. d)

Table S5. EXAFS at the Ru K edge of the 2D-Co(Ru)MOF/C from in situ pyrolysis to become **Co-Ru@C**. These spectra were measured after the sample was cooled from 800°C to RT, in N_2. The model used for the metallic component was of FCC Co with 50% of Ru atoms substituted. The σ^2 value for O is high, however, it was fit using a model of ordered RuO_2, which is unlikely to represent well the oxide component present. Only 1st shell components were fit.

Enot	Path	C.N.	σ²	R (Å)	R-factor
-4.08 ± 0.40	Ru-O	1.95 ± 0.13	0.0051 ± 0.001	1.977	0.007
	Ru-Ru	4.20 ± 0.16	0.0066 ± 0.000	2.546	
	Ru-Co	2.46 ± 0.12	0.0057 ± 0.000	2.661	

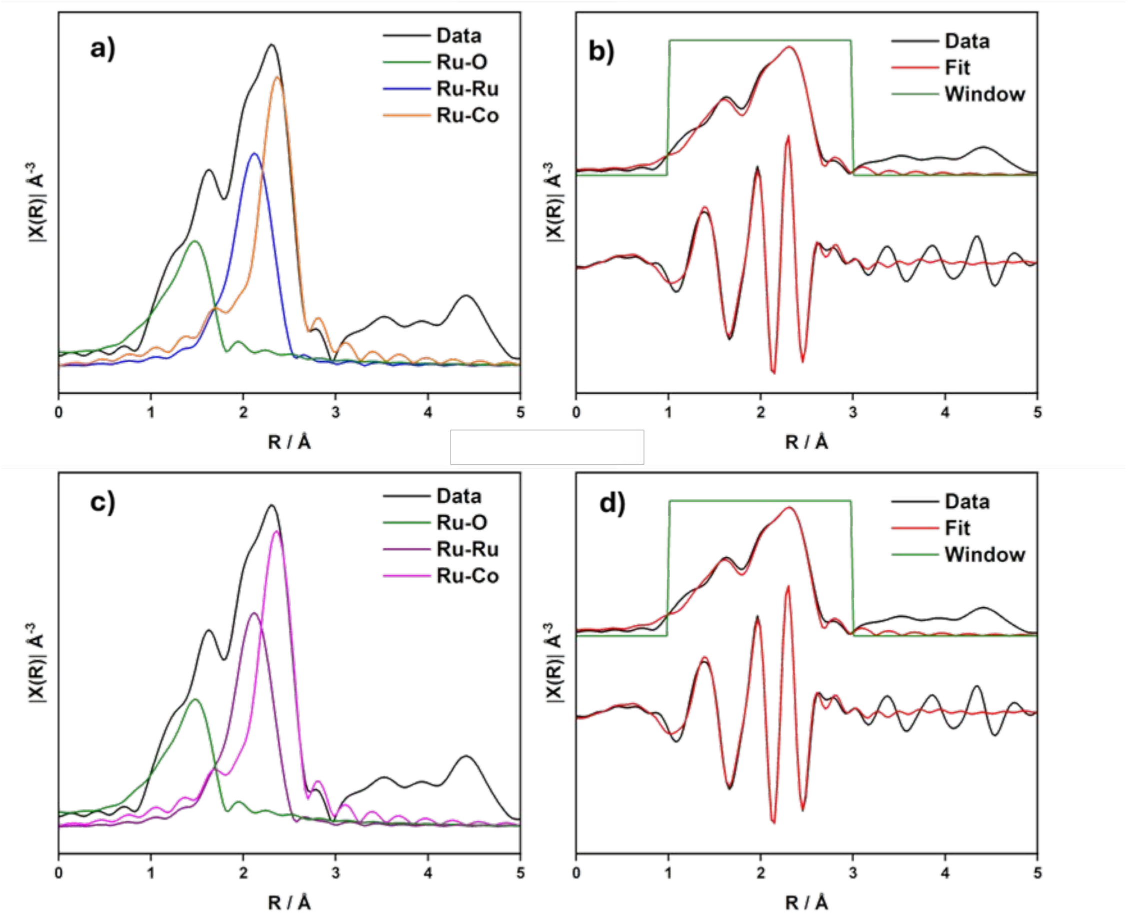

Figure S14. Comparison of the two models used to fit the Co-Ru data at the Ru K edge. (a, b) Scattering paths and EXAFS fit using an hcp Ru^0 model with Co atom substitution. (c, d) Scattering paths and EXAFS fit using an fcc Co^0 model with Ru as the absorbing atom.

Table S6. Comparison of goodness of fit and delta R values for the two models. *Note that Ru increases the size of the Co lattice. Co decreases the size of the Ru lattice.*

Enot	Path	ΔR (Å) *(versus undoped lattice)*	R (Å)	R-factor
Ru doped into FCC Co	Ru-Ru	0.041	2.546	0.008
	Ru-Co	0.156	2.661	
Co doped into HCP Ru	Ru-Ru	0.013	2.663	0.007
	Ru-Co	-0.106	2.544	

4. Study of other solvents

Table S7. Study of other solvents in the reductive coupling of 1,2-dinitrobenzene and benzaldehyde.[a]

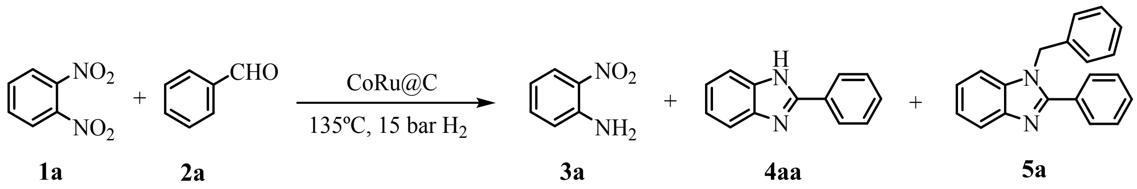

Entry	Solvent	Conversion 1a (%)	Selectivity (%)		
			3a	4aa	5a
1	Toluene	>99	0	50	50
2	THF	>99	0	92	8
3	AcOEt	>99	2	98	0
4	iPrOH	>99	0	>99	0
5	EtOH	>99	0	>99	0
6	H_2O	>99	0	>99	0

[a]Reaction conditions: **1a** (1 mmol), **2a** (1.5 mmol), **Co-Ru@C** (10 mg), solvent (3 mL), 16 h.

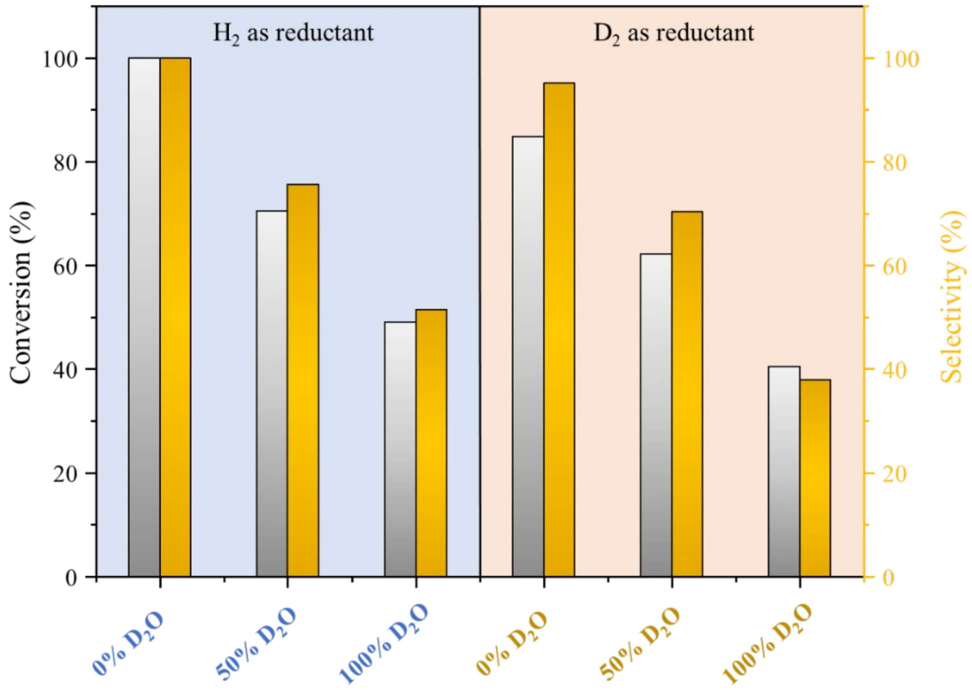

Figure S15. Conversion and selectivity to 2-phenylbenzimidazole (4aa) in the reductive coupling of 1,2-dinitrobenzene and benzaldehyde using H_2 (blue region) and D_2 (orange region) as reductant agents and different ratios of % D_2O as solvent ($D_2O + H_2O = 100\%$).

5. Unravelling the active species

UV-vis spectroscopy studies were carried out to calculate the adsorption of 1,2-nitroaniline (3a) and 2-phenylbenzimidazole (4aa) on **Co@C** and **Co-Ru@C** catalysts. First, we made a calibration with concentrations of 2 ppm, 4 ppm, 8 ppm and 12 ppm of 1,2-nitronaline and 2-phenylbenzimidazole to determine their respective absorptivity coefficients (e) by the Beer-Lambert equation:

$$A = \varepsilon \cdot l \cdot C$$

Where A is the absorbance, l is the optical path length and C is the concentration of the substrate. Once ε has been calculated, we prepared 8 ppm solutions of 1,2-nitroaniline and 2-phenylbenzimidazole. We add 10 mg of each tested catalyst to 10 mL of each substrate solution. We left it under constant stirring for 16 hours and then filtered the catalyst to determine the absorbance value of the solution. We can obtain the concentration (ppm) of the substrate remaining in the solution through interpolation and, in this way, the amount of 1,2-nitroaniline and 2-phenylbenzimidazole adsorbed by the catalyst (mg/g_{cat}). As the support is carbonaceous, all data are normalized by subtracting the absorbance signal from the support to each catalyst.

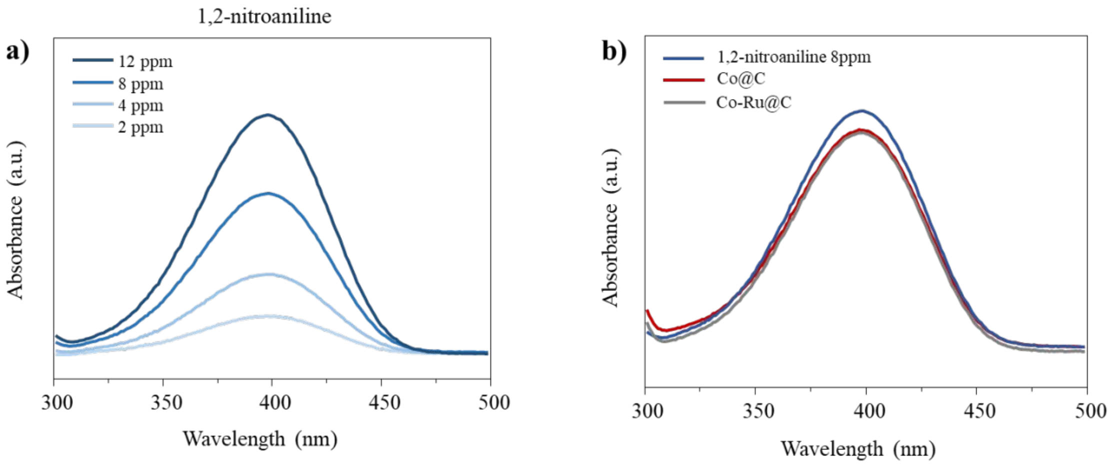

Figure S16. a) UV-vis calibration curves of 1,2-nitroaniline (**3a**) at different concentrations (12, 8, 4 and 2 ppm). b) UV-vis spectra of the solutions before (blue) and after (red and grey, respectively) adsorption of 1,2-nitroaniline (**3a**) by **Co@C** and **Co-Ru@C** catalysts.

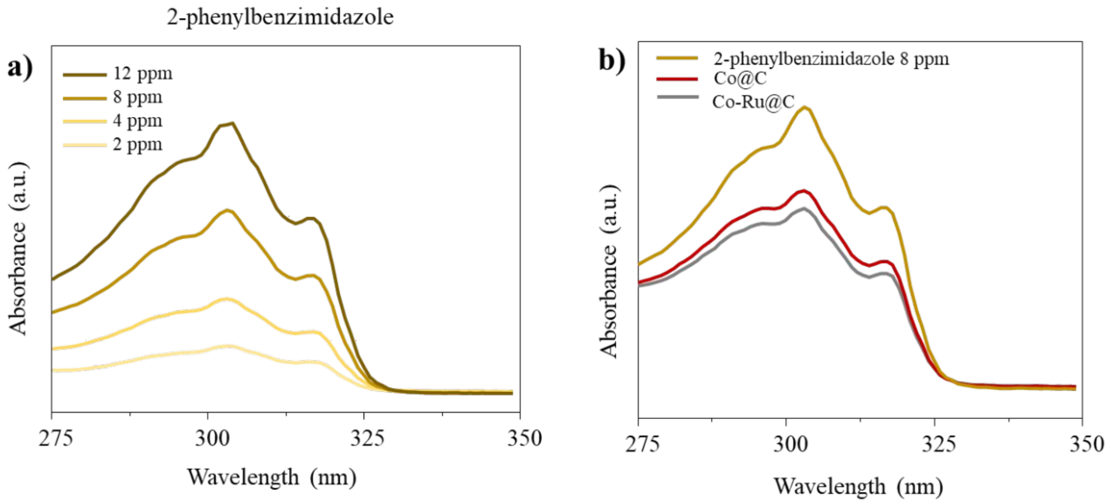

Figure S17. a) UV-vis calibration curves of 2-phenylbenzimidazole (**4aa**) at different concentrations (12, 8, 4 and 2 ppm). b) UV-vis spectra of the solutions before (yellow) and after (red and grey, respectively) adsorption of 2-phenylbenzimidazole (**4aa**) by **Co@C** and **Co-Ru@C** catalysts.

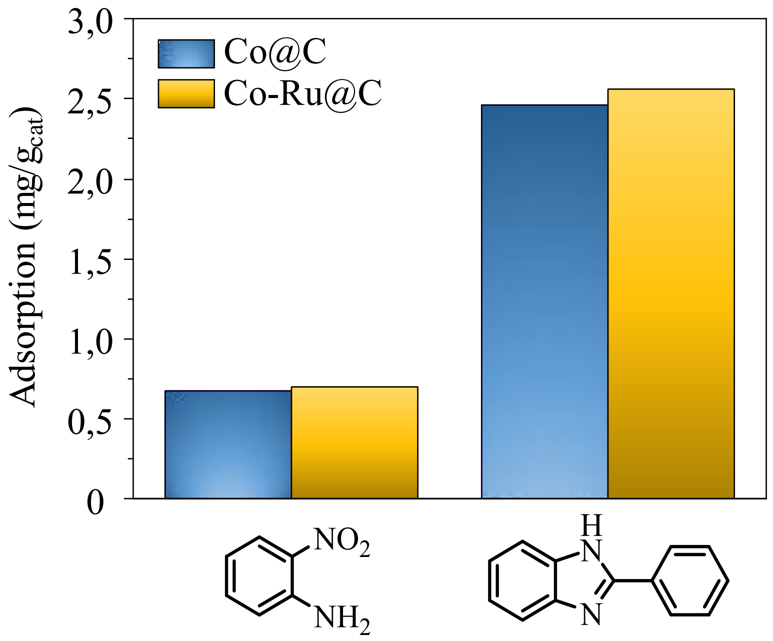

Figure S18. Adsorption capacities of **Co@C** and **Co-Ru@C** catalysts to 1,2-nitroaniline (**3a**) and 2-phenylbenzimidazole (**4aa**).

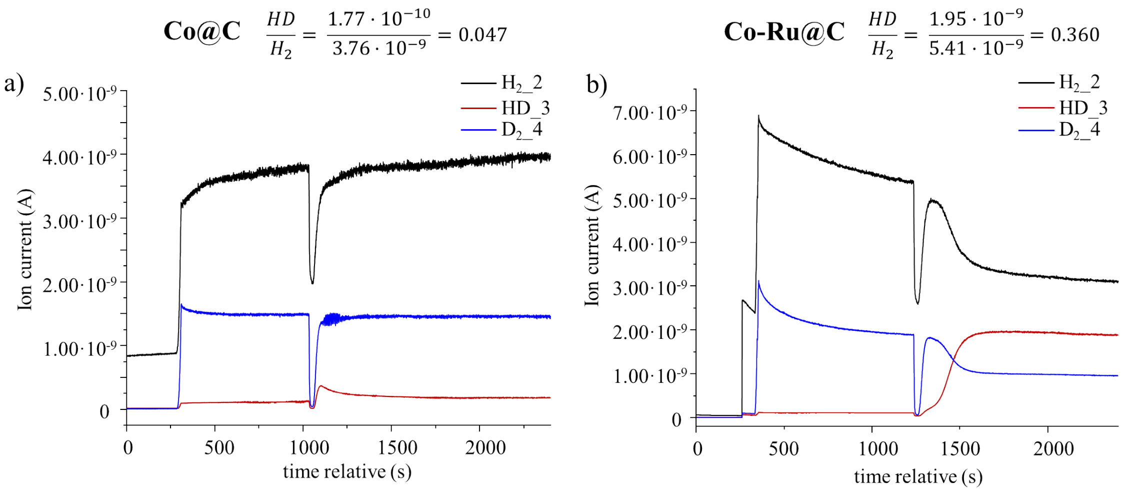

Figure S19. Time-resolved mass spectra to determine the ratio HD/H_2 for a) **Co@C** and b) **Co-Ru@C** catalyst.

6. Catalyst stability

Table S8. Reusability study data with full conversion and selectivity.

Run	Conversion (%)	Selectivity to 3a (%)	Selectivity to 4aa (%)	Co metal leached (µg)[a]	Ru metal leached (µg)[a]
1	> 99	< 1	> 99	14	0
2	> 99	< 1	> 99	8	0
3	> 99	< 1	> 99	9	1
4	> 99	4	96	7	1
5	>97	2	98	8	1

[a]The reaction mixture was filtered and the crude was analysed through ICP-AES to determine the amount of leached cobalt and ruthenium. 10 mg of **Co-Ru@C** catalyst has 970 µg of Co and 80 µg of Ru.

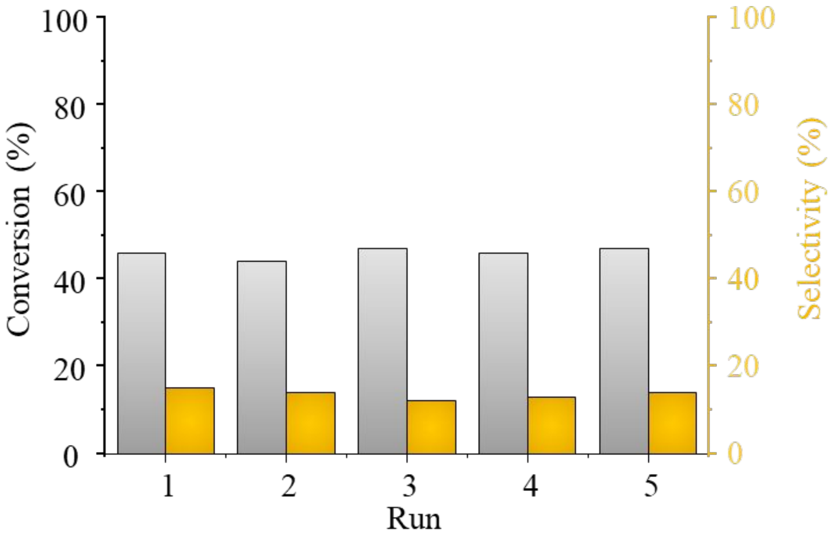

Figure S20. Conversion of 1,2-dinitrobenzene **1a** (grey) and selectivity to 2-phenylbenzimidazole **4aa** (orange) for five reaction runs using **Co-Ru@C** as catalyst at incomplete conversion. Reaction conditions: **1a** (1 mmol), **2a** (1.5 mmol), H_2O (3 mL), 135ºC, 15 bar H_2, 3 h.

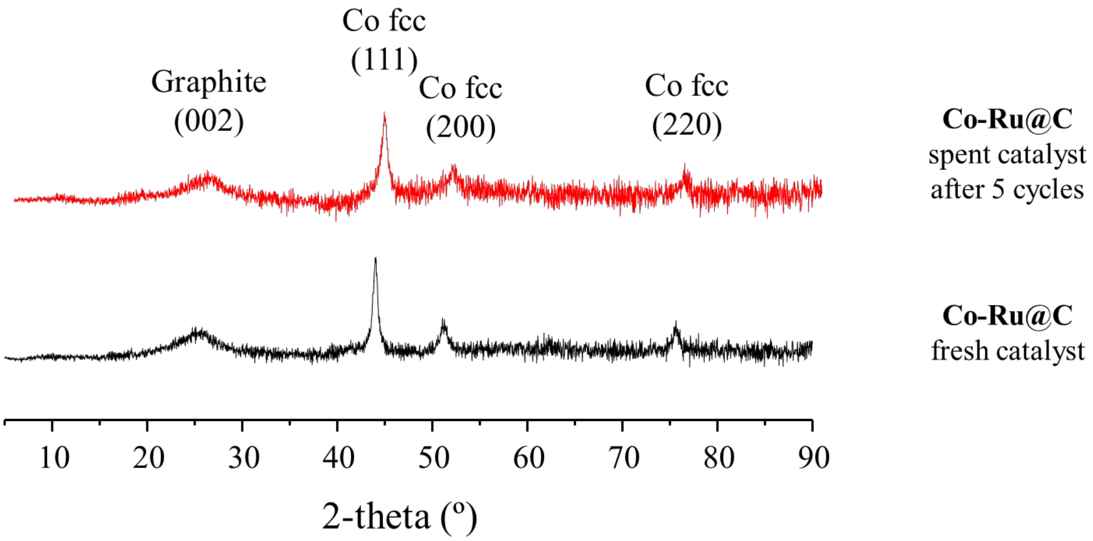

Figure S21. Power X-ray diffraction patterns of **Co-Ru@C** fresh catalyst and **Co-Ru@C** spent catalyst after 5 cycles.

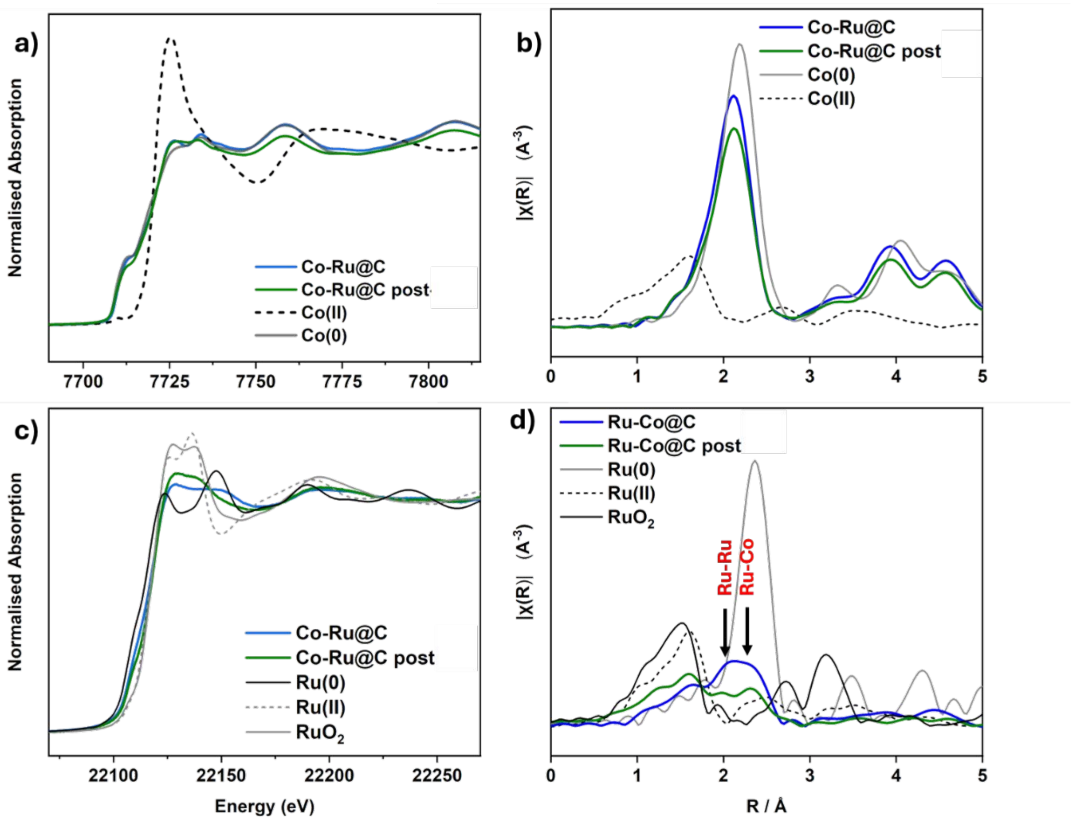

Figure S22. a,c) XANES spectra and b,d) k²-weighted Fourier transform in R-space for the pyrolysed **Co-Ru@C** and the same sample after reaction. Data were collected *ex situ* at (top) the Co K edge, and (bottom) the Ru K edge. Note Ru data has an additional reference of RuO_2, as linear combination fitting showed it is a component of the post-reaction sample. Also, the change in intensity of the EXAFS oscillations at the Co K edge is due to measurement at different synchrotrons. No increase in whiteline intensity, which would indicate oxidation, is observed.

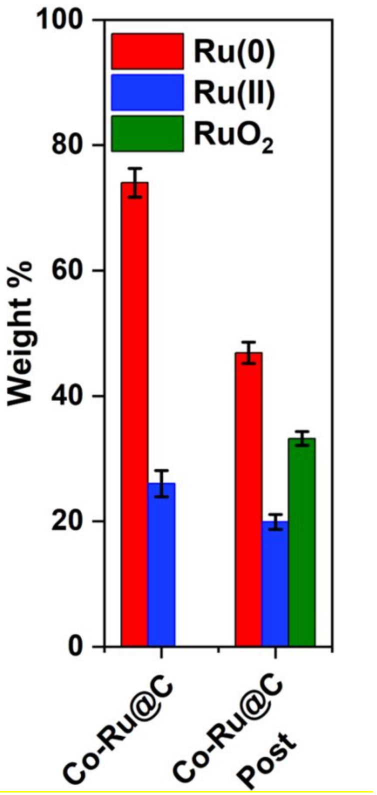

Figure S23. Weight fractions of Ru(0), Ru(II), and RuO_2 determined by linear combination fitting (LCF) of the XANES spectra using $Ru(OAc)_2$ as the reference for Ru(II).

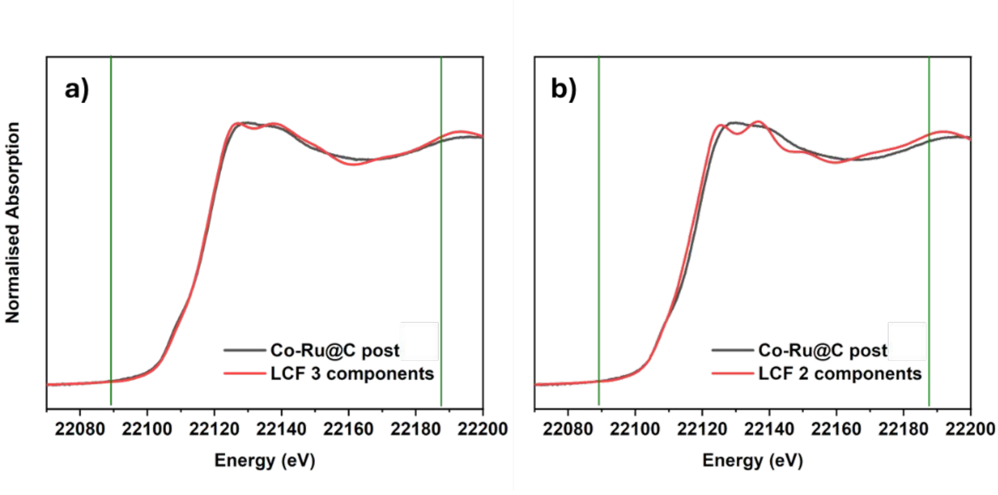

Figure S24. Comparison of the quality of the linear combination fits of **Co-Ru@C** after reaction using a) Ru(0), Ru(II) and RuO_2, or b) Ru(0) and Ru(II) only. Note that 3 components gave the best fit, and component weight percents were extracted from this fit.

Table S9. Linear combination fitting results for **Co-Ru@C** and **Co-Ru@C** post-reaction, including errors. Fits were in the range -20 to +50 eV, and all weights were forced between 0-1. The reference spectra for Ru(II) was $Ru(OAc)_2$

	**Ru(0)**	**Ru(II)**	**RuO_2**
**Co-Ru@C**	74.0 ± 2.3	26.0 ± 2.1	-
**Co-Ru@C** post-reaction	46.9 ± 1.7	19.9 ± 1.2	33.2 ± 1.1

7. Green Metrics calculations

The metrics of Green Chemistry and Sustainability have been calculated using the work of Roger A. Sheldon[4]. The metrics employed in this work have been calculated using the following formulas:

$$E = \frac{total\ mass\ of\ waste}{mass\ of\ final\ product}$$

$$PMI\ (Process\ Mass\ Intensity) = \frac{total\ mass\ in\ process}{mass\ of\ final\ product}$$

$$SI\ (Solvent\ Intensity) = \frac{mass\ of\ solvents}{mass\ of\ product}$$

$$RME\ (Reaction\ Mass\ Eficiency) = \frac{mass\ of\ product}{total\ mass\ of\ reactants} \cdot 100$$

The results obtained from the study using **Co-Ru@C** catalyst are presented in Table S10.

Table S10. Green Chemistry parameters calculated for the reaction using **Co-Ru@C** catalyst.

Green Metric	**Co-Ru@C** catalyst
E	16.2
PMI	17.2
SI	15.4
RME (%)	59.2

Regarding EcoScale tool, the assessment of the rubric evaluating the various parameters related to the sustainability of a chemical reaction for this catalyst is presented in Table S11. Each parameter has been evaluated in order to determine its penalization. EcoScale score is calculated by subtracting the total penalty points of each solvent from 100 points.

Table S11. EcoScale evaluation for the reaction using **Co-Ru@C** catalyst.

Parameter	**Co-Ru@C** catalyst
1. Yield	0
2. Price of reaction components	3
3. Safety	0
4. Technical setup	3
5. Temperature/time	4
6. Workup and purification	0
Total penalty points	10
**EcoScale score**	**90**

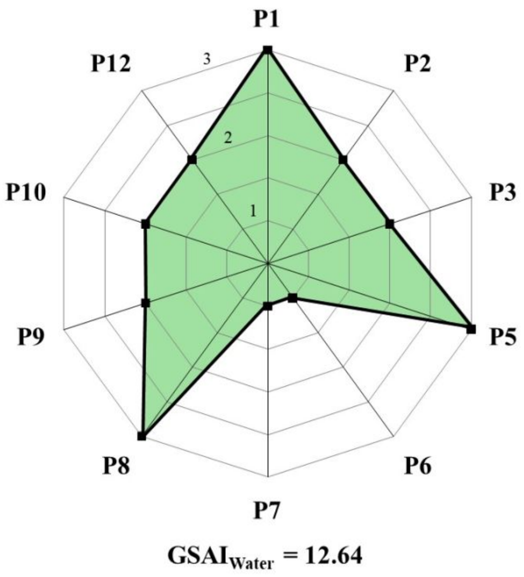

Figure S25. Representation of the GreenStar metrics for the synthesis of 1,2-phenylbenzimidazole (4aa) from 1,2-dinitrobenzene (1a) and benzaldehyde (2a) using **Co-Ru@C** catalyst.

8. Characterization data and experimental details of the isolated benzimidazoles

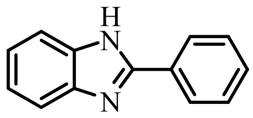

2-phenyl-1*H*-benzo[d]imidazole (4aa):

Yield: 95%. ^{1}H NMR (401 MHz, MeOD) δ: 8.02 (dd, J = 7.6, 1.7 Hz, 2H), 7.54 (dd, J = 6.0, 3.2 Hz, 2H), 7.48 – 7.42 (m, 3H), 7.19 (dd, J = 6.0, 3.2 Hz, 2H). ^{13}C NMR (101 MHz, MeOD) δ: 153.37, 140.26, 131.35, 131.01, 130.14, 127.81, 123.93, 115.87. MS (EI): m/z (rel. int.) 194.[5]

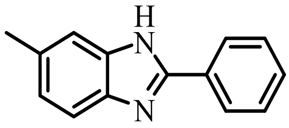

4-methyl-2-phenyl-1*H*-benzo[d]imidazole (4ba):

Yield: 90%. ^{1}H NMR (401 MHz, MeOD): δ: 8.09-8.06 (m, 2H), 7.56 – 7.48 (m, 4H), 7.40 (s, 1H), 7.11 (d, *J* = 8.1 Hz, 1H), 2.48 (s, 3H). ^{13}C NMR (101 MHz, MeOD) δ: 153.06, 138.54, 134.03, 131.26, 130.15, 129.42, 127.71, 125.50, 116.00, 115.27, 21.73. MS (EI): m/z (rel. int.) 208.[5]

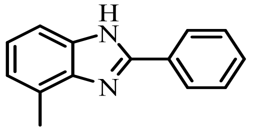

4-methyl-2-phenyl-1*H*-benzo[d]imidazole (4ca):

Yield: 98%. ^{1}H NMR (300 MHz, MeOD): δ: 8.13 – 8.011 (m, 2H), 7.56 – 7.41 (m, 3H), 7.42 (d, *J* = 7.7 Hz, 1H), 7.16 – 7.12 (m, 1H), 7.05 – 7.03 (m, 1H), 2.62 (s, 3H). ^{13}C NMR (75 MHz, MeOD): δ: 153.20, 133.35, 131.32, 131.22, 130.61, 130.08, 129.16, 128.05, 124.50, 123.94, 113.68, 17.17. MS (EI): m/z (rel. int.) 208.[5]

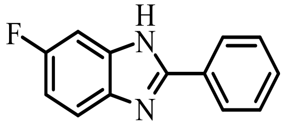

6-fluoro-2-phenyl-1*H*-benzo[d]imidazole (4da):

Yield: 75%. ^{1}H NMR (300 MHz, MeOD) δ: 8.06 (dd, *J* = 10.6, 4.4 Hz, 2H), 7.59 – 7.52 (m, 4H), 7.29 (dd, *J* = 9.0, 2.1 Hz, 1H), 7.08 – 7.01 (m, 1H). ^{13}C NMR (75 MHz, MeOD) δ: 161.15 [162.72, 159.57 (d, ^1JC-F = 237.5 Hz)], 154.74, [140.64, 140.41 (d, ^3JC-F = 12.9 Hz)], 136.43, 134.00,

131.57, 130.76, 130.22, 129.55, 127.79, [116.83, 116.66 (d, ^{3}JC-F = 10.1 Hz)], [112.22, 111.87 (d, ^{2}JC-F = 25.8 Hz)], [101.90, 101.52 (d, ^{2}JC-F = 26.1 Hz)]. MS (EI): m/z (rel. int.) 212.[5]

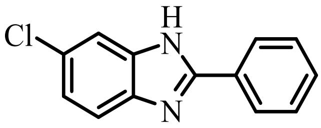

6-chloro-2-phenyl-1*H*-benzo[d]imidazole (4ea):

Yield: 83%. ^{1}H NMR (401 MHz, MeOD) δ: 8.08 (dd, *J* = 7.7, 1.8 Hz, 2H), 7.60 – 7.53 (m, 5H), 7.25 (dd, *J* = 8.6, 1.9 Hz, 1H). ^{13}C NMR (101 MHz, MeOD) δ: 154.50, 141.31, 138.49, 131.51, 130.38, 130.02, 129.21, 127.07, 124.41, 116.50, 114.84. MS (EI): m/z (rel. int.) 228.[5]

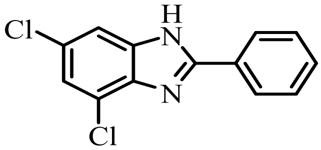

4,6-dichloro-2-phenyl-1*H*-benzo[d]imidazole (4fa):

Yield: 73%. ^{1}H NMR (401 MHz, MeOD) δ: 8.13 – 8.09 (m, 2H), 7.55 – 7.53 (m, 4H), 7.28 (d, *J* = 1.7 Hz, 1H). ^{13}C NMR (101 MHz, MeOD) δ: 155.71, 133.88, 131.98, 130.69, 130.29, 130.19, 129.41, 129.34, 128.32, 123.68, 111.34. MS (EI): m/z (rel. int.) 262.[5]

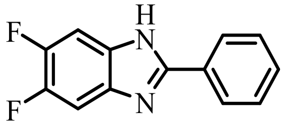

5,6-difluoro-2-phenyl-1*H*-benzo[d]imidazole (4ga):

Yield: 82%. ^{1}H NMR (300 MHz, MeOD) δ: 7.99 – 7.95 (m, 2H), 7.48 – 7.45 (m, 3H), 7.37 (dd, *J* = 17.5, 8.7 Hz, 2H). ^{13}C NMR (75 MHz, MeOD) δ: 155.31, [151.09, 150.87, 147.89, 147.67 (dd, 1,3*J*C-F = 243.6, 16.9 Hz)]], 134.14, 133.94, 131.69, 130.51, 130.19, 129.41, 127.71, [103.54, 103.45, 103.37, 103.26 (dd, 2,3*J*C-F = 15.2, 7.9 Hz)]. MS (EI): m/z (rel. int.) 230.[5]

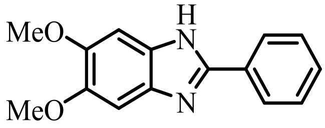

5,6-dimethoxy-2-phenyl-1*H*-benzo[d]imidazole (4ha):

Yield: 59%. ^{1}H NMR (401 MHz, MeOD) δ: 8.00 – 7.97 (m, 2H), 7.49-7.42 (m, 3H), 7.10 (s, 2H), 3.86 (s, 6H). ^{13}C NMR (101 MHz, MeOD) δ: 152.04, 149.03, 134.15, 131.40, 130.92, 130.27, 127.41, 98.99, 57. 04. MS (EI): m/z (rel. int.) 254.[5]

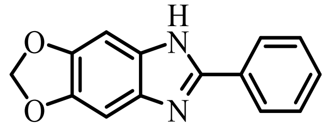

6-phenyl-5*H*-[1,3]dioxolo[4',5':4,5]benzo[1,2-d]imidazole (4ia):

Yield: 87%. ^{1}H NMR (401 MHz, MeOD) δ: 7.96 (dd, J = 8.2, 1.4 Hz, 2H), 7.50 – 7.42 (m, 3H), 6.99 (s, 2H), 5.94 (s, 2H). ^{13}C NMR (100 MHz, MeOD) δ: 151.94, 146.74, 134.88, 131.18, 130.72, 130.09, 127.13, 102.47, 95.64. MS (EI): m/z (rel. int.) 238.[5]

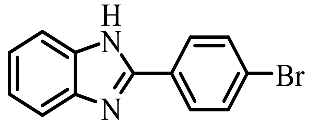

2-(4-bromophenyl)-1*H*-benzo[d]imidazole (4ab):

Yield: 73%. ^{1}H NMR (401 MHz, MeOD) δ: 8.02 (d, J = 8.2 Hz, 2H), 7.73 – 7.71 (m, 2H), 7.62 – 7.60 (m, 2H), 7.28 (dd, J = 6.1, 3.1 Hz, 2H); ^{13}C NMR (101 MHz, MeOD) δ: 152.91, 140.89, 134.05, 130.78, 130.12, 126.13, 124.85, 116.65. MS (EI): m/z (rel. int.) 272.[6]

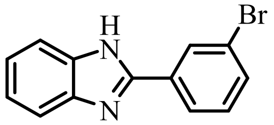

2-(3-bromophenyl)-1*H*-benzo[d]imidazole (4ac):

Yield: 59%. ^{1}H NMR (300 MHz, MeOD) δ: 8.27 (t, J = 1.8 Hz, 1H), 8.06 – 8.02 (m, 1H), 7.63 – 7.58 (m, 3H), 7.44 (t, J = 7.9 Hz, 1H), 7.26 (dd, J = 6.1, 3.2 Hz, 2H). ^{13}C NMR (75 MHz, MeOD) δ: 151.36, 140.30, 133.87, 132.90, 131.69, 130.39, 126.17, 123.97, 123.81, 116.19. MS (EI): m/z (rel. int.) 272.[5]

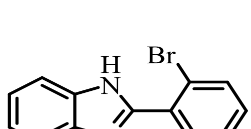

2-(2-bromophenyl)-1*H*-benzo[d]imidazole (4ad):

Yield: 64%. ^{1}H NMR (401 MHz, MeOD) δ: 7.80 (ddd, J = 15.3, 7.8, 1.5 Hz, 2H), 7.74 – 7.71 (m, 2H), 7.55 (td, J = 7.5, 1.3 Hz, 1H), 7.45 (td, J = 7.7, 1.8 Hz, 1H), 7.32 – 7.28 (m, 2H). ^{13}C NMR (101 MHz, MeOD) δ: 152.36, 134.68, 133.53, 133.25, 132.65, 131.69, 130.17, 128.82, 127.83, 124.03, 123.27. MS (EI): m/z (rel. int.) 272.[7]

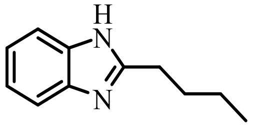

2-butyl-1*H*-benzo[d]imidazole (4ae):

Yield: 86%. ^{1}H NMR (300 MHz, MeOD) δ: 7.49 – 7.46 (m, 2H), 7.19 – 7.16 (m, 2H), 2.89 (t, *J* = 7.7 Hz, 2H), 1.82 (p, *J* = 7.3 Hz, 2H), 1.41 (dd, *J* = 7.2, 3.6 Hz, 2H), 0.97 (t, *J* = 6.7 Hz, 3H). ^{13}C NMR (101 MHz, MeOD) δ: 156.95, 139.47, 123.13, 115.25, 31.44, 29.46, 23.35, 14.04. MS (EI): m/z (rel. int.) 174.[8]

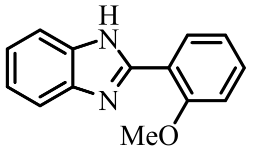

2-(2-methoxyphenyl)-1*H*-benzo[d]imidazole (4af):

Yield: 88%. ^{1}H NMR (401 MHz, MeOD) δ: 8.24 (dd, *J* = 7.8, 1.7 Hz, 1H), 7.63 (dd, *J* = 6.1, 3.2 Hz, 2H), 7.50 (ddd, *J* = 8.6, 7.4, 1.7 Hz, 1H), 7.24 (dd, *J* = 6.1, 3.2 Hz, 2H), 7.22 (d, *J* = 8.3 Hz, 1H), 7.13 (td, *J* = 7.8, 1.0 Hz, 1H), 4.08 (s, 3H). ^{13}C NMR (101 MHz, MeOD) δ: 158.56, 150.87, 139.15, 132.65, 130.79, 123.46, 122.00, 118.76, 115.60, 112.69, 56.07. MS (EI): m/z (rel. int.) 224.[5]

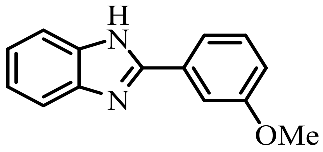

2-(3-methoxyphenyl)-1*H*-benzo[d]imidazole (4ag):

Yield: 89%. ^{1}H NMR (401 MHz, MeOD) δ: 7.65 – 7.55 (m, 4H), 7.40 (t, *J* = 8.0 Hz, 1H), 7.23 (dd, *J* = 6.1, 3.2 Hz, 2H), 7.02 (ddd, *J* = 8.3, 2.5, 0.8 Hz, 1H), 3.86 (s, 3H). ^{13}C NMR (101 MHz, MeOD) δ: 161.73, 153.27, 140.21, 132.20, 131.23, 123.97, 120.01, 117.37, 116.08, 112.92, 55.89. MS (EI): m/z (rel. int.) 224.[5]

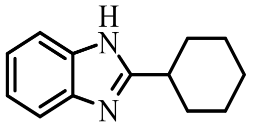

2-cyclohexyl-1*H*-benzo[d]imidazole (4ah):

Yield: 53%. ^{1}H NMR (300 MHz, MeOD) δ: 7.44 (dd, *J* = 6.0, 3.2 Hz, 2H), 7.13 (dd, *J* = 6.0, 3.2 Hz, 2H), 2.85 (tt, *J* = 11.9, 3.5 Hz, 1H), 2.17 – 2.02 (m, 2H), 1.87-1.81 (m, 2H), 1.66 – 1.61 (m, 2H), 1.45 – 1.24 (m, 4H). ^{13}C NMR (75 MHz, MeOD) δ: 160.81, 139.32, 123.11, 115.35, 39.85, 32.85, 27.21, 26.99. MS (EI): m/z (rel. int.) 200.[5]

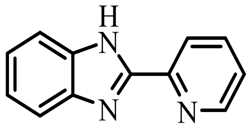

2-(pyridin-2-yl)-1*H*-benzo[d]imidazole (4ai):

Yield: 50%. ^{1}H NMR (300 MHz, MeOD) δ: 8.70 – 8.68 (m, 1H), 8.27 (dd, *J* = 7.0, 0.9 Hz, 1H), 7.93 (td, *J* = 7.8, 1.7 Hz, 1H), 7.63 (dd, *J* = 6.0, 3.2 Hz, 2H), 7.44 (ddd, *J* = 7.6, 4.9, 1.1 Hz, 1H), 7.28 (dd, *J* = 6.1, 3.2 Hz, 2H). ^{13}C NMR (75 MHz, MeOD) δ: 152.44, 150.87, 149.43, 138.51, 125.92, 124.37, 122.52. MS (EI): m/z (rel. int.) 195.[5]

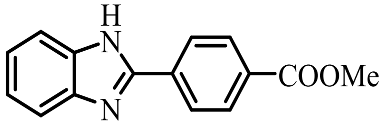

Methyl 4-(1*H*-benzo[d]imidazol-2-yl)benzoate (4aj):

Yield: 52%. ^{1}H NMR (300 MHz, MeOD) δ: 8.05 – 7.98 (m, 4H), 7.53 (dd, *J* = 5.9, 3.2 Hz, 2H), 7.19 (dd, *J* = 6.1, 3.1 Hz, 2H), 3.82 (s, 3H). ^{13}C NMR (75 MHz, MeOD) δ: 167.80, 151.82, 135.02, 132.33, 131.09, 130.74, 127.64, 124.32, 116.17, 52.82. MS (EI): m/z (rel. int.) 252.[5]

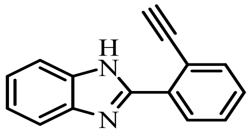

2-(2-ethynylphenyl)-1*H*-benzo[d]imidazole (4ak):

Yield: 84%. ^{1}H NMR (300 MHz, MeOD) δ: 8.62 – 8.59 (m, 1H), 8.42 (d, *J* = 7.3 Hz, 1H), 8.00 (d, *J* = 8.1 Hz, 1H), 7.85 – 7.78 (m, 1H), 7.69 – 7.65 (m, 1H), 7.49 (t, *J* = 3.4 Hz, 1H), 7.38 (t, *J* = 7.6 Hz, 1H), 7.16 (d, *J* = 7.3 Hz, 1H). ^{13}C NMR (75 MHz, CDCl3) δ: 148.06, 143.87, 133.35, 131.49, 131.21, 129.30, 128.49, 126.09, 125.41, 123.76, 123.37, 123.02, 119.58, 112.86, 111.71. MS (EI): m/z (rel. int.) 218.[5]

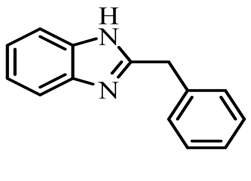

2-benzyl-1*H*-benzo[d]imidazole (4al):

Yield: 51%. ^{1}H NMR (300 MHz, MeOD) δ: 7.42 (dd, *J* = 6.0, 3.2 Hz, 2H), 7.22 (d, *J* = 4.4 Hz, 5H), 7.10 (dd, *J* = 6.0, 3.2 Hz, 2H), 4.14 (s, 2H). ^{13}C NMR (75 MHz, MeOD) δ: 155.20, 139.61, 138.24, 129.78, 129,74, 127.97, 123.35, 115.49, 36.07. MS (EI): m/z (rel. int.) 208. [9]

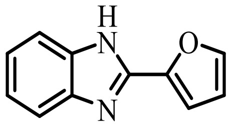

2-(furan-2-yl)-1*H*-benzo[d]imidazole (4am):

Yield: 93%. ^{1}H NMR (401 MHz, MeOD) δ 7.58 – 7.57 (m, 1H), 7.42 (dd, *J* = 6.1, 3.1 Hz, 2H), 7.09 (dd, *J* = 6.0, 3.2 Hz, 2H), 7.04 (d, *J* = 3.5 Hz, 1H), 6.49 (dd, *J* = 3.5, 1.8 Hz, 1H). ^{13}C NMR (100 MHz, MeOD) δ: 146.45, 145.73, 145.37, 124.04, 115.83, 115.85, 113.19, 111.85. MS (EI): m/z (rel. int.) 184.[9]

^{1}H NMR, ^{13}C NMR AND ^{19}F NMR SPECTRA OF THE ISOLATED BENZIMIDAZOLES

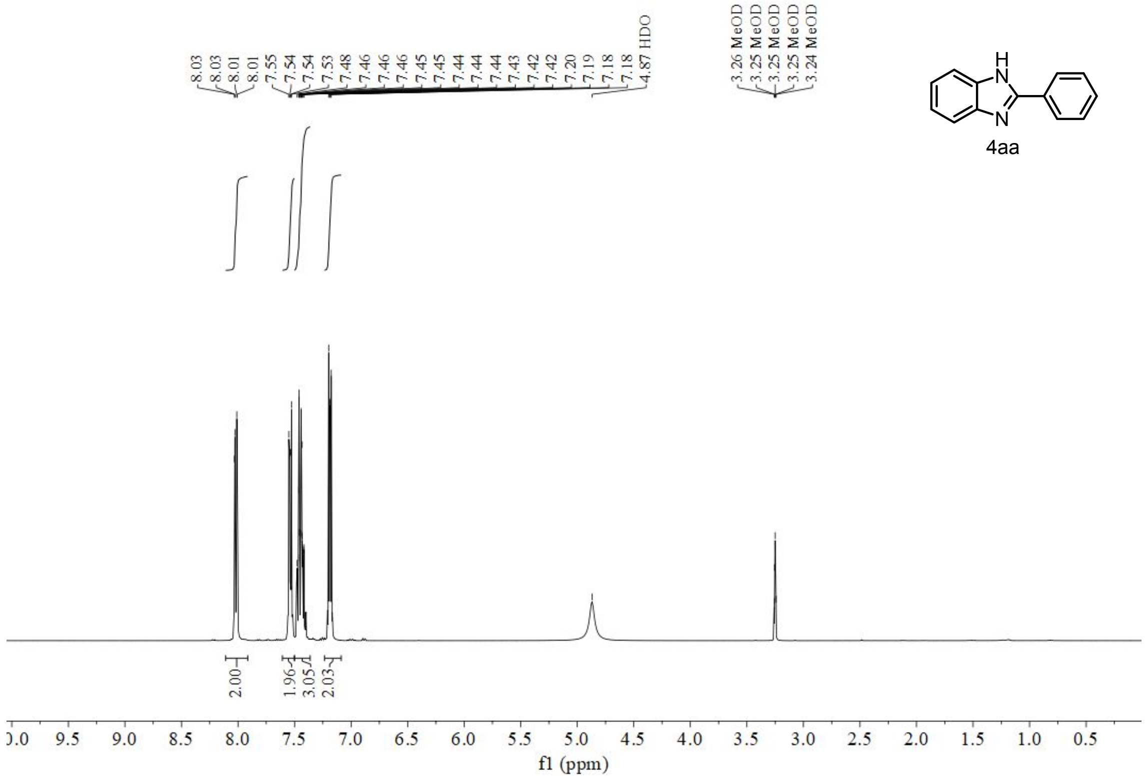

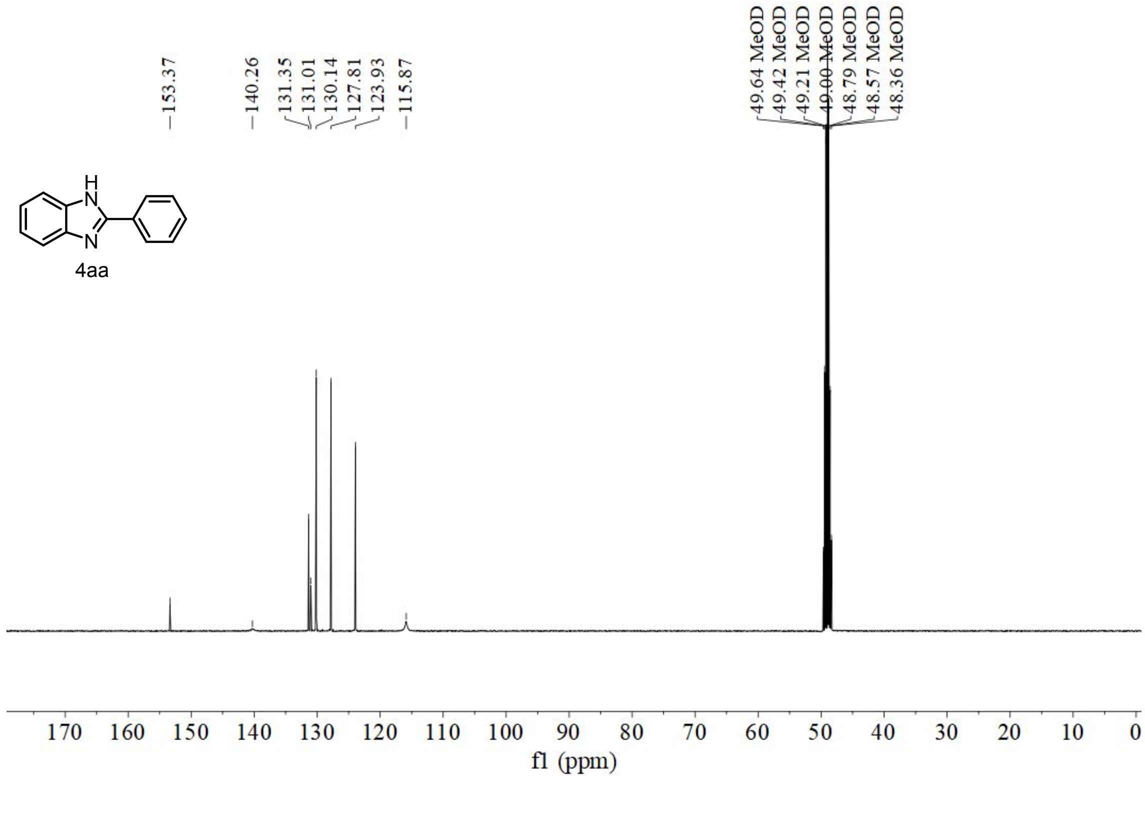

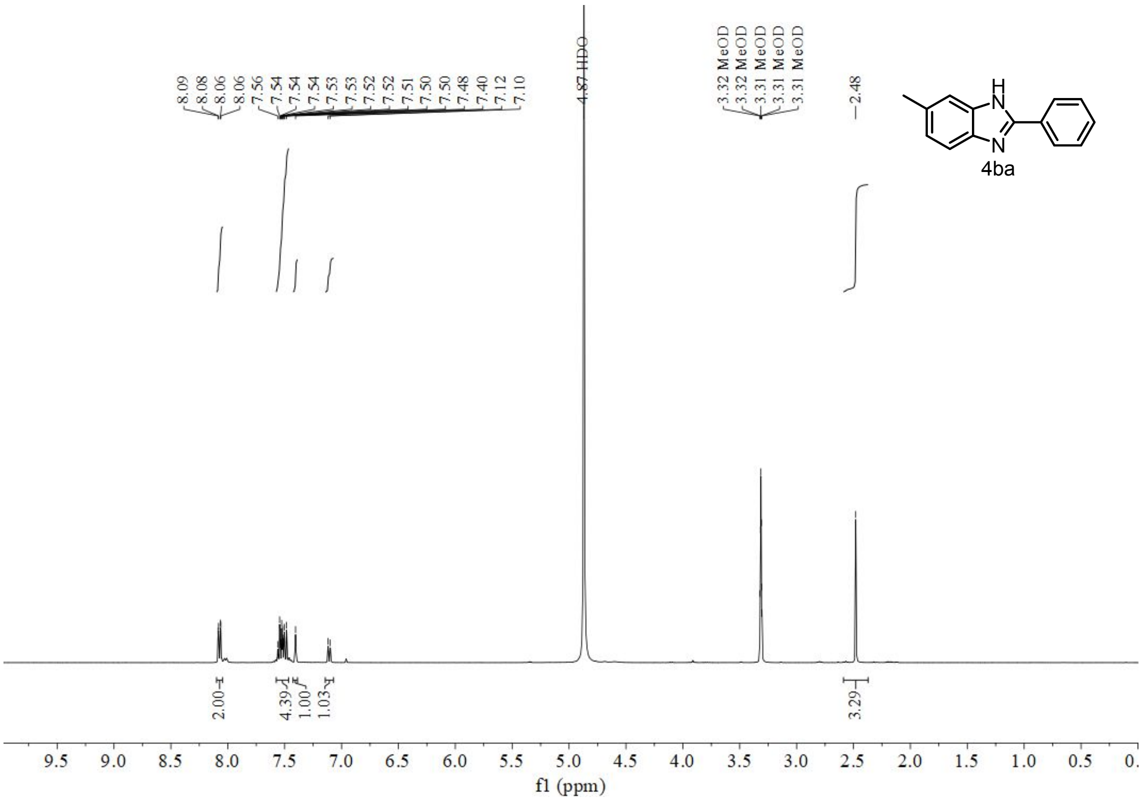

8.09
8.08
8.06
8.06
7.56
7.54
7.54
7.54
7.53
7.53
7.52
7.52
7.51
7.50
7.50
7.48
7.40
7.12
7.10
4.87 HDO
3.32 MeOD
3.32 MeOD
3.31 MeOD
3.31 MeOD
3.31 MeOD
2.48
4ba
2.00
4.39
1.00
1.03
3.29
f1 (ppm)

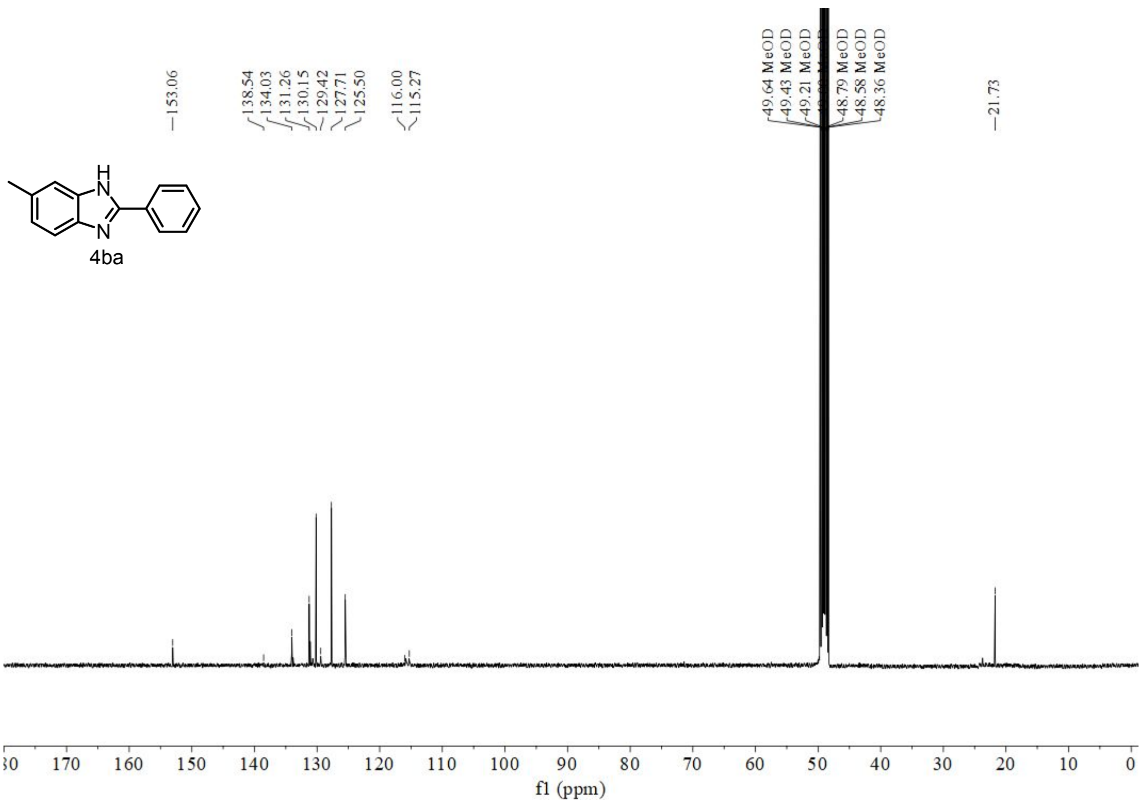

153.06
138.54
134.03
131.26
130.15
129.42
127.71
125.50
116.00
115.27
49.64 MeOD
49.43 MeOD
49.21 MeOD
48.79 MeOD
48.58 MeOD
48.36 MeOD
21.73
4ba
f1 (ppm)

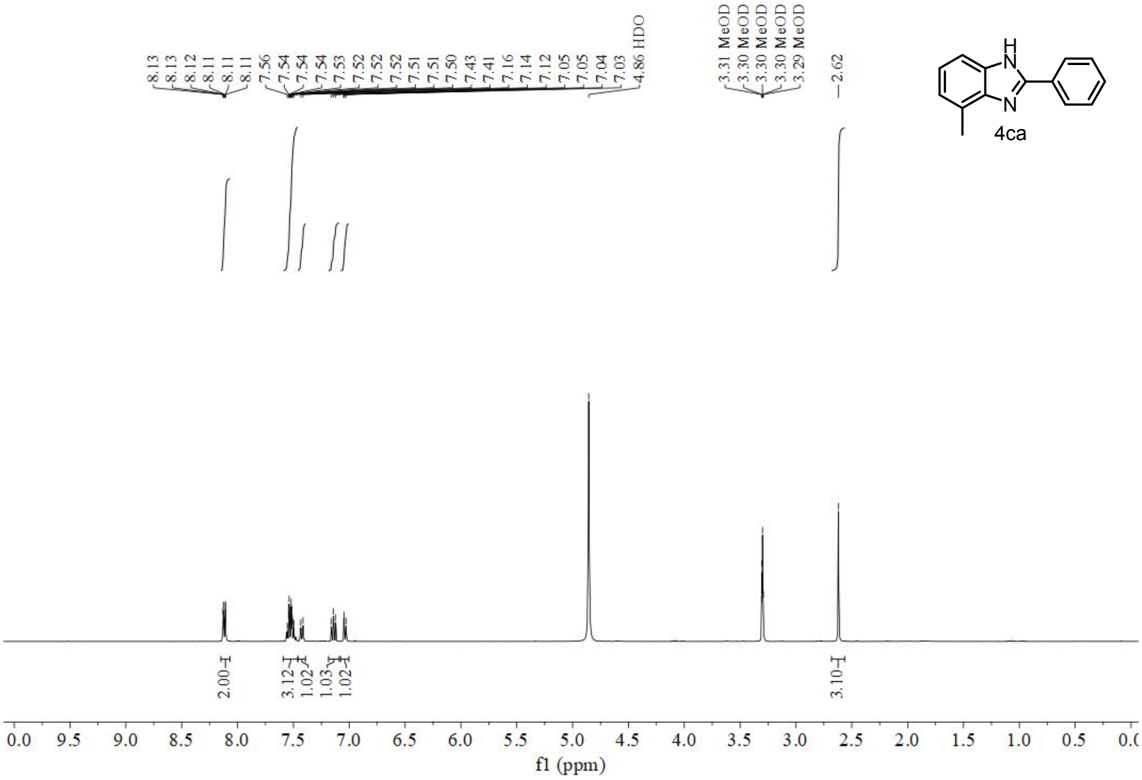
8.13
8.13
8.12
8.11
8.11
8.11
7.56
7.54
7.54
7.54
7.53
7.52
7.52
7.52
7.51
7.51
7.50
7.43
7.41
7.16
7.14
7.12
7.05
7.05
7.04
7.03
4.86 HDO
3.31 MeOD
3.30 MeOD
3.30 MeOD
3.30 MeOD
3.29 MeOD
2.62
H
N
N
4ca
2.00
3.12
1.02
1.03
1.02
3.10
f1 (ppm)

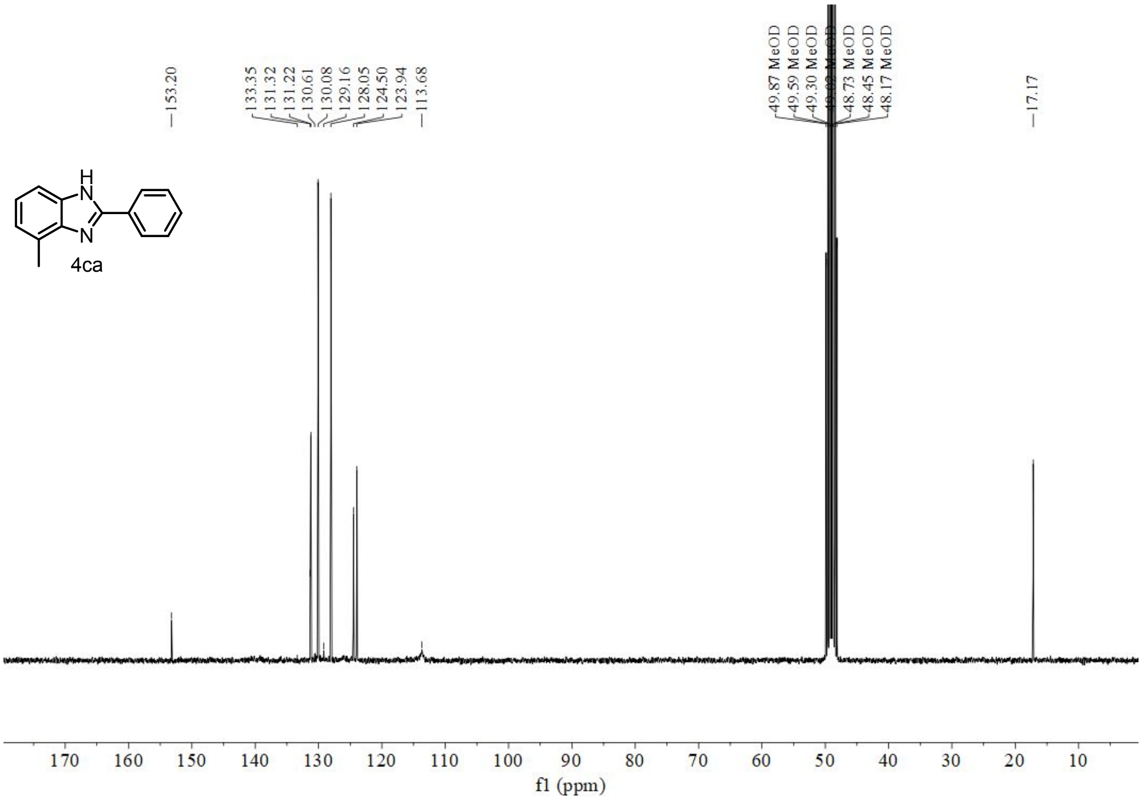
153.20
133.35
131.32
131.22
130.61
130.08
129.16
128.05
124.50
123.94
113.68
49.87 MeOD
49.59 MeOD
49.30 MeOD
48.73 MeOD
48.45 MeOD
48.17 MeOD
17.17
H
N
N
4ca
f1 (ppm)

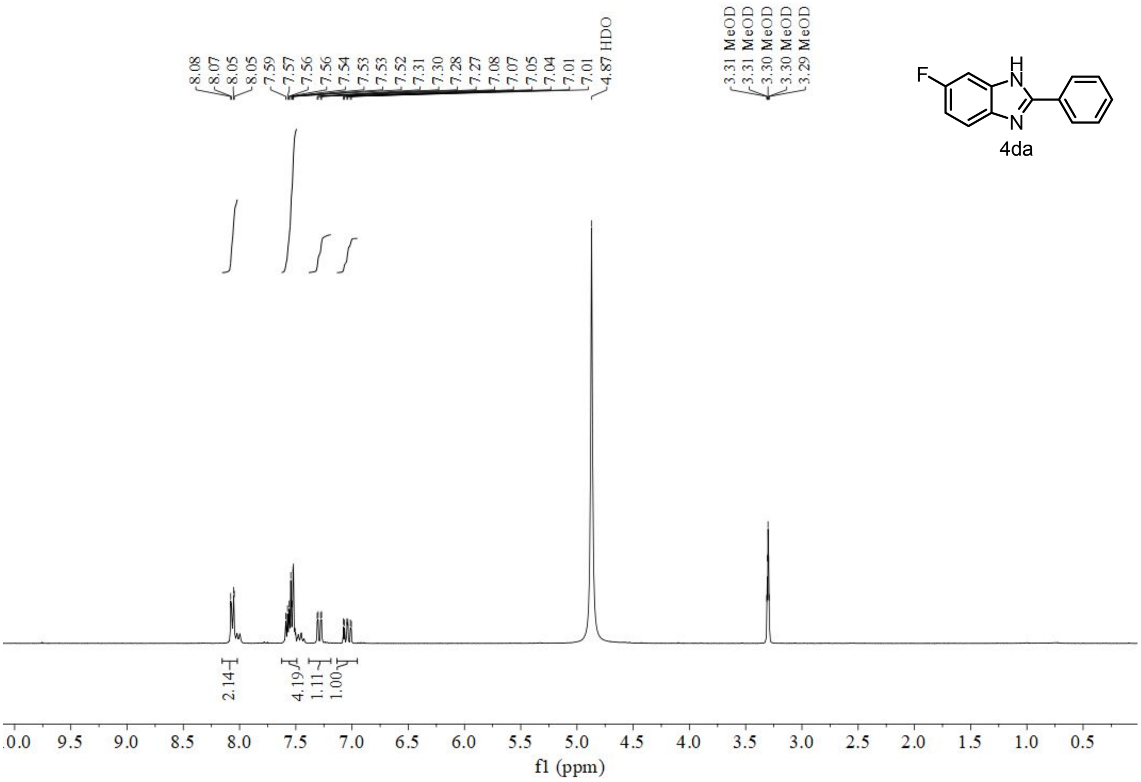
8.08
8.07
8.05
8.05
7.59
7.57
7.56
7.56
7.54
7.53
7.53
7.52
7.31
7.30
7.28
7.27
7.08
7.07
7.05
7.04
7.01
7.01
4.87 HDO
3.31 MeOD
3.31 MeOD
3.30 MeOD
3.30 MeOD
3.29 MeOD
F
H
N
N
4da
2.14
4.19
1.11
1.00
.0.0 9.5 9.0 8.5 8.0 7.5 7.0 6.5 6.0 5.5 5.0 4.5 4.0 3.5 3.0 2.5 2.0 1.5 1.0 0.5
f1 (ppm)

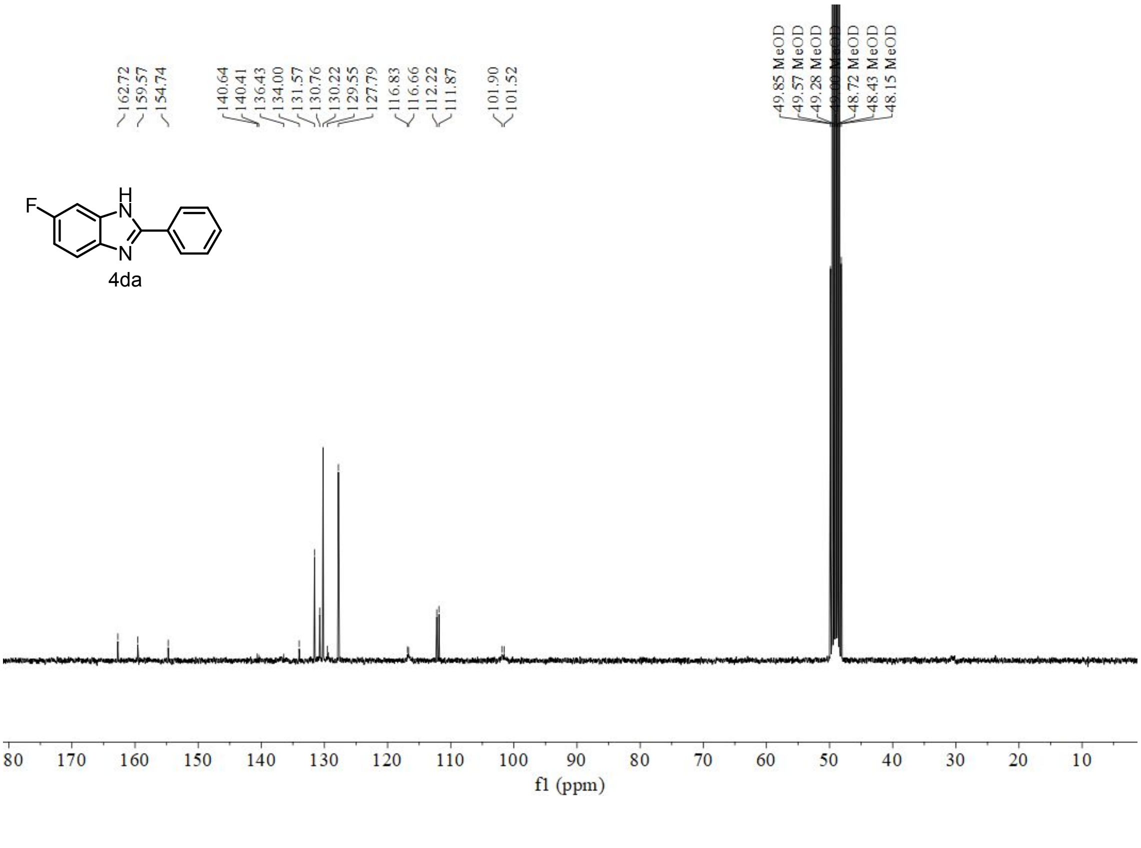
162.72
159.57
154.74
140.64
140.41
136.43
134.00
131.57
130.76
130.22
129.55
127.79
116.83
116.66
112.22
111.87
101.90
101.52
49.85 MeOD
49.57 MeOD
49.28 MeOD
48.72 MeOD
48.43 MeOD
48.15 MeOD
F
H
N
N
4da
80 170 160 150 140 130 120 110 100 90 80 70 60 50 40 30 20 10
f1 (ppm)

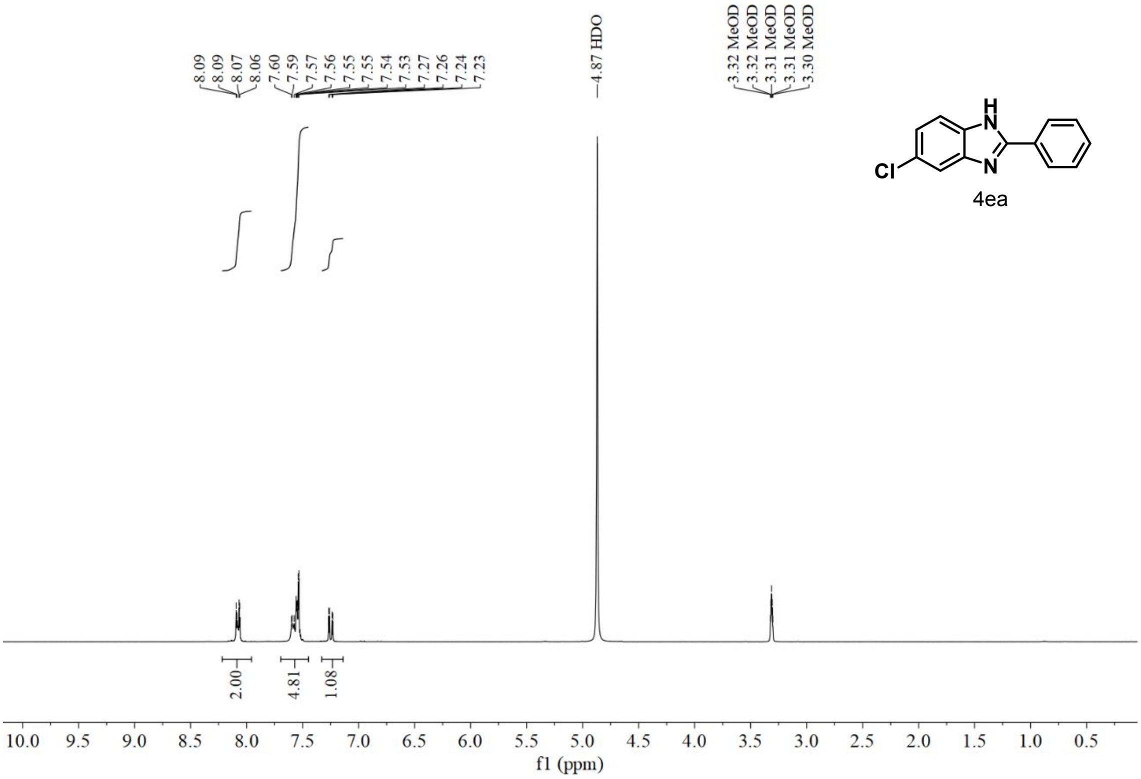
8.09
8.09
8.07
8.06
7.60
7.59
7.57
7.56
7.55
7.55
7.54
7.53
7.27
7.26
7.24
7.23
4.87 HDO
3.32 MeOD
3.32 MeOD
3.31 MeOD
3.31 MeOD
3.30 MeOD
H
N
N
Cl
4ea
2.00
4.81
1.08
10.0 9.5 9.0 8.5 8.0 7.5 7.0 6.5 6.0 5.5 5.0 4.5 4.0 3.5 3.0 2.5 2.0 1.5 1.0 0.5
f1 (ppm)

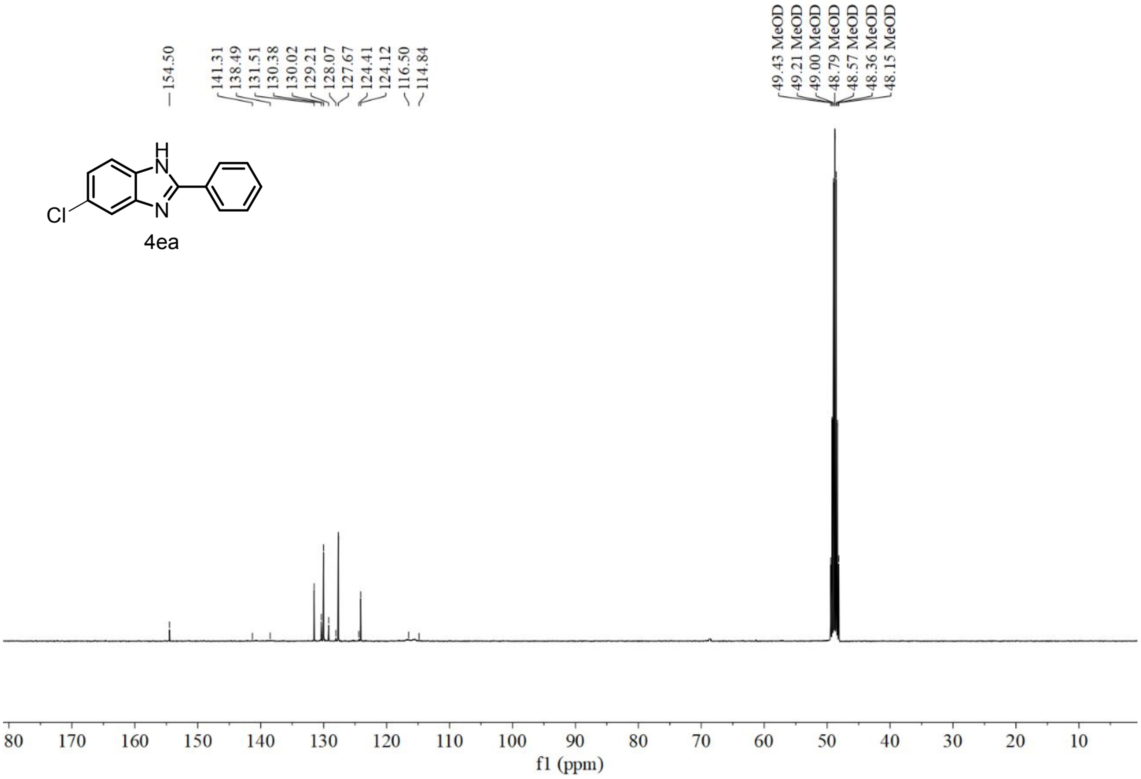
154.50
141.31
138.49
131.51
130.38
130.02
129.21
128.07
127.67
124.41
124.12
116.50
114.84
49.43 MeOD
49.21 MeOD
49.00 MeOD
48.79 MeOD
48.57 MeOD
48.36 MeOD
48.15 MeOD
H
N
N
Cl
4ea
80 170 160 150 140 130 120 110 100 90 80 70 60 50 40 30 20 10
f1 (ppm)

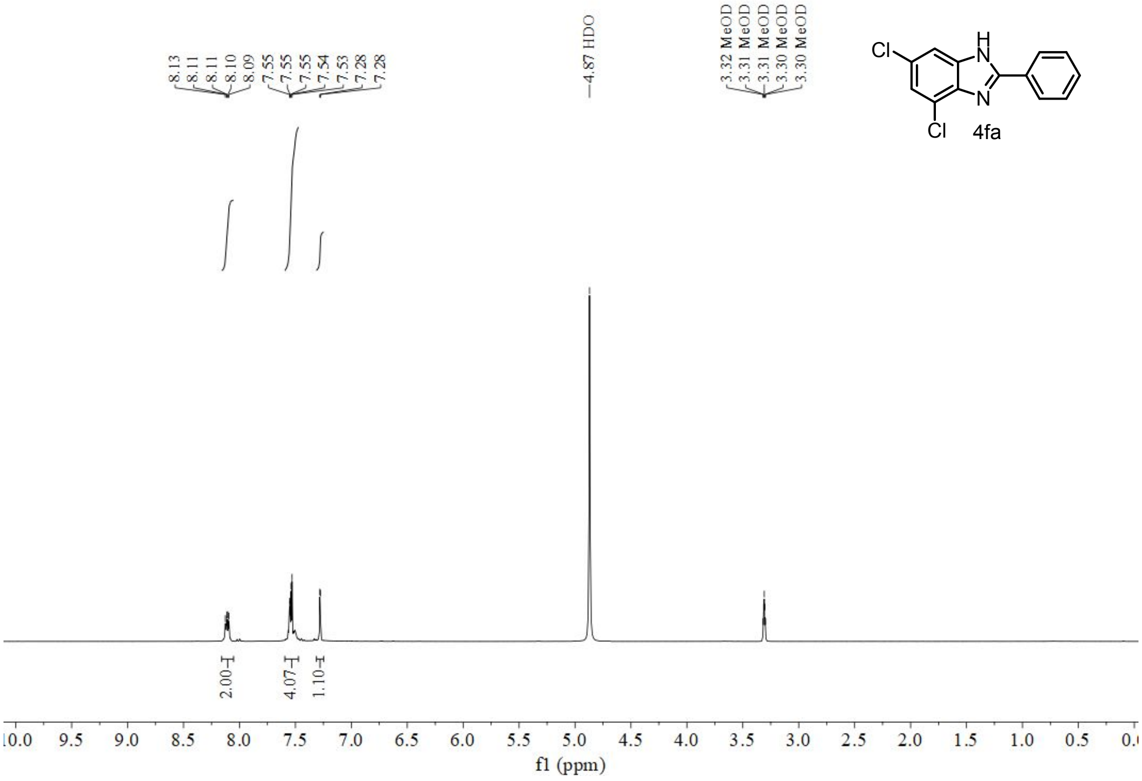
8.13
8.11
8.11
8.10
8.09
7.55
7.55
7.55
7.54
7.53
7.28
7.28
4.87 HDO
3.32 MeOD
3.31 MeOD
3.31 MeOD
3.30 MeOD
3.30 MeOD
Cl
H
N
N
Cl
4fa
2.00
4.07
1.10
10.0 9.5 9.0 8.5 8.0 7.5 7.0 6.5 6.0 5.5 5.0 4.5 4.0 3.5 3.0 2.5 2.0 1.5 1.0 0.5
f1 (ppm)

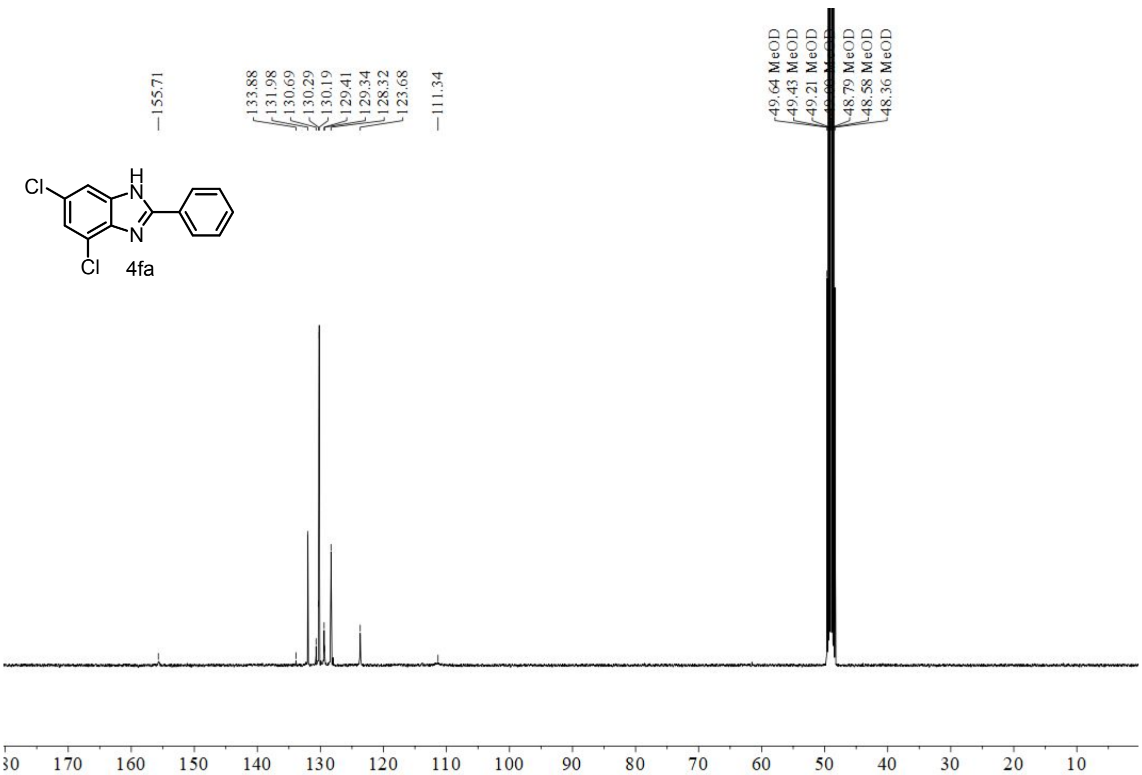
155.71
133.88
131.98
130.69
130.29
130.19
129.41
129.34
128.32
123.68
111.34
49.64 MeOD
49.43 MeOD
49.21 MeOD
48.79 MeOD
48.58 MeOD
48.36 MeOD
Cl
H
N
N
Cl
4fa
170 160 150 140 130 120 110 100 90 80 70 60 50 40 30 20 10
f1 (ppm)

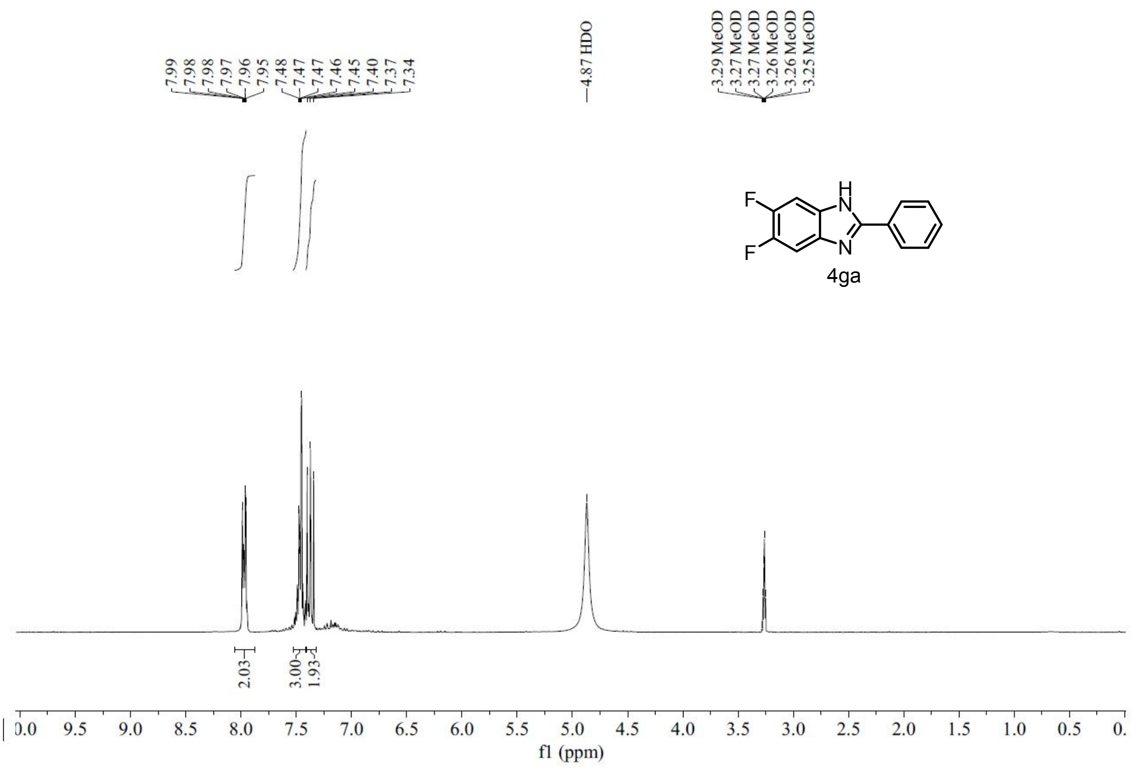
7.99
7.98
7.98
7.97
7.96
7.95
7.48
7.47
7.47
7.46
7.45
7.40
7.37
7.34
4.87 HDO
3.29 MeOD
3.27 MeOD
3.27 MeOD
3.26 MeOD
3.26 MeOD
3.25 MeOD
F
F
H
N
N
4ga
2.03
3.00
1.93
f1 (ppm)

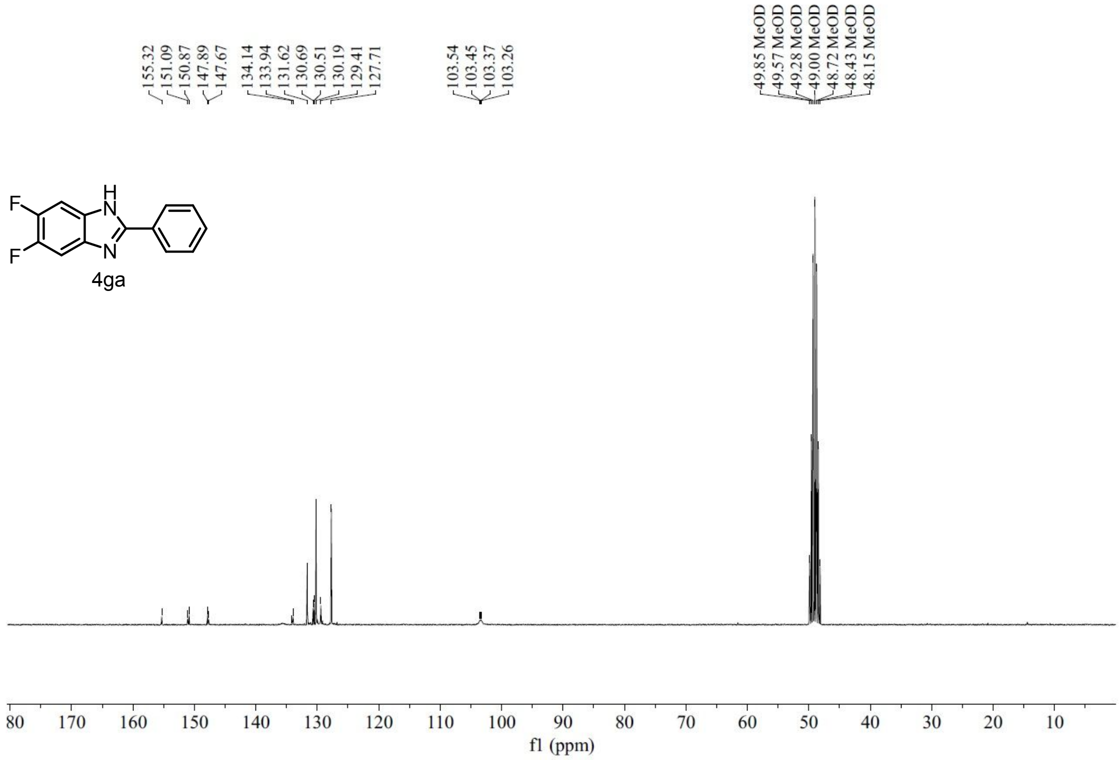
155.32
151.09
150.87
147.89
147.67
134.14
133.94
131.62
130.69
130.51
130.19
129.41
127.71
103.54
103.45
103.37
103.26
49.85 MeOD
49.57 MeOD
49.28 MeOD
49.00 MeOD
48.72 MeOD
48.43 MeOD
48.15 MeOD
F
F
H
N
N
4ga
f1 (ppm)

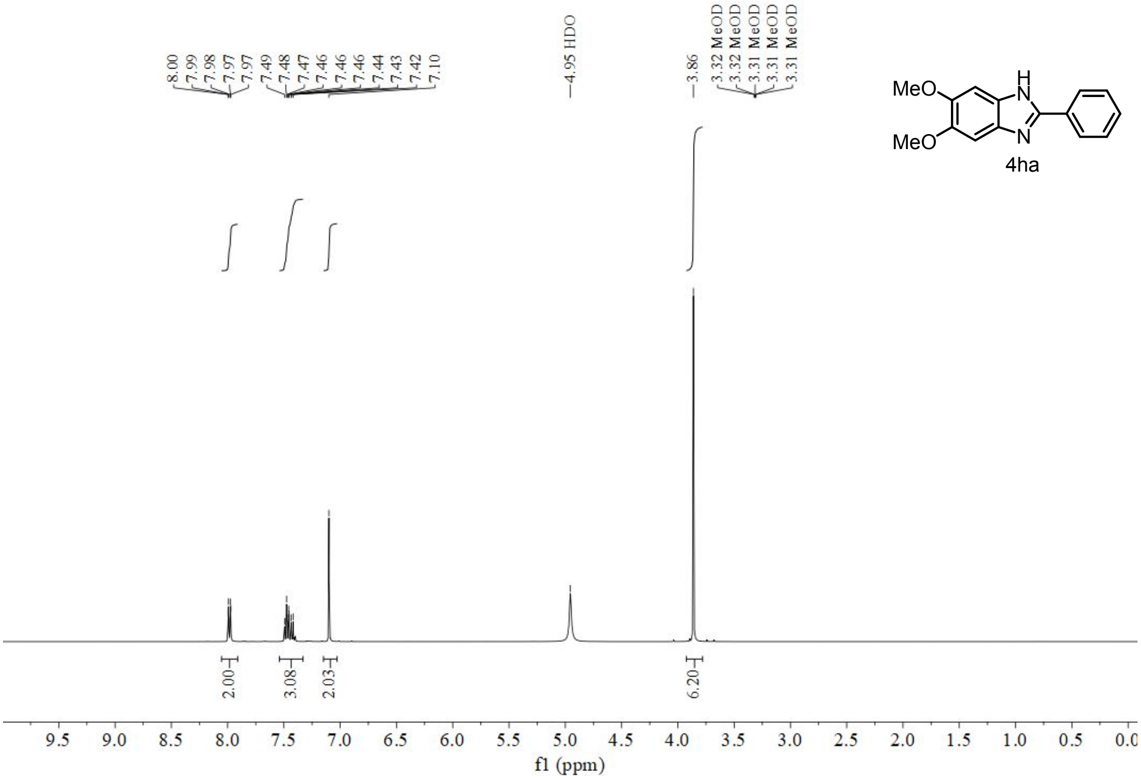
8.00
7.99
7.98
7.97
7.97
7.49
7.48
7.47
7.46
7.46
7.44
7.43
7.42
7.10
4.95 HDO
3.86
3.32 MeOD
3.32 MeOD
3.31 MeOD
3.31 MeOD
3.31 MeOD
MeO
MeO
H
N
N
4ha
2.00
3.08
2.03
6.20
9.5 9.0 8.5 8.0 7.5 7.0 6.5 6.0 5.5 5.0 4.5 4.0 3.5 3.0 2.5 2.0 1.5 1.0 0.5 0.0
f1 (ppm)

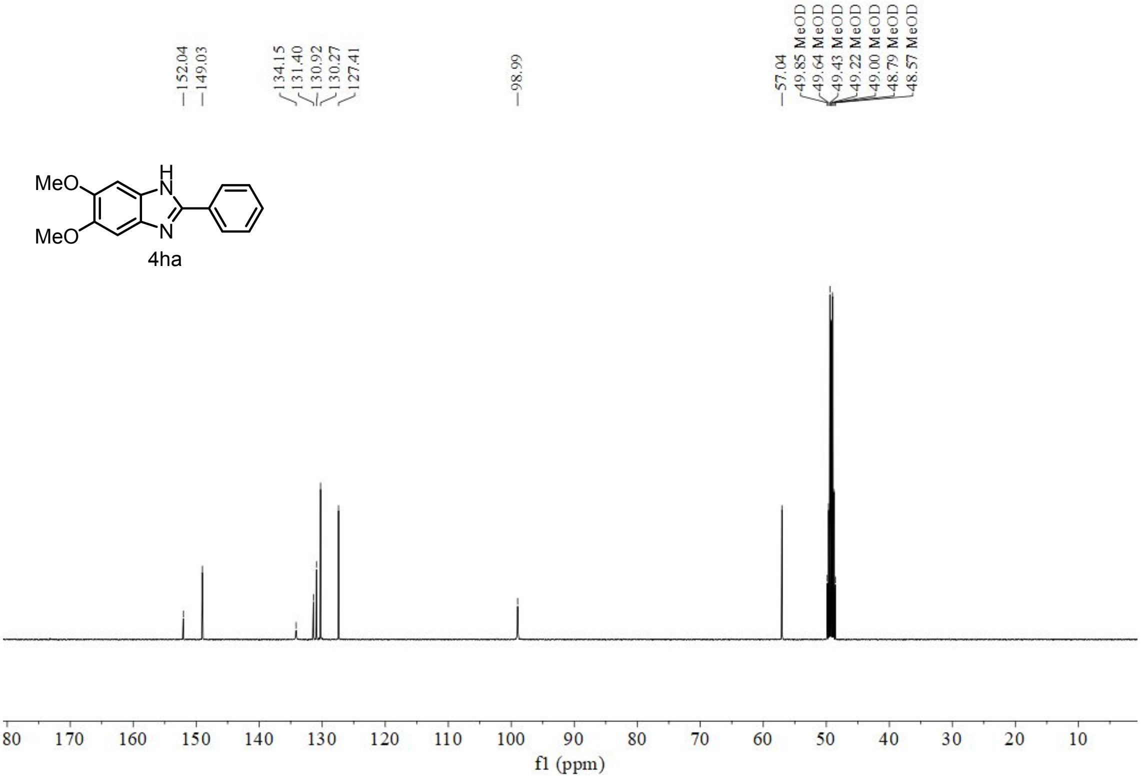
152.04
149.03
134.15
131.40
130.92
130.27
127.41
98.99
57.04
49.85 MeOD
49.64 MeOD
49.43 MeOD
49.22 MeOD
49.00 MeOD
48.79 MeOD
48.57 MeOD
MeO
MeO
H
N
N
4ha
80 170 160 150 140 130 120 110 100 90 80 70 60 50 40 30 20 10
f1 (ppm)

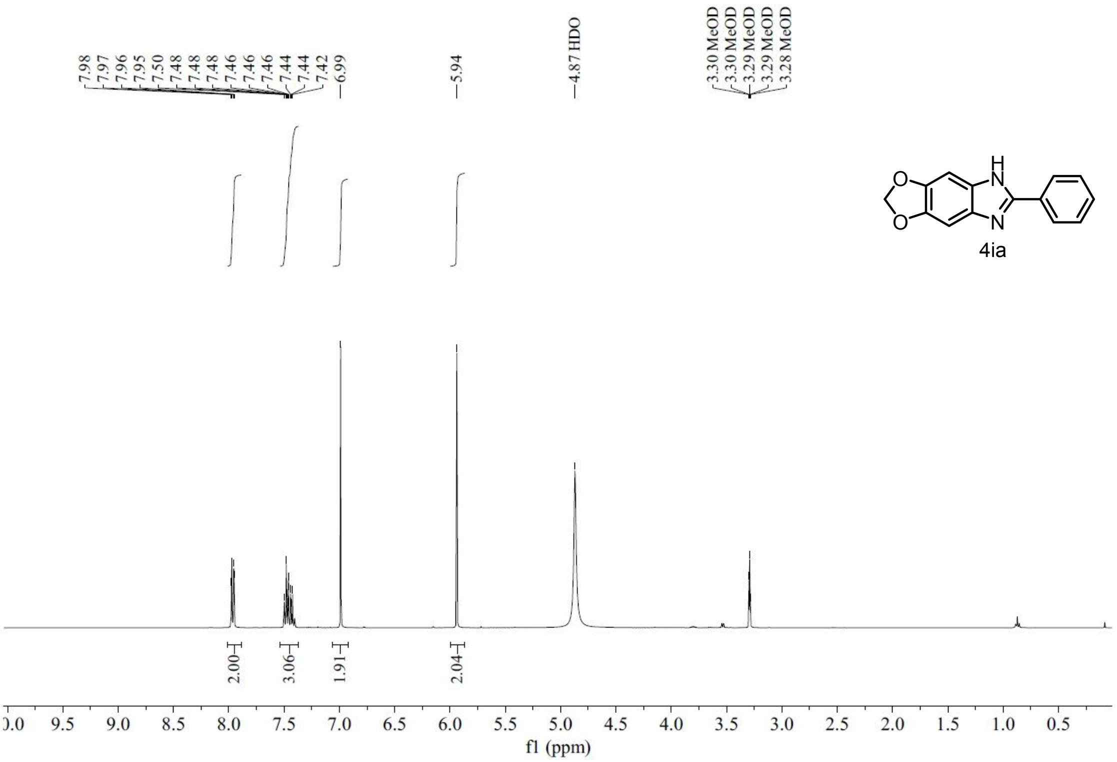
7.98
7.97
7.96
7.95
7.50
7.48
7.48
7.48
7.46
7.46
7.46
7.44
7.44
7.42
6.99
5.94
4.87 HDO
3.30 MeOD
3.30 MeOD
3.29 MeOD
3.29 MeOD
3.28 MeOD
H
N
O
O
N
4ia
2.00
3.06
1.91
2.04
f1 (ppm)

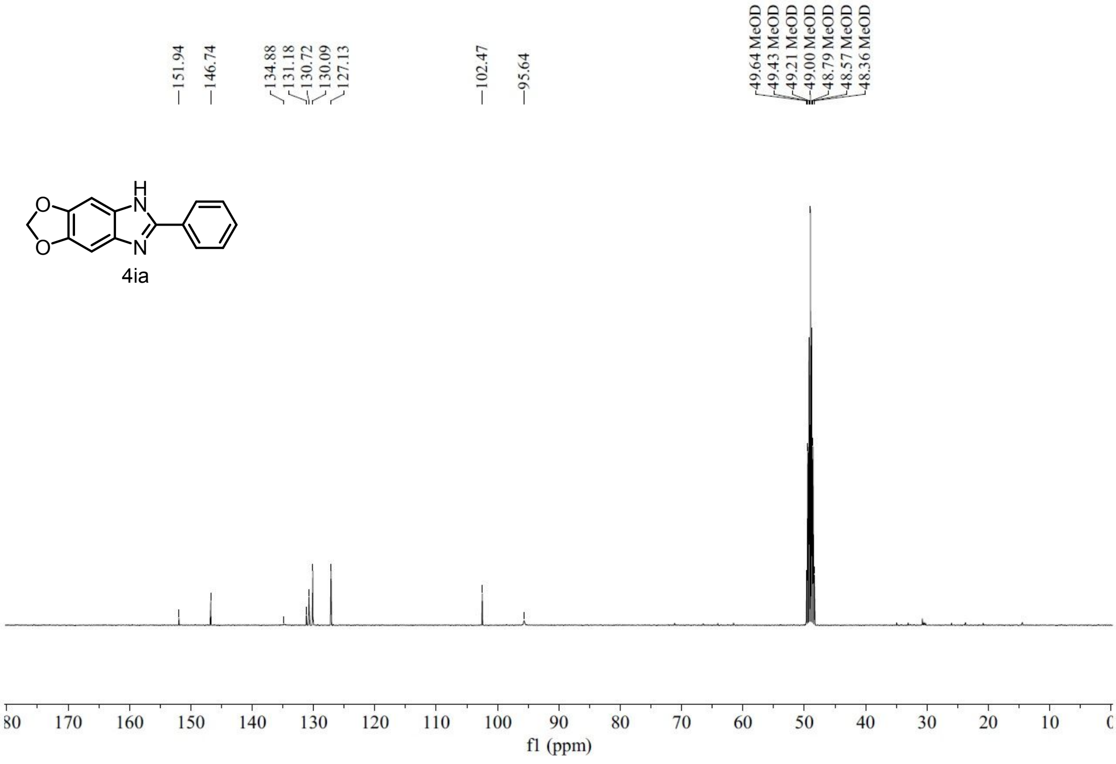
151.94
146.74
134.88
131.18
130.72
130.09
127.13
102.47
95.64
49.64 MeOD
49.43 MeOD
49.21 MeOD
49.00 MeOD
48.79 MeOD
48.57 MeOD
48.36 MeOD
H
N
O
O
N
4ia
f1 (ppm)

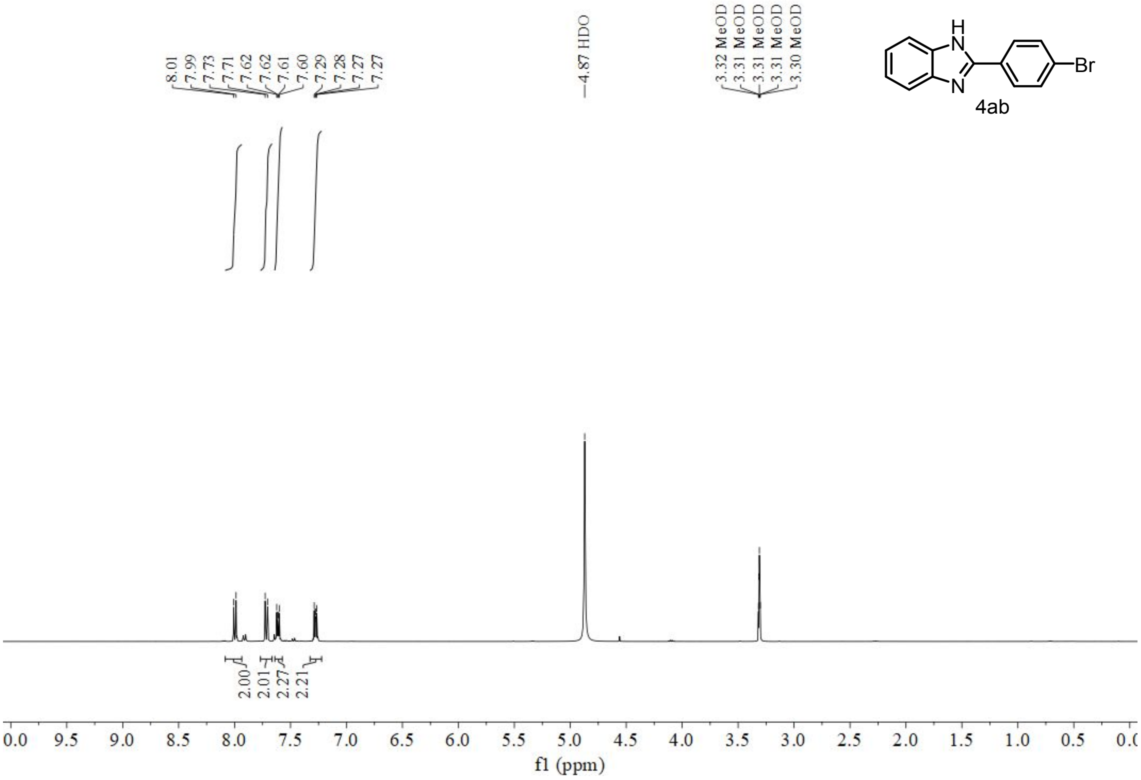
8.01
7.99
7.73
7.71
7.62
7.62
7.61
7.60
7.29
7.28
7.27
7.27
4.87 HDO
3.32 MeOD
3.31 MeOD
3.31 MeOD
3.31 MeOD
3.30 MeOD
H
N
N
Br
4ab
2.00
2.01
2.27
2.21
10.0 9.5 9.0 8.5 8.0 7.5 7.0 6.5 6.0 5.5 5.0 4.5 4.0 3.5 3.0 2.5 2.0 1.5 1.0 0.5 0.0
f1 (ppm)

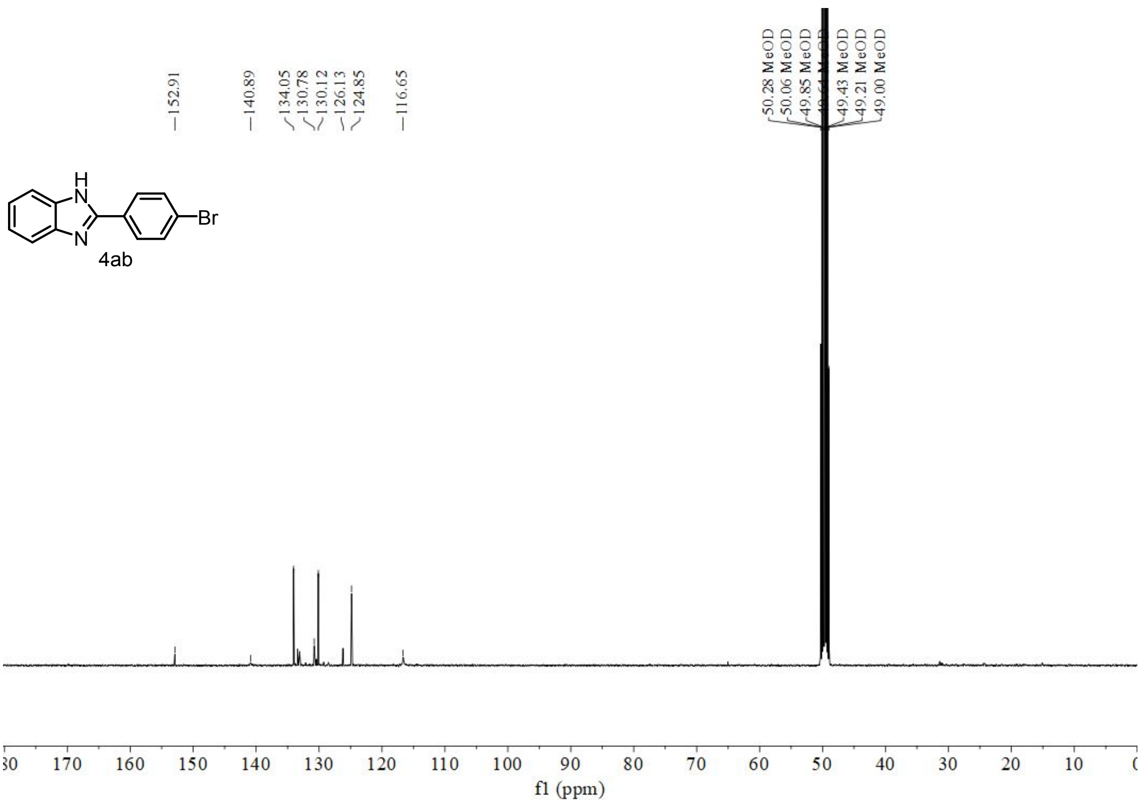
152.91
140.89
134.05
130.78
130.12
126.13
124.85
116.65
50.28 MeOD
50.06 MeOD
49.85 MeOD
49.43 MeOD
49.21 MeOD
49.00 MeOD
H
N
N
Br
4ab
170 160 150 140 130 120 110 100 90 80 70 60 50 40 30 20 10
f1 (ppm)

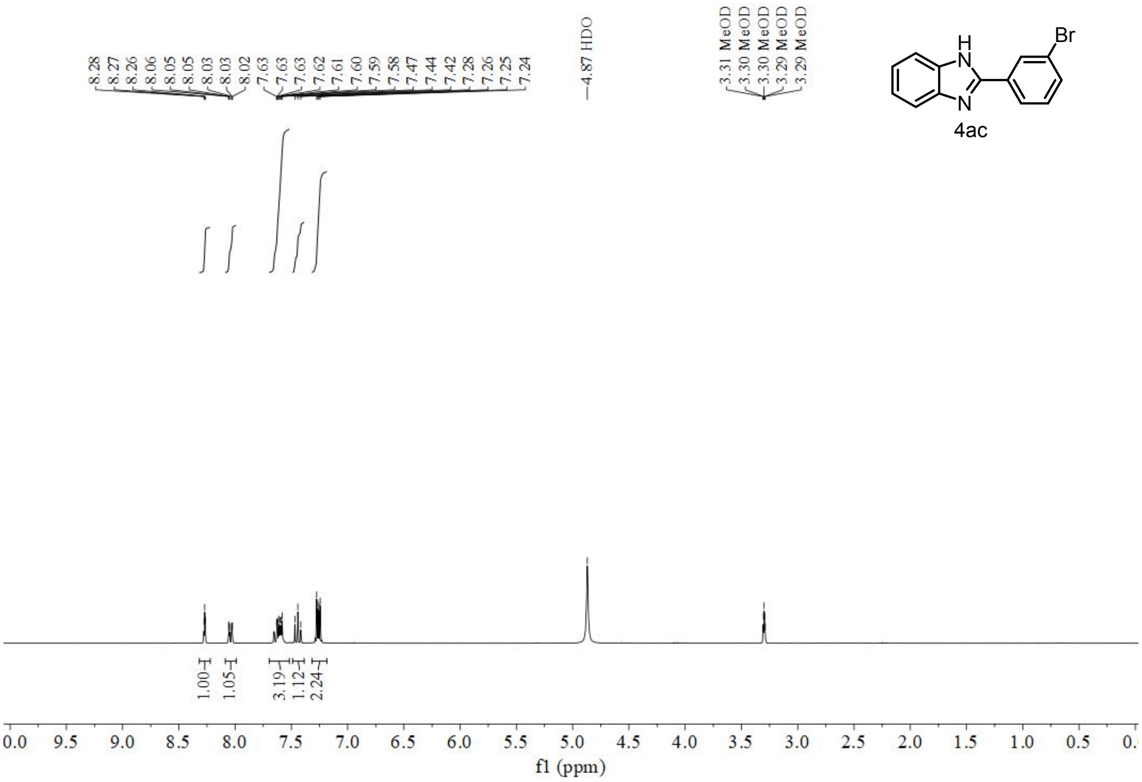
8.28
8.27
8.26
8.06
8.05
8.05
8.03
8.03
8.02
7.63
7.63
7.63
7.62
7.61
7.60
7.59
7.58
7.47
7.44
7.42
7.28
7.26
7.25
7.24
4.87 HDO
3.31 MeOD
3.30 MeOD
3.30 MeOD
3.29 MeOD
3.29 MeOD
H
N
N
Br
4ac
1.00
1.05
3.19
1.12
2.24
f1 (ppm)

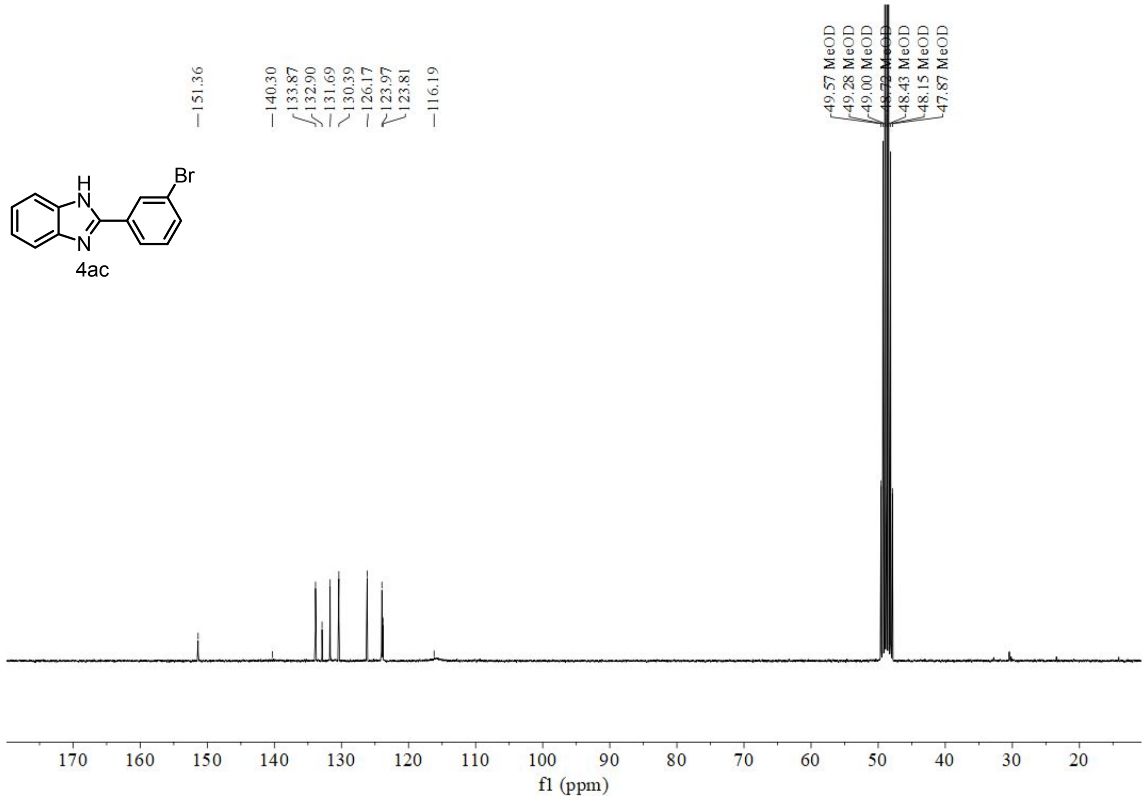
151.36
140.30
133.87
132.90
131.69
130.39
126.17
123.97
123.81
116.19
49.57 MeOD
49.28 MeOD
49.00 MeOD
48.43 MeOD
48.15 MeOD
47.87 MeOD
H
N
N
Br
4ac
f1 (ppm)

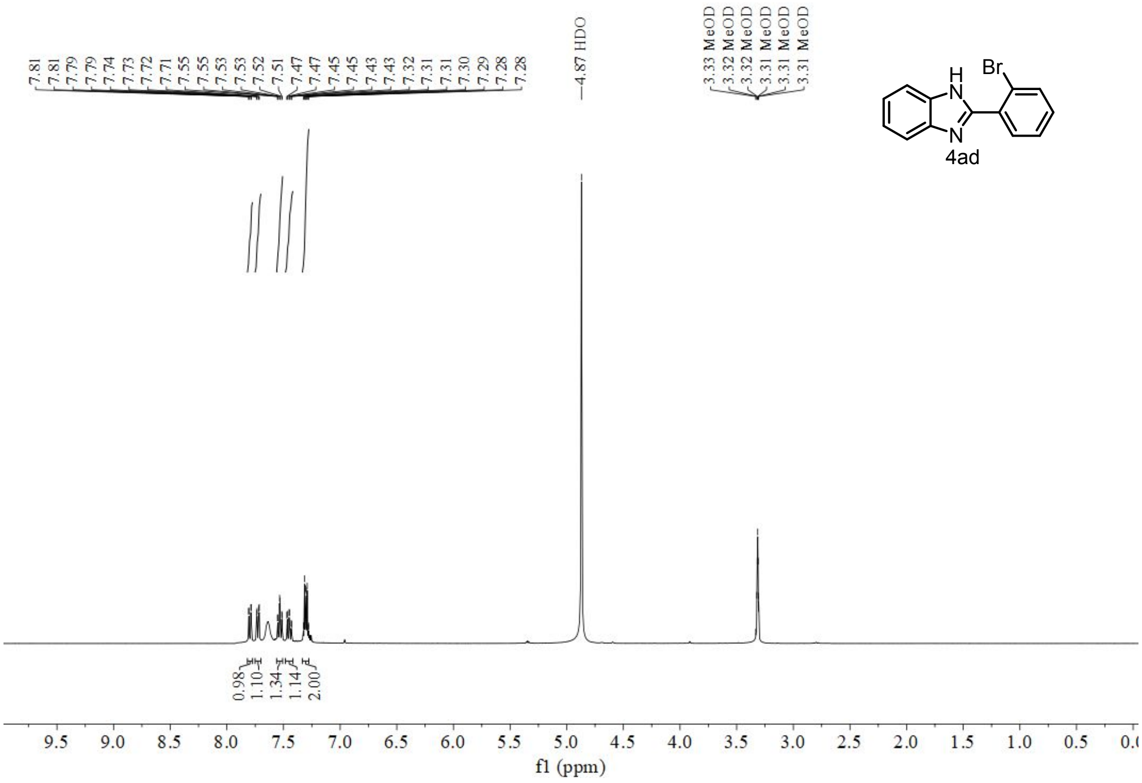
7.81
7.81
7.79
7.79
7.74
7.73
7.72
7.71
7.55
7.55
7.53
7.53
7.52
7.51
7.47
7.47
7.45
7.45
7.43
7.43
7.32
7.31
7.31
7.30
7.29
7.28
7.28
4.87 HDO
3.33 MeOD
3.32 MeOD
3.32 MeOD
3.31 MeOD
3.31 MeOD
3.31 MeOD
Br
H
N
N
4ad
0.98
1.10
1.34
1.14
2.00
f1 (ppm)

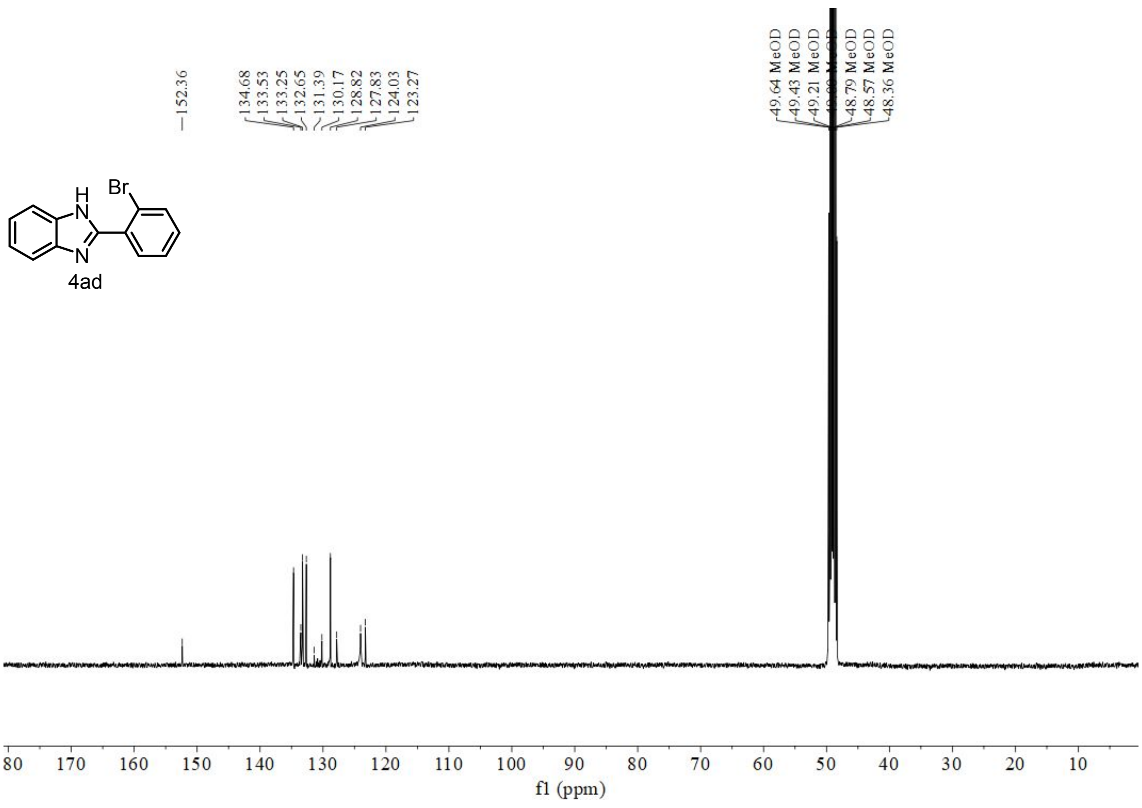
152.36
134.68
133.53
133.25
132.65
131.39
130.17
128.82
127.83
124.03
123.27
49.64 MeOD
49.43 MeOD
49.21 MeOD
48.79 MeOD
48.57 MeOD
48.36 MeOD
Br
H
N
N
4ad
f1 (ppm)

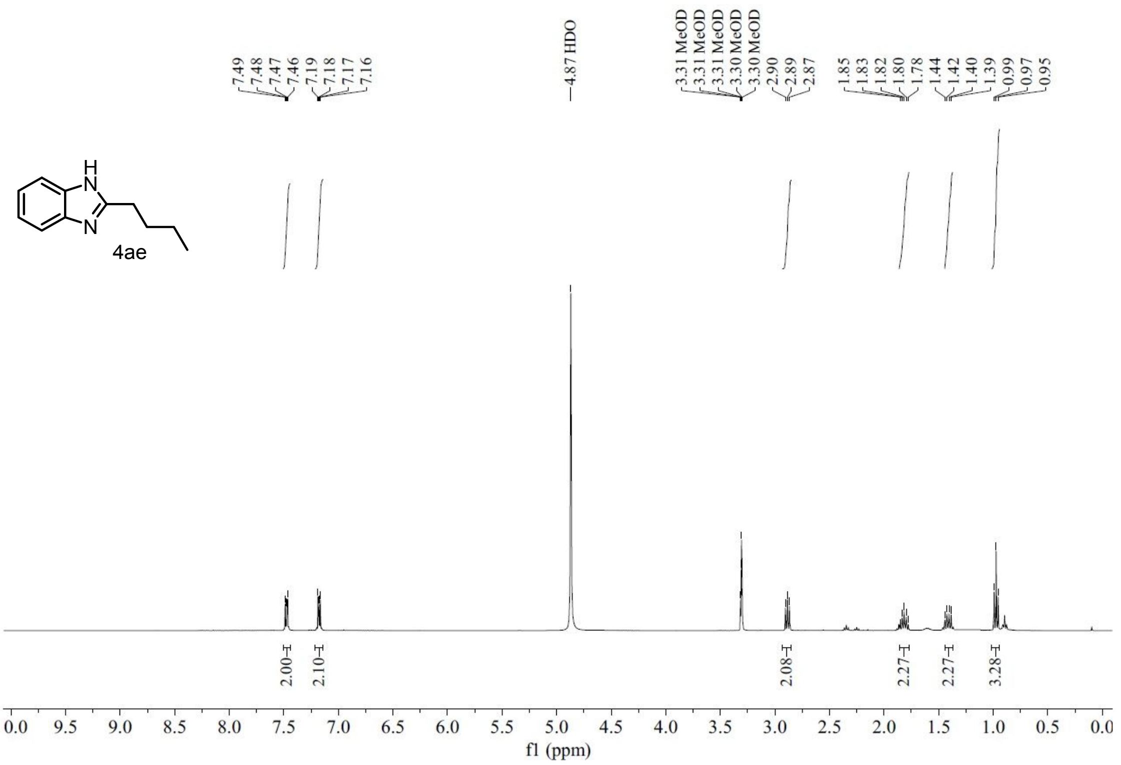
7.49
7.48
7.47
7.46
7.19
7.18
7.17
7.16
4.87 HDO
3.31 MeOD
3.31 MeOD
3.31 MeOD
3.30 MeOD
3.30 MeOD
2.90
2.89
2.87
1.85
1.83
1.82
1.80
1.78
1.44
1.42
1.40
1.39
0.99
0.97
0.95
4ae
2.00
2.10
2.08
2.27
2.27
3.28
f1 (ppm)

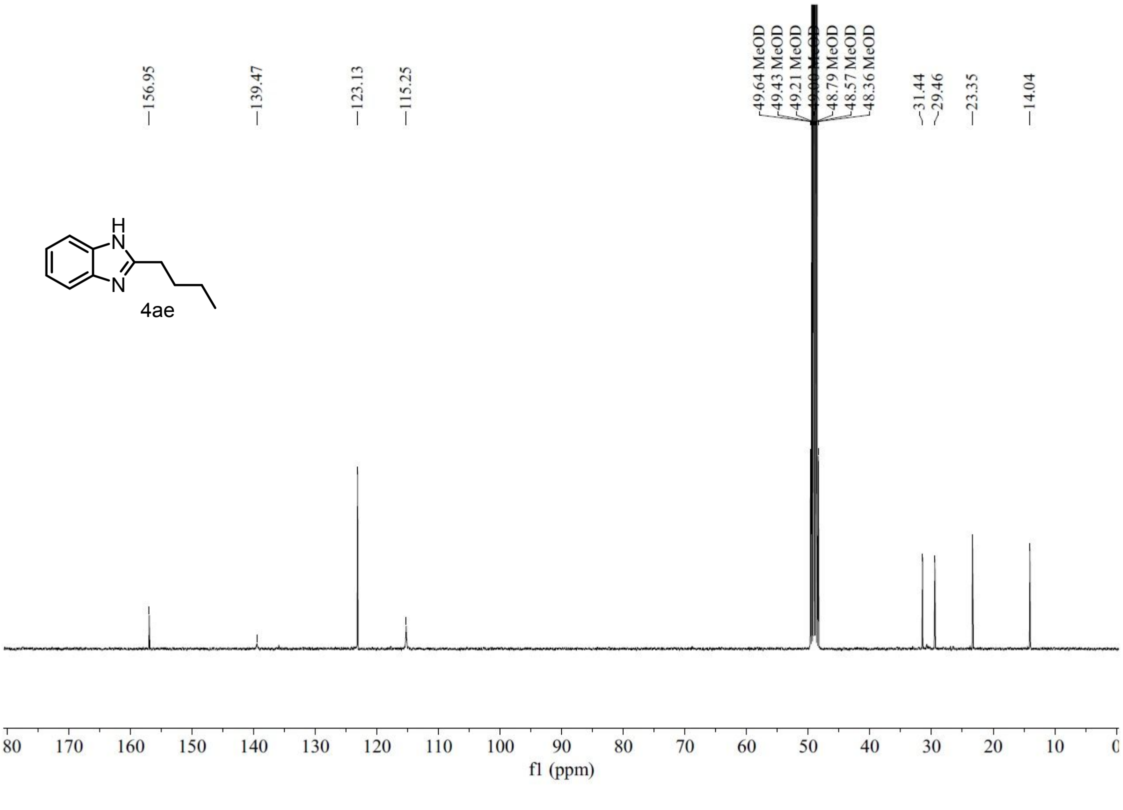
156.95
139.47
123.13
115.25
49.64 MeOD
49.43 MeOD
49.21 MeOD
48.79 MeOD
48.57 MeOD
48.36 MeOD
31.44
29.46
23.35
14.04
4ae
f1 (ppm)

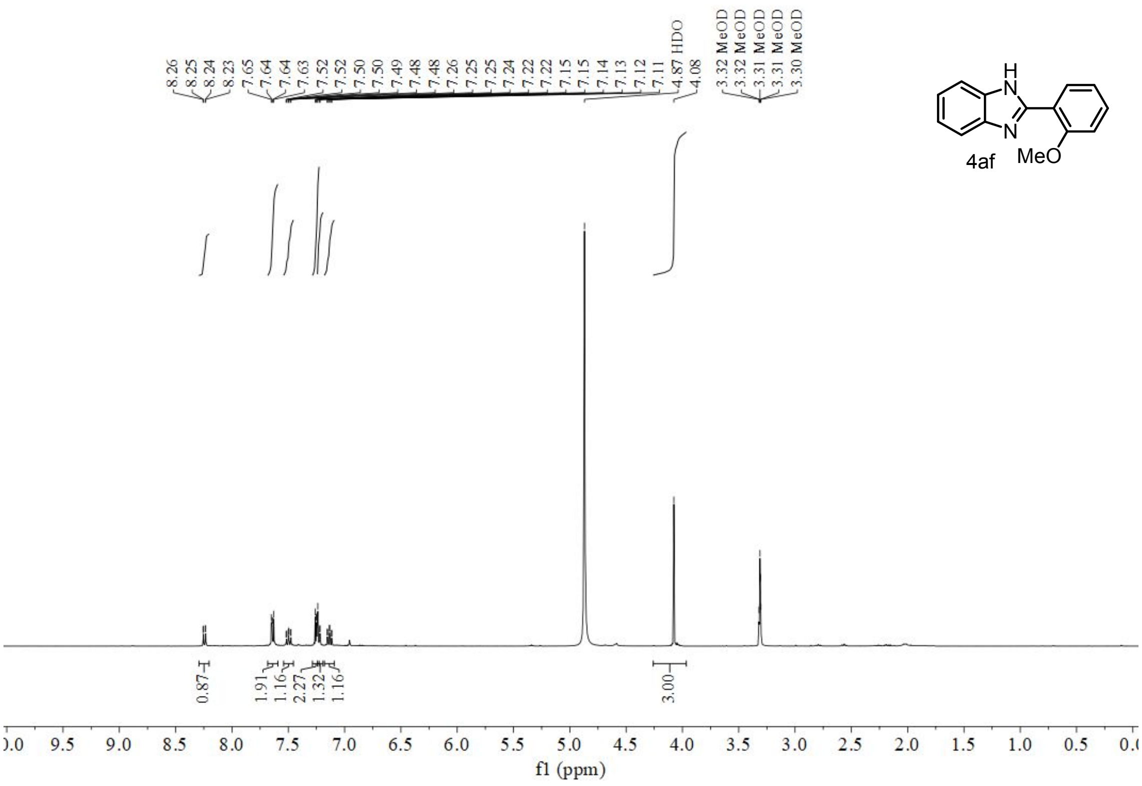
8.26
8.25
8.24
8.23
7.65
7.64
7.64
7.63
7.52
7.52
7.50
7.50
7.49
7.48
7.48
7.26
7.25
7.25
7.24
7.22
7.22
7.15
7.15
7.14
7.13
7.12
7.11
4.87 HDO
4.08
3.32 MeOD
3.32 MeOD
3.31 MeOD
3.31 MeOD
3.30 MeOD
H
N
N
4af
MeO
0.87
1.91
1.16
2.27
1.32
1.16
3.00
f1 (ppm)

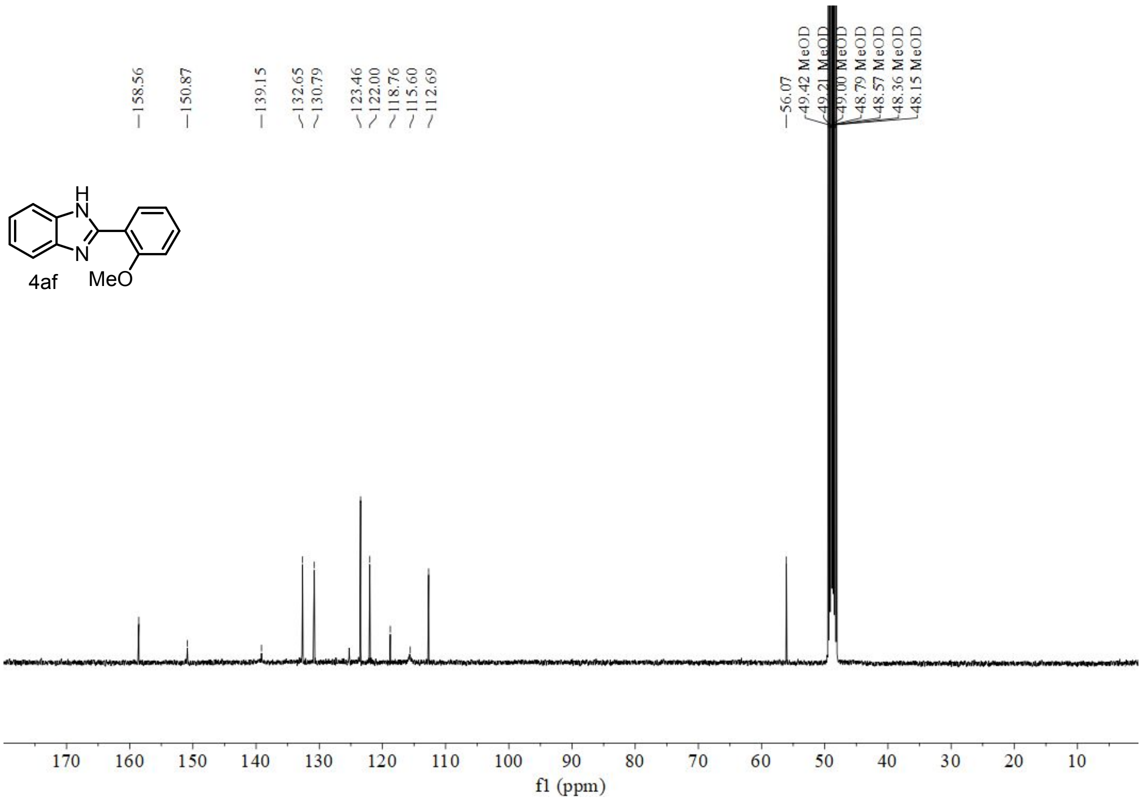
158.56
150.87
139.15
132.65
130.79
123.46
122.00
118.76
115.60
112.69
56.07
49.42 MeOD
49.21 MeOD
49.00 MeOD
48.79 MeOD
48.57 MeOD
48.36 MeOD
48.15 MeOD
H
N
N
4af
MeO
f1 (ppm)

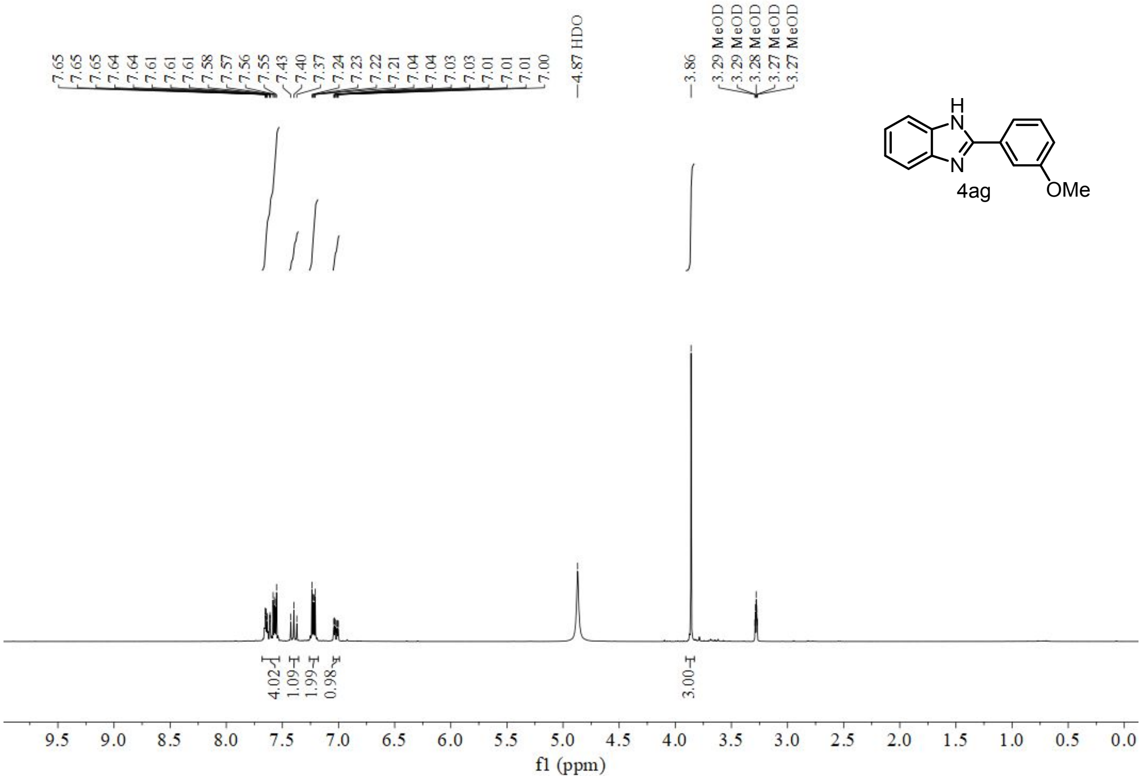
7.65
7.65
7.65
7.64
7.64
7.61
7.61
7.61
7.58
7.57
7.56
7.55
7.43
7.40
7.37
7.24
7.23
7.22
7.21
7.04
7.04
7.03
7.03
7.01
7.01
7.01
7.00
4.87 HDO
3.86
3.29 MeOD
3.29 MeOD
3.28 MeOD
3.27 MeOD
3.27 MeOD
4ag
OMe
4.02
1.09
1.99
0.98
3.00
f1 (ppm)

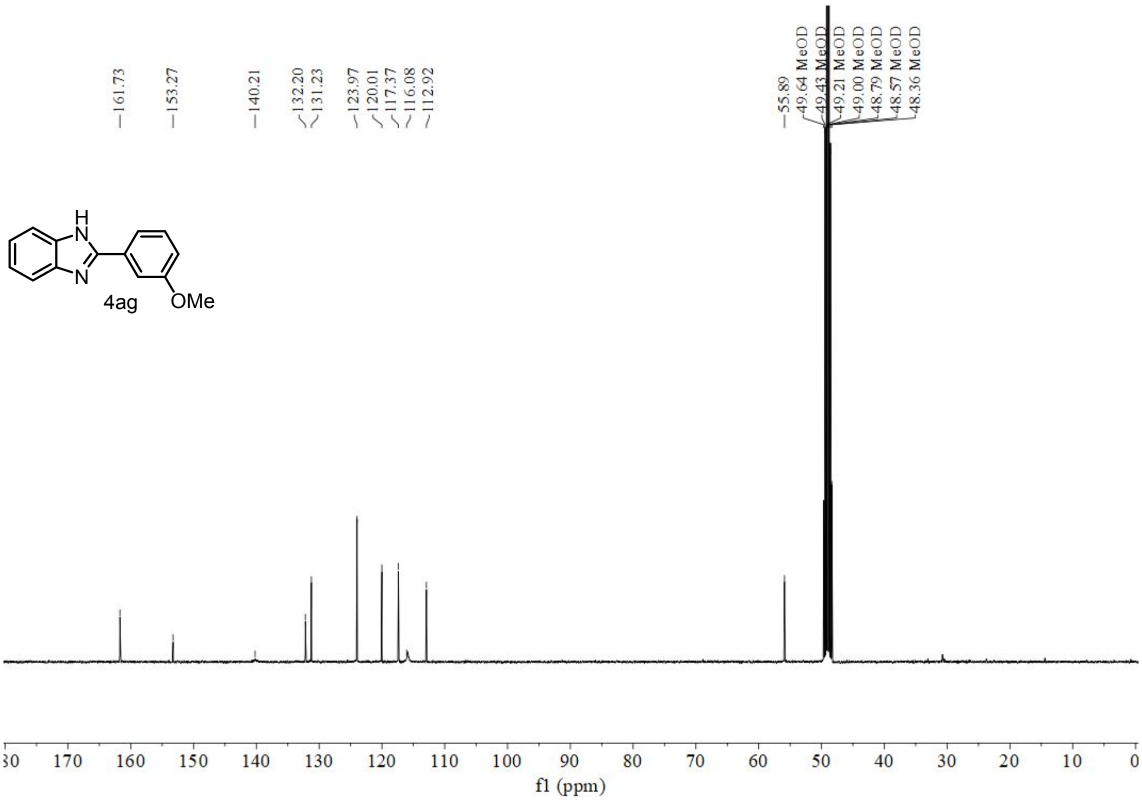
161.73
153.27
140.21
132.20
131.23
123.97
120.01
117.37
116.08
112.92
55.89
49.64 MeOD
49.43 MeOD
49.21 MeOD
49.00 MeOD
48.79 MeOD
48.57 MeOD
48.36 MeOD
4ag
OMe
f1 (ppm)

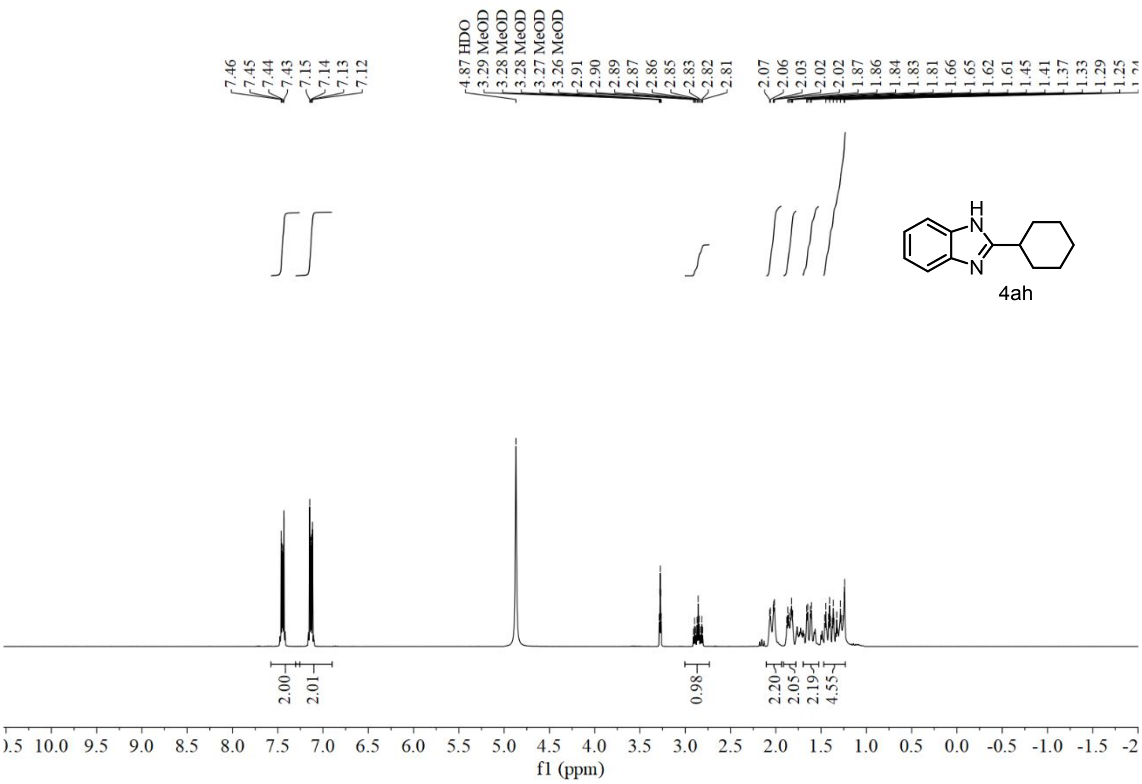
7.46
7.45
7.44
7.43
7.15
7.14
7.13
7.12
4.87 HDO
3.29 MeOD
3.28 MeOD
3.28 MeOD
3.27 MeOD
3.26 MeOD
2.91
2.90
2.89
2.87
2.86
2.85
2.83
2.82
2.81
2.07
2.06
2.03
2.02
2.02
1.87
1.86
1.84
1.83
1.81
1.66
1.65
1.62
1.61
1.45
1.41
1.37
1.33
1.29
1.25
H
N
N
4ah
2.00
2.01
0.98
2.20
2.05
2.19
4.55
f1 (ppm)

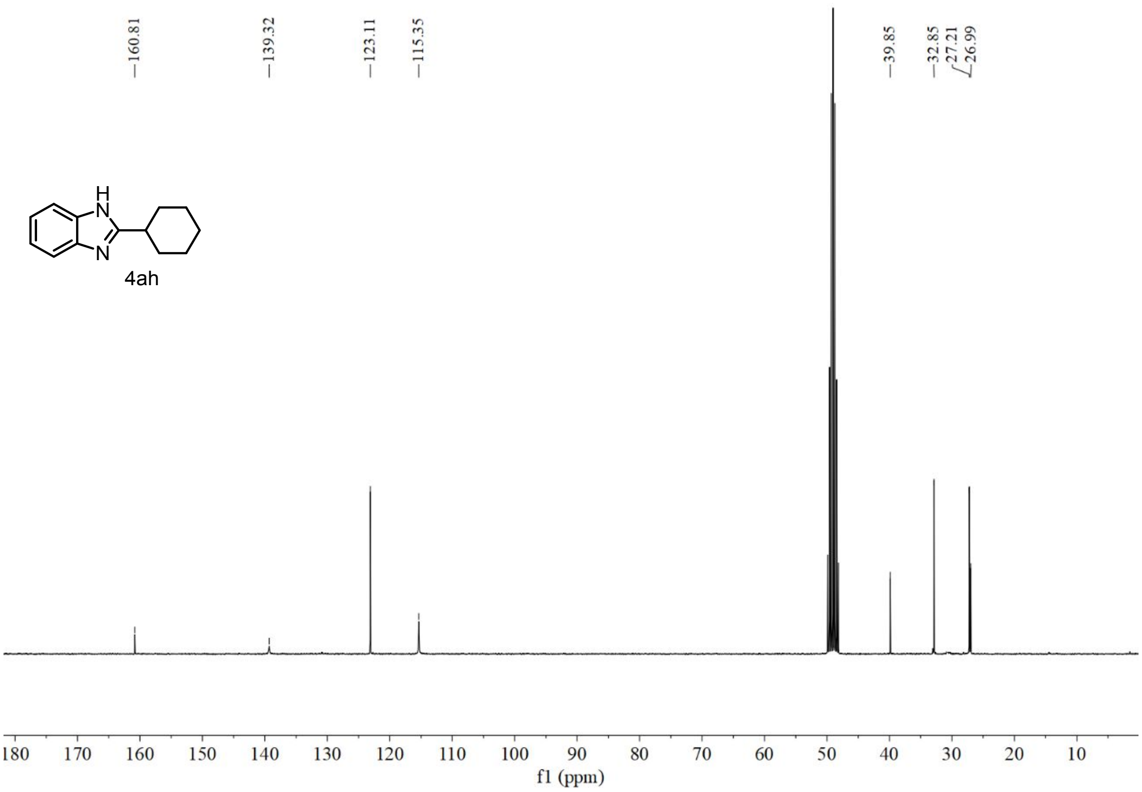
160.81
139.32
123.11
115.35
39.85
32.85
27.21
26.99
H
N
N
4ah
f1 (ppm)

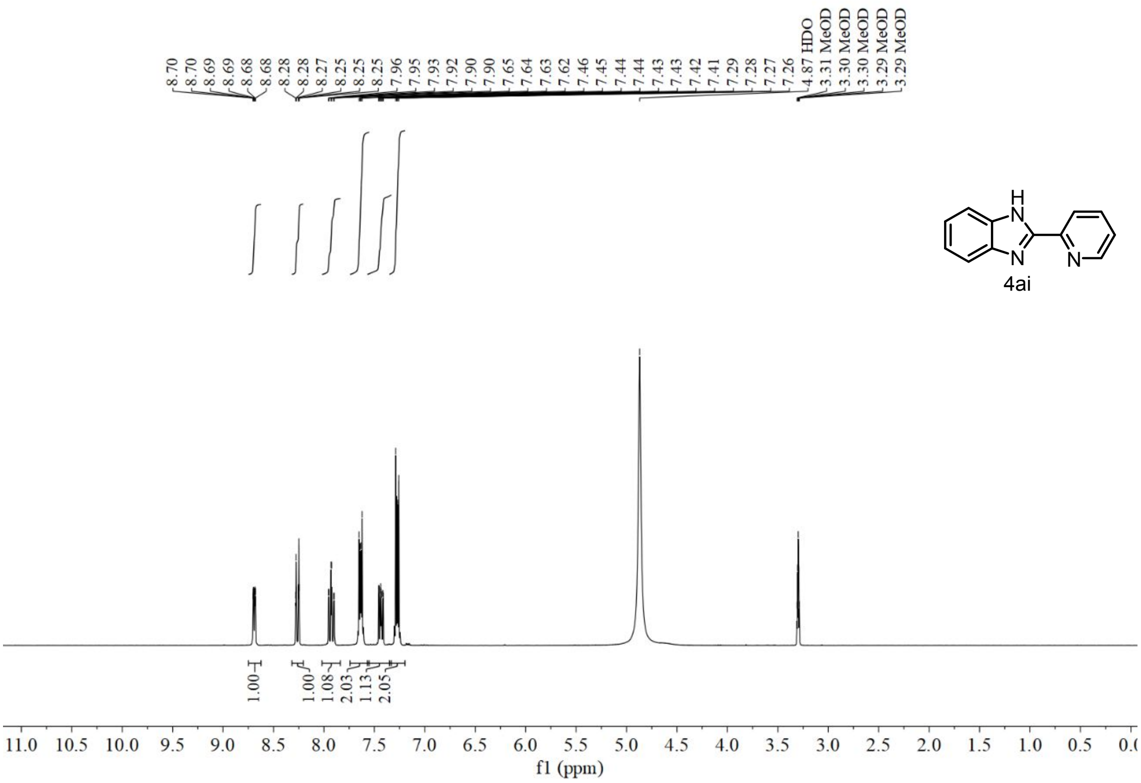
8.70
8.70
8.69
8.69
8.68
8.68
8.28
8.28
8.27
8.25
8.25
8.25
7.96
7.95
7.93
7.92
7.90
7.90
7.65
7.64
7.63
7.62
7.46
7.45
7.44
7.43
7.42
7.41
7.29
7.28
7.27
7.26
4.87 HDO
3.31 MeOD
3.30 MeOD
3.30 MeOD
3.29 MeOD
3.29 MeOD
H
N
N
N
4ai
1.00
1.00
1.08
2.03
1.13
2.05
11.0 10.5 10.0 9.5 9.0 8.5 8.0 7.5 7.0 6.5 6.0 5.5 5.0 4.5 4.0 3.5 3.0 2.5 2.0 1.5 1.0 0.5 0.0
f1 (ppm)

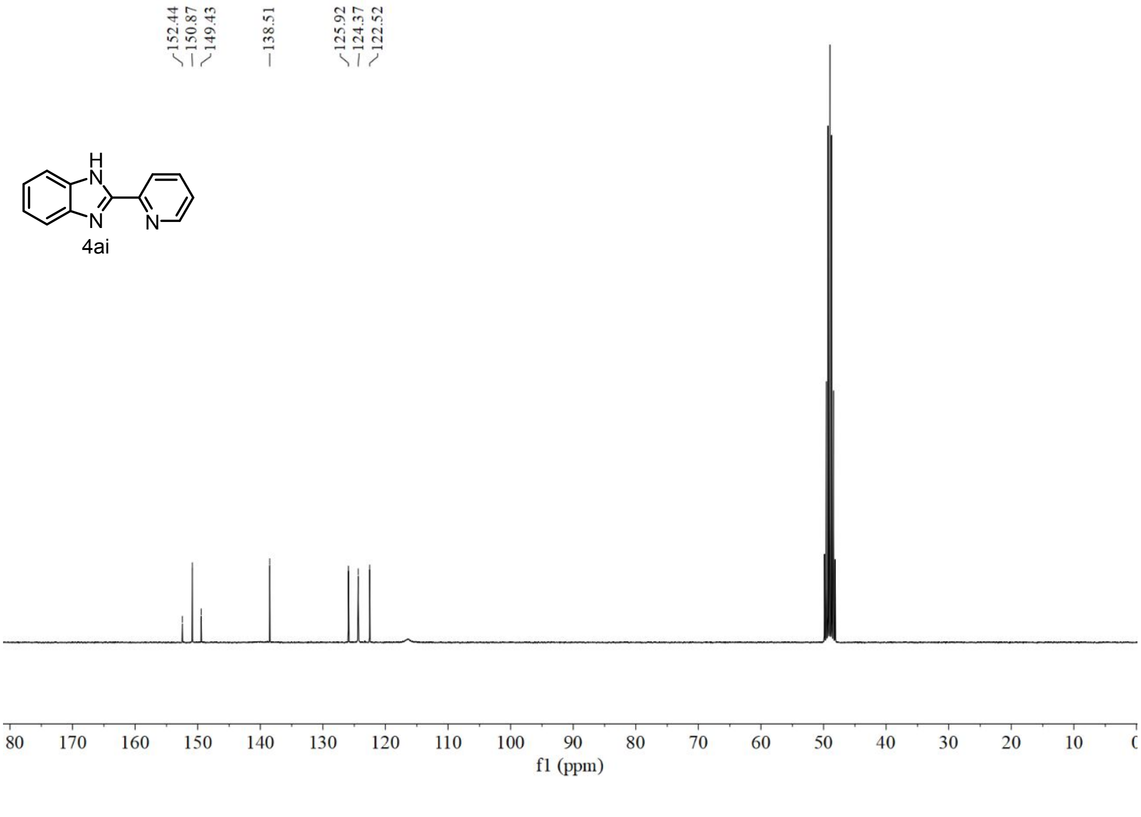
152.44
150.87
149.43
138.51
125.92
124.37
122.52
H
N
N
N
4ai
180 170 160 150 140 130 120 110 100 90 80 70 60 50 40 30 20 10 0
f1 (ppm)

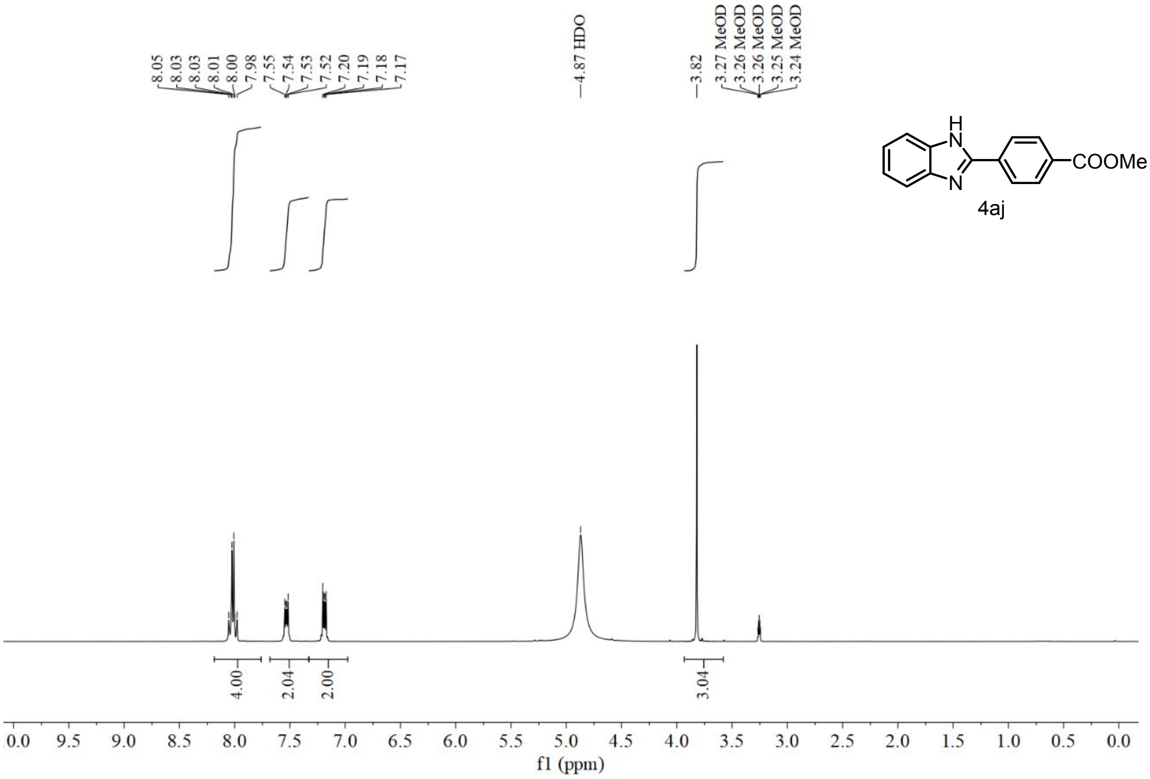
8.05
8.03
8.03
8.01
8.00
7.98
7.55
7.54
7.53
7.52
7.20
7.19
7.18
7.17
4.87 HDO
3.82
3.27 MeOD
3.26 MeOD
3.26 MeOD
3.25 MeOD
3.24 MeOD
COOMe
4aj
4.00
2.04
2.00
3.04
f1 (ppm)

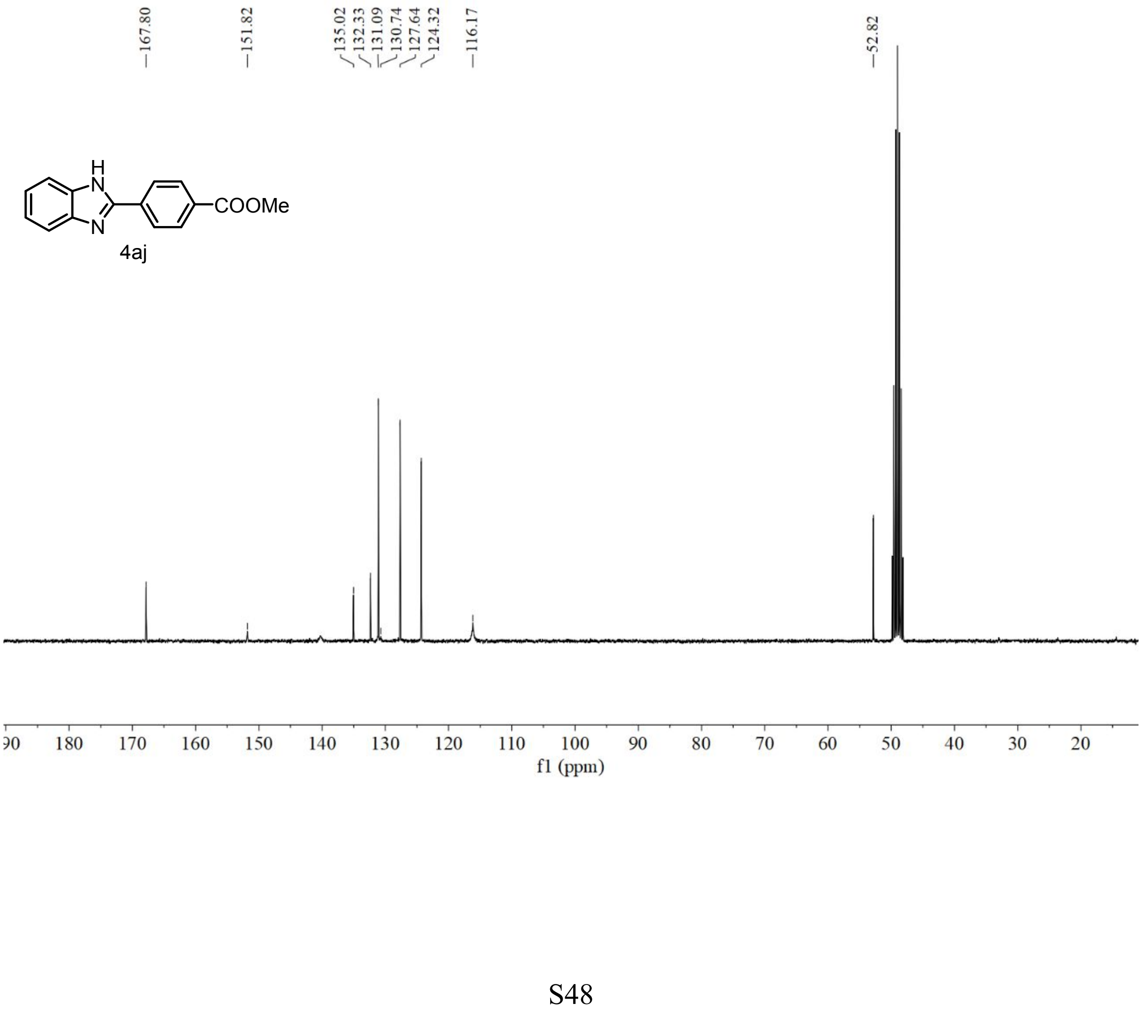
167.80
151.82
135.02
132.33
131.09
130.74
127.64
124.32
116.17
52.82
COOMe
4aj
f1 (ppm)

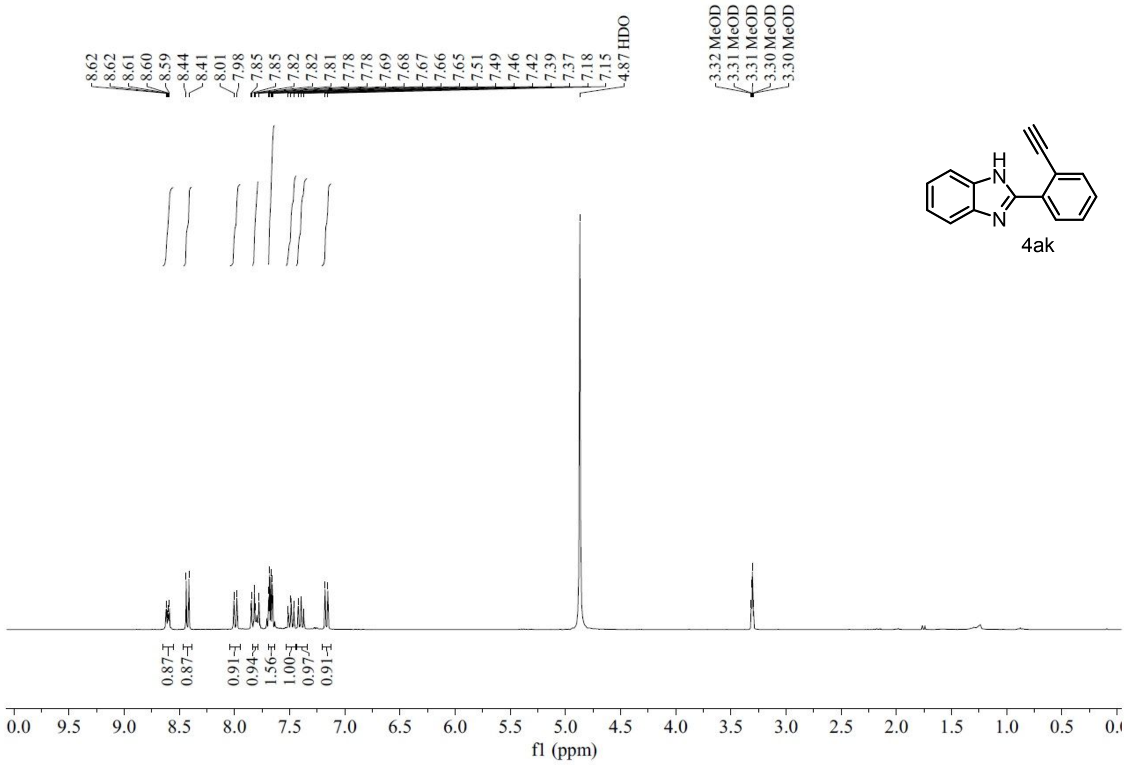

8.62
8.62
8.61
8.60
8.59
8.44
8.41
8.01
7.98
7.85
7.85
7.82
7.82
7.81
7.78
7.78
7.69
7.68
7.67
7.66
7.65
7.51
7.49
7.46
7.42
7.39
7.37
7.18
7.15
4.87 HDO
3.32 MeOD
3.31 MeOD
3.31 MeOD
3.30 MeOD
3.30 MeOD
H
N
N
4ak
0.87
0.87
0.91
0.94
1.56
1.00
0.97
0.91
0.0 9.5 9.0 8.5 8.0 7.5 7.0 6.5 6.0 5.5 5.0 4.5 4.0 3.5 3.0 2.5 2.0 1.5 1.0 0.5 0.
f1 (ppm)

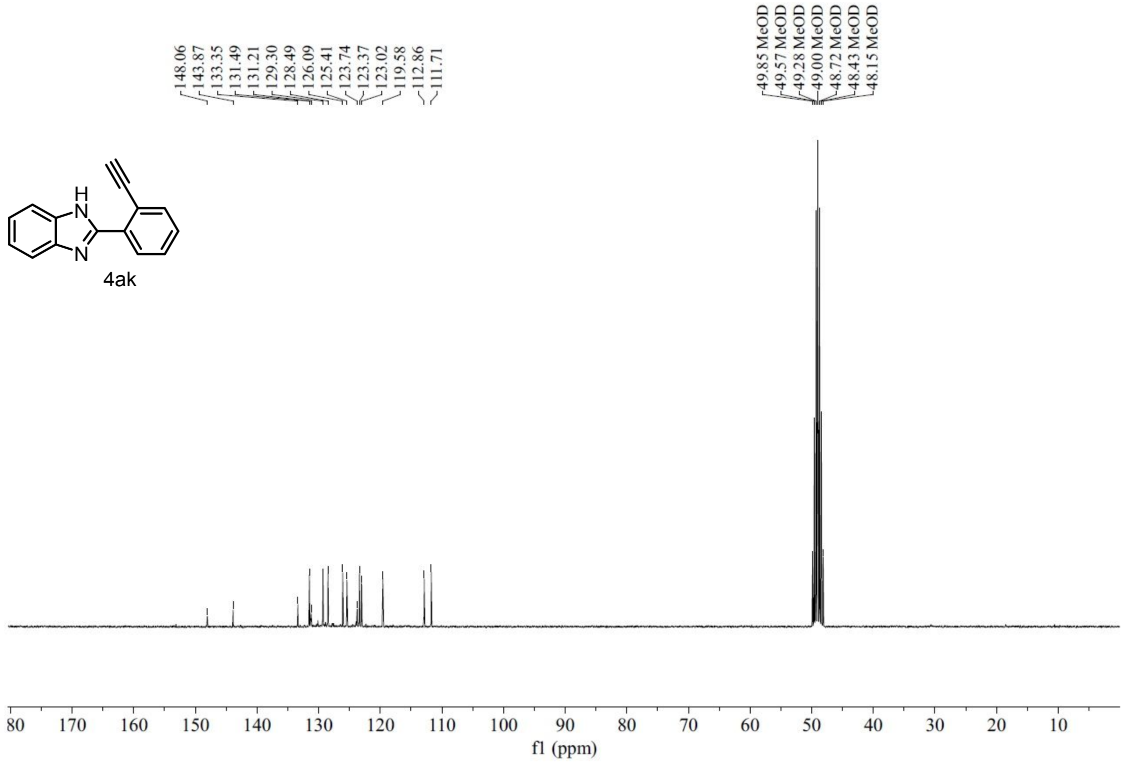

148.06
143.87
133.35
131.49
131.21
129.30
128.49
126.09
125.41
123.74
123.37
123.02
119.58
112.86
111.71
49.85 MeOD
49.57 MeOD
49.28 MeOD
49.00 MeOD
48.72 MeOD
48.43 MeOD
48.15 MeOD
H
N
N
4ak
80 170 160 150 140 130 120 110 100 90 80 70 60 50 40 30 20 10
f1 (ppm)

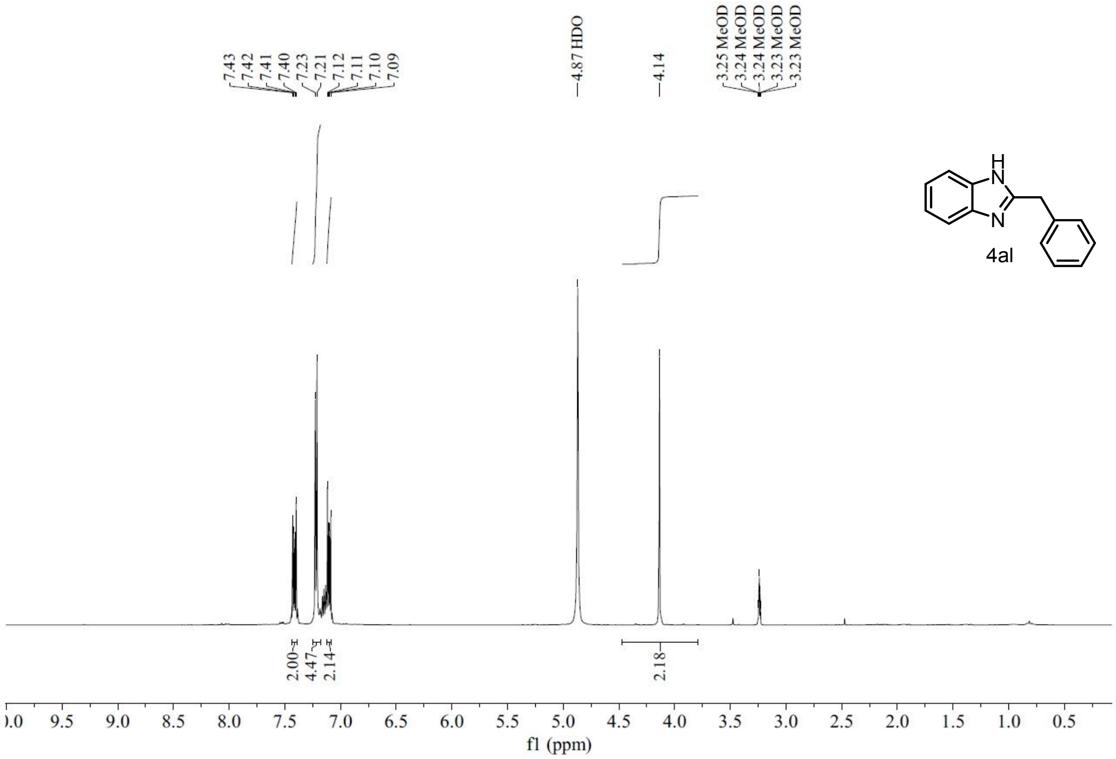
7.43
7.42
7.41
7.40
7.23
7.21
7.12
7.11
7.10
7.09
4.87 HDO
4.14
3.25 MeOD
3.24 MeOD
3.24 MeOD
3.23 MeOD
3.23 MeOD
4al
2.00
4.47
2.14
2.18
f1 (ppm)

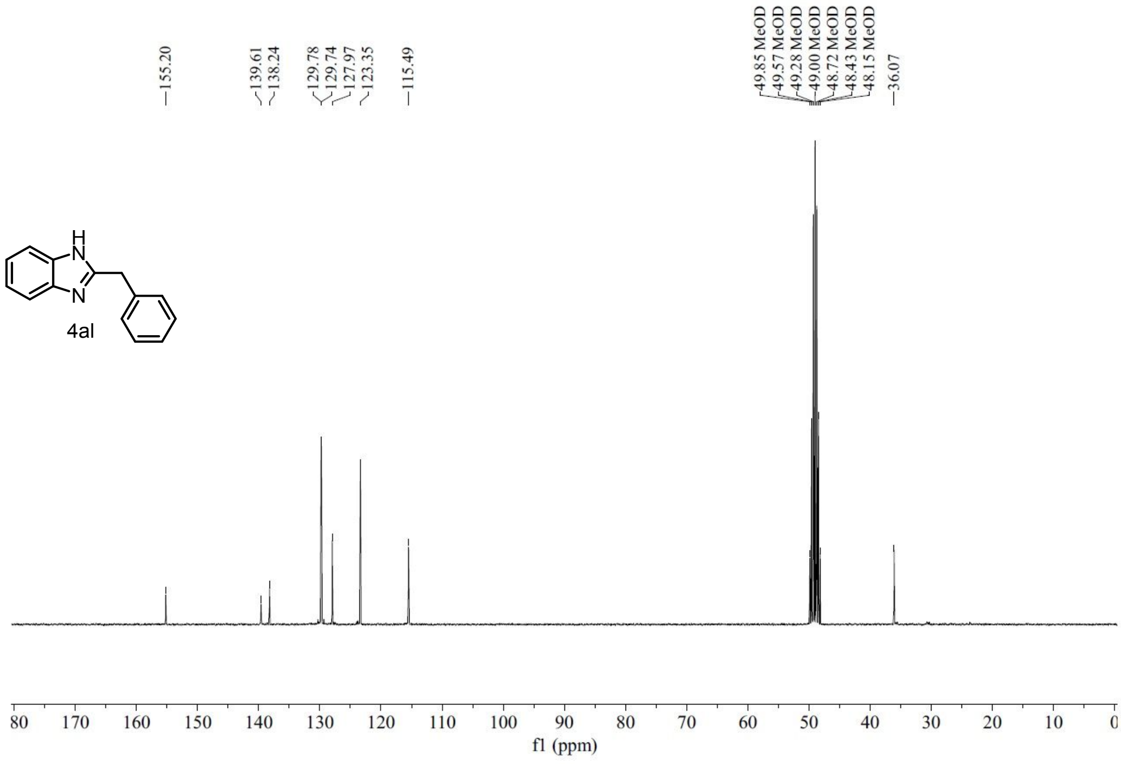
155.20
139.61
138.24
129.78
129.74
127.97
123.35
115.49
49.85 MeOD
49.57 MeOD
49.28 MeOD
49.00 MeOD
48.72 MeOD
48.43 MeOD
48.15 MeOD
36.07
4al
f1 (ppm)

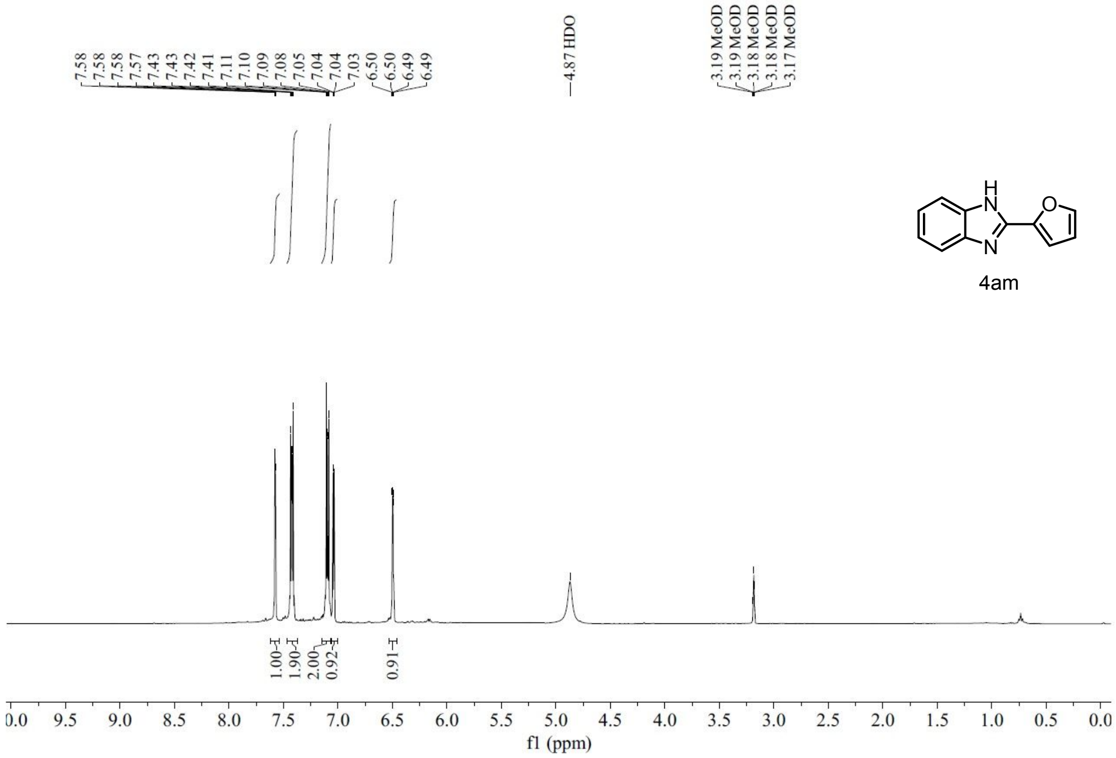
7.58
7.58
7.58
7.57
7.43
7.43
7.42
7.41
7.11
7.10
7.09
7.08
7.05
7.04
7.04
7.03
6.50
6.50
6.49
6.49
4.87 HDO
3.19 MeOD
3.19 MeOD
3.18 MeOD
3.18 MeOD
3.17 MeOD
4am
1.00
1.90
2.00
0.92
0.91
f1 (ppm)

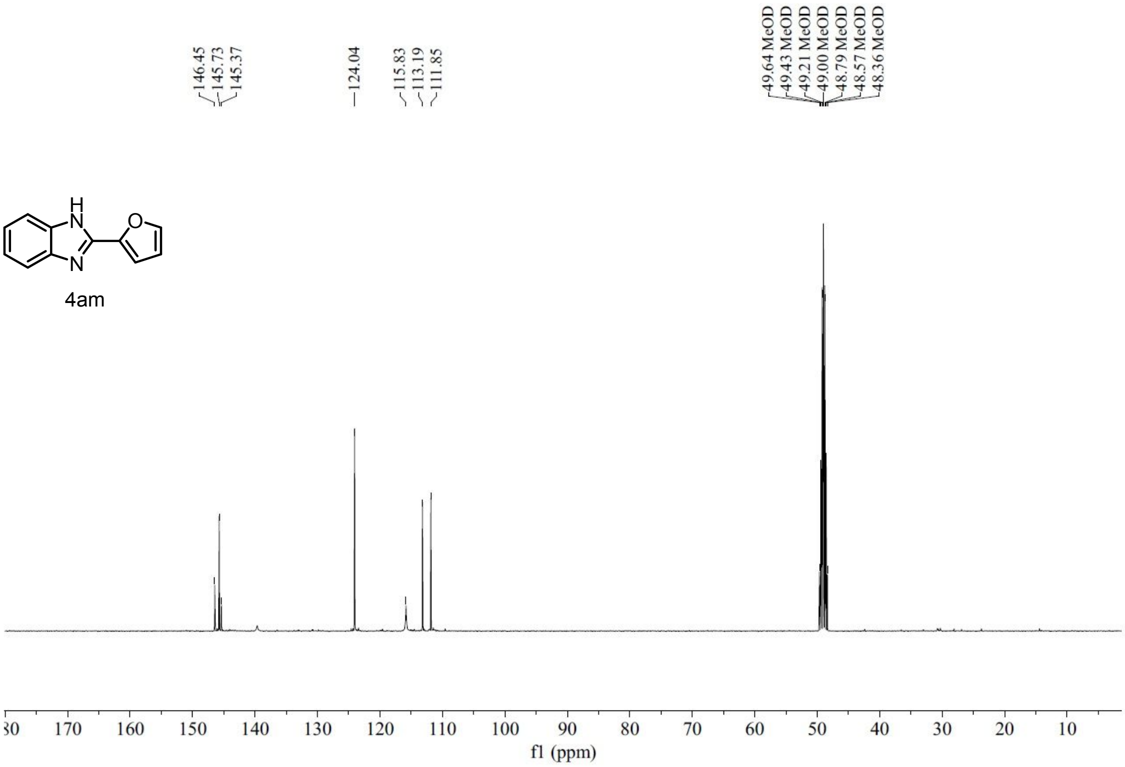
146.45
145.73
145.37
124.04
115.83
113.19
111.85
49.64 MeOD
49.43 MeOD
49.21 MeOD
49.00 MeOD
48.79 MeOD
48.57 MeOD
48.36 MeOD
4am
f1 (ppm)

9. References

(1) Ravel, B.; Newville, M. ATHENA ARTEMIS HEPHAESTUS: Data Analysis for X-Ray Absorption Spectroscopy Using IFEFFIT. *J. Synchrotron Radiat.* **2005**, *12*, 537–541. https://doi.org/doi:10.1107/S0909049505012719 537.

(2) Toby, B. H.; Von Dreele, R. B. GSAS-II: The Genesis of a Modern Open-source All Purpose Crystallography Software Package. *J. Appl. Crystallogr.* **2013**, *46*, 544–549. https://doi.org/doi:10.1107/S0021889813003531.

(3) Newville, M. Larch: An Analysis Package for XAFS and Related Spectroscopies. *J. Phys. Conf. Ser.* **2013**, *430*, 012007. https://doi.org/10.1088/1742-6596/430/1/012007.

(4) Sheldon, R. A. Metrics of Green Chemistry and Sustainability: Past, Present, and Future. *ACS Sustainable Chemistry and Engineering*. American Chemical Society January 2, 2018, pp 32–48. https://doi.org/10.1021/acssuschemeng.7b03505.

(5) Rodenes, M.; Gonell, F.; Martín, S.; Corma, A.; Sorribes, I. Molecularly Engineering Defective Basal Planes in Molybdenum Sulfide for the Direct Synthesis of Benzimidazoles by Reductive Coupling of Dinitroarenes with Aldehydes. *JACS Au* **2022**, *2* (3), 601–612. https://doi.org/10.1021/jacsau.1c00477.

(6) Abdi, B.; Tayebee, R.; Rezaei-seresht, E.; Zonoz, F. M.; Jalili, Z. TiO2/AgSbO3 Nanophotocatalyst with Improved Photocatalytic Performance in the Synthesis of Some Benzimidazole Derivatives. *J. Mol. Struct.* **2025**, *1321*, 140037. https://doi.org/10.1016/j.molstruc.2024.140037.

(7) Zhou, Y.; Zhang, Y.; Du, X.; Li, J.; Dong, C. Aerobic Oxidative Synthesis of N-Containing Heterocycles from O-Substituted Aniline Derivatives and Primary Amines by Perylene Diimide Catalysis. *European J. Org. Chem.* **2024**, *27*, e202400631.

(8) Luo, Q.; Dai, Z.; Cong, H.; Li, R.; Peng, T.; Zhang, J. Oxidant-Free Synthesis of Benzimidazoles from Alcohols and Aromatic Diamines Catalysed by New Ru(II)-PNS(O) Pincer Complexes. *Dalt. Trans.* **2017**, *46* (43), 15012–15022. https://doi.org/10.1039/c7dt02584j.

(9) Daw, P.; Ben-David, Y.; Milstein, D. Direct Synthesis of Benzimidazoles by Dehydrogenative Coupling of Aromatic Diamines and Alcohols Catalyzed by Cobalt. *ACS Catal.* **2017**, *7*, 7456–7460. https://doi.org/10.1021/acscatal.7b02777.
